# Novel inhibitors against *Cryptosporidium parvum* through blocking its energy metabolism

**DOI:** 10.1371/journal.pntd.0014742

**Published:** 2026-10-05

**Authors:** Chang Xu, Yaru Ji, Qiang Sha, Kun Li

**Affiliations:** 1 College of Veterinary Medicine, Nanjing Agricultural University, Nanjing, PR China; 2 College of Sciences, Nanjing Agricultural University, Nanjing, PR China; Universidade do Estado do Rio de Janeiro, BRAZIL

## Abstract

Through systematic structural optimization and modification of anti-*Cryptosporidium parvum* compound NSC158011 (N-1-naphthalenyl-2-(phenylthiol) ethane thioamide), we constructed a virtual compound library comprising 1,440 derivatives, and 29 derivatives were selected via molecular docking for chemical synthesis. Among them, eight derivatives exhibited significant inhibitory effects on LDH compared with NSC158011. In vitro examination found that two derivatives could markedly reduce the number of *C. parvum* in cells while exhibiting reduced toxicity. In vivo studies in model animals (IFN-γ knockout mouse) and native animals (calves) further validated the derivatives’ potent anti-parasitic effects and their ability to alleviate intestinal villus atrophy caused by parasitic infections. Further investigations revealed that compound treatment was associated with upregulated expression of intestinal tight junction proteins (ZO-1, Occludin, Claudin-1) and downregulated the expression level of inflammatory protein Caspase-1. Analysis of the gut microbiota and metabolomics in calves indicated that treatment significantly restored the abundance of beneficial flora and regulated key metabolites, including isocitric acid and ornithine. The two derivatives identified in this study represent promising lead compounds for further development as anti-cryptosporidiosis agents, with potential relevance to livestock production and public health.

## Author summary

In developing countries, cryptosporidiosis represents one of the leading causes of childhood diarrhea and growth retardation. However, there remains a lack of safe and effective therapeutic options in clinical practice. In this study, we optimized the structure of the lead compound NSC158011 to construct a virtual compound library comprising 1,440 derivatives. The candidate compounds with high affinity for the active site of lactate dehydrogenase (LDH) were screened out by molecular docking and binding free energy calculation for synthesis verification. Two potent antiparasitic derivatives were ultimately identified through high-throughput enzyme kinetics screening, cell assays and animal experiments conducted in two model systems. Further investigation revealed that this therapeutic approach may alleviate mitochondrial dysfunction and enhance energy metabolism and oxidative stress responses by restoring the function of the TCA cycle.

## Introduction

*Cryptosporidium* spp. is a protozoan parasite of many vertebrates worldwide, known to infect humans and various animals, causing gastroenteritis and diarrhea [1,2]. Neonatal calf diarrhea (NCD) is one of the most important concerns in cattle production [3]. Calf diarrhea is a highly prevalent clinical disease, with a mortality rate exceeding 8% in some regions [4]. The etiology of calf diarrhea is multifactorial, and among these factors, *Cryptosporidium*-induced diarrhea represents a significant challenge to the rearing of healthy calves [5]. Over the past decade, U.S. public health officials in 40 states and Puerto Rico have voluntarily announced 444 outbreaks of cryptosporidiosis, resulting in 7,465 cases and huge economic losses [6]. Clinically, Cryptosporidiosis in neonatal calves’ manifests as severe watery diarrhea, dehydration, and growth retardation. Mortality rates can reach 10–20%, and surviving calves often exhibit reduced weight gain and impaired feed conversion due to intestinal damage [7]. *C. parvum* is not only a critical pathogen in animal husbandry but also an important zoonotic pathogen that poses a threat to public health security. Globally distributed, cryptosporidiosis is closely associated with long-term diarrhea and high morbidity and mortality rates among children [8]. In the United States, *C. parvum* infection has become one of the leading causes of gastroenteritis. According to global multicenter studies on the gut, *C. parvum* ranks second after rotavirus as the primary cause of moderate-to-severe diarrhea in children across seven regions of South Asia and sub-Saharan Africa, with a relatively high risk of mortality for affected patients [9]. Currently, there are no vaccines specifically targeting cryptosporidiosis. Nitazoxanide is the sole US FDA-approved drug for treating cryptosporidiosis in immunocompetent adults and children over one year old. However, it is ineffective in immunodeficient individuals and exhibits significant side effects in cancer patients and organ transplant recipients [10,11]. Thus, there is an urgent need for novel, safe, and efficacious drugs to combat cryptosporidiosis.

Upon infecting the intestinal tract, *C. parvum* triggers a series of complex molecular pathological changes by disrupting the intestinal epithelial barrier function and activating the host’s immune response [12,13]. Oocysts detach from the host’s small intestine, releasing invasive sporozoites. These sporozoites preferentially colonize the brush borders of ileal epithelial cells, binding to host cell membrane receptors such as mucins and glycolipids via surface ligands (gp40/15) [14]. This process induces crypt hyperplasia, villous atrophy, and diffuse shortening or denudation of microvilli at the brush border, reducing the absorptive surface area and compromising barrier function [15]. Clinically, this manifests as severe osmotic diarrhea, dehydration, abdominal pain, malnutrition, and potential fatality in immunosuppressed patients [16]. *C. parvum* infection significantly downregulates the expression and distribution of tight junction proteins. ZO-1, a scaffold protein connecting transmembrane tight junction proteins (Claudins and Occludin) to the actin cytoskeleton, experiences reduced expression or mis-localization, disrupting intercellular junction stability and increasing paracellular permeability [17]. Claudin-1, which regulates tight junction pore size, shows decreased expression, weakening barrier selectivity and promoting abnormal permeation of water, electrolytes, and pathogens [18]. Occludin degradation further exacerbates barrier dysfunction, potentially mediated by proteases secreted by *C. parvum* cysteine protease or host matrix metalloproteinases MMPs [19]. Additionally, *C. parvum* infection activates the NLRP6 inflammasome, promoting Caspase-1 self-cleavage and activation. Activated Caspase-1 cleaves pro-IL-1β and pro-IL-18 into biologically active IL-1β and IL-18 [20], driving TH1-type immune responses while intensifying epithelial cell death and barrier disruption through GSDMD-mediated pyroptosis [21]

Unlike other Apicomplexa, *C. parvum* exhibits unique metabolic characteristics. It lacks a citric acid cycle and cytochrome-based respiratory chain in its mitochondria and does not possess an apicoplast [22], rendering traditional drug targets unsuitable for *C. parvum* treatment. Genomic sequencing reveals that although *C. parvum* retains residual mitochondria and encodes glycolysis-related enzymes, it lacks a tricarboxylic acid cycle and cytochrome-based respiratory chain [23]. Consequently, this parasite likely relies primarily on glycolysis for ATP production [24], making glycolytic enzymes attractive drug targets. Lactate dehydrogenase (LDH), a key enzyme in anaerobic respiration, exists in two main forms in humans. Studies indicate that LDH serves as a drug target for various parasitic protozoa, including *Plasmodium*, *Toxoplasma gondii*, *Giardia lamblia*, and *Trypanosoma cruzi*, as well as *Entamoeba histolytica* [25,26]. Despite high sequence conservation of LDH across organisms, structural and biochemical differences distinguish parasitic protozoan LDH, such as that of *C. parvum*, from human LDH. *C. parvum* LDH, a bacterial-type enzyme, shares only 25% amino acid sequence homology with human LDH [26,27], exhibiting distinct active and antigenic sites [28]. Previous research, using LDH knockout in *C. parvum*, confirmed that this enzyme is essential for parasite survival, viability, and virulence [29]. Zhang et al. first demonstrated that CpLDH is associated with the parasitophorous vacuole membrane during intracellular development and validated it as a potential target for therapeutic intervention. Cook et al. characterized the biochemical and structural properties of recombinant CpLDH, providing a structural basis for inhibitor design. Our prior work identified LDH as a key metabolic enzyme in *C. parvum* and NSC158011 as an LDH inhibitor with anti-*Cryptosporidium* activity [30]. In this study, we identified two novel compounds, H25 and H29, which demonstrate superior safety and efficacy compared to NSC158011. Molecular docking, enzymatic screening, cellular assays, and animal studies (in both mice and calves) collectively support the potent anti-*Cryptosporidium* effects of these compounds. These discoveries provide a novel framework for the development of anti-*Cryptosporidium* drugs.

## Materials and methods

### Ethics statement

All the experimental operations were under the instructions and approval of the Laboratory Animals Research Centre of Jiangsu, China, and the ethics committee of Nanjing Agricultural University (NJAU.No20250221020).

### Structure optimization and molecular docking simulation analysis

Based on the effective inhibition of compound NSC158011 against *C. parvum* lactate dehydrogenase (CpLDH) as reported in prior studies, the structural framework of the compound was retained. Based on the core scaffold of NSC158011, we performed systematic structural modifications using computer-aided drug design strategies (introducing electron-donating or electron-withdrawing substituents at different positions of the benzene ring and naphthalene ring (such as -OCH_3_, -Cl, -F, -CF_3_, etc.). Oxidize the thioether bond to form sulfoxide or sulfone. Introduce different-sized alkyl or aryl groups at the amide/sulfamoyl position, and construct a virtual compound library comprising 1,440 derivatives. Subsequently, molecular docking was performed to dock these virtual derivatives into the active pockets of CpLDH and H-LDH. Using the binding affinity of NSC158011 (-5.55 kcal/mol) as the benchmark threshold, combined with the requirement for low affinity to H-LDH, 722 candidate derivatives with theoretical scores comparable to or better than the parent compound were identified. Molecular docking simulations were conducted using AutoDock Vina Tools; the crystal structures of CpLDH (PDB: 4ND5) and H-LDH (PDB: 1I0Z) were obtained from the RCSB PDB database; the receptor protein was preprocessed through AutoDockTools with hydrogenation, calculation of Gasteiger charges, and addition of non-polar hydrogen, etc. The drug components were subjected to energy minimization using ChemDraw 3D. The number of connection operations was 15. Use pymol for visualization of docking. Compounds with binding affinity values against CpLDH more negative than or equal to that of NSC158011 and weaker binding affinity to H-LDH were selected as candidates. Derivatives exhibiting strong binding affinity to CpLDH (more negative than or equal to NSC158011) and weaker binding affinity to H-LDH were identified as potentially efficient and safe candidates. Binding affinity values were calculated using the AutoDock semi-empirical free energy scoring function and are expressed in kcal/mol. Importantly, more negative binding affinity values indicate stronger binding interactions between the compound and the target protein.

### Chemical synthesis methods of derivatives

In this study, 30 compounds (including the parent compound NSC158011 and 29 synthesized derivatives) were used and the numbers are shown as indicated in Fig 1C. Compounds 1, 15–17, 23–30 were synthesized according to route A. Route B was used to synthesize compounds 3, 4, 6, 11–14. Compounds 2, 5, 7–10, 18–22 were synthesized using route C.Route A: Under a nitrogen atmosphere, into a 500 mL three-necked round-bottom flask, naphthalen-1-amine (7.16 g, 50 mmol), triethylamine (7.60 mL, 55 mmol), and dichloromethane (DCM, 200 mL) were added with a stirring bar. The solution was cooled to 0–5 °C using an ice-water bath. Then, 2-bromoacetyl bromide (4.60 mL, 50 mmol) was added to the flask dropwise (about 10 minutes). Then the reaction mixture was allowed to warm to room temperature and stirred for 2 h. After adding 200 mL water to it, the mixture was extracted with DCM.

**Fig 1:**
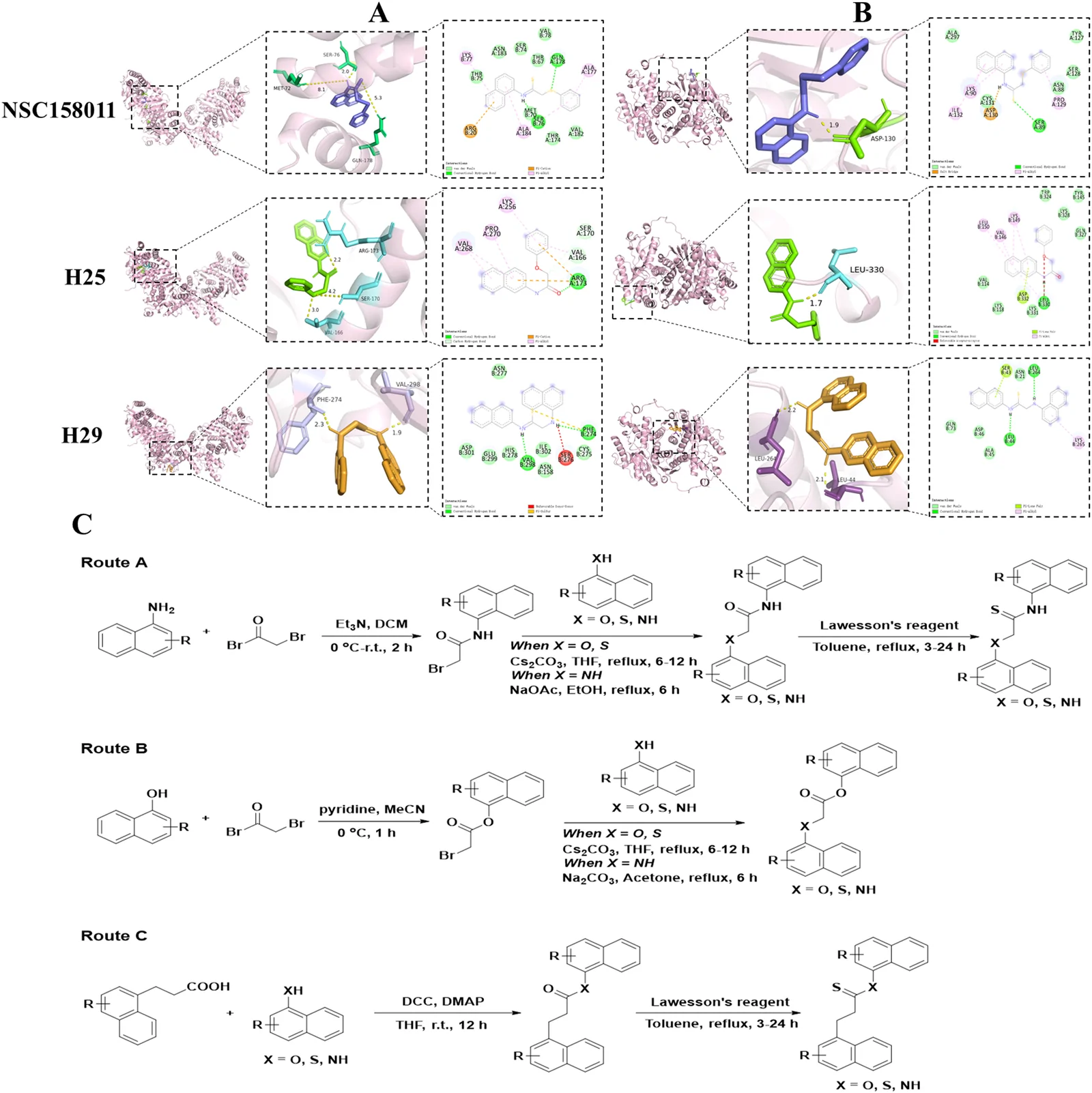
A: The molecular docking results of CpLDH with NSC158011 and the screening of effective derivatives. B: Molecular docking results of HLDH with NSC158011 and screening effective derivatives. C: Three Route maps for compound synthesis (Route A, Route B, Route **C)**. Abbreviations: CpLDH, *Cryptosporidium parvum* lactate dehydrogenase; H-LDH, human lactate dehydrogenase.

After this, the residue was twice washed with brine, dried, and filtered. The residue was purified after the removal of solvent for the production of the 2-bromo-N-(naphthalene-1-yl. In a 100 mL condenser-fitted flask, the mixture of benzenethiol, THFand 2- bromo-N- acetamide was addedCesium carbonate was added in the mixture, and heated for 6 hrs, then cooled, and the extraction was done 3 times with dichloromethane after adding water. A mixture of N-(naphthalen-1-yl)-2-(phenylthio) acetamide, Lawesson’s reagent, and toluene was placed in a 200 mL flask with a condenser. For three hours, the mixture was stirred at 110 °C, and then, using silica gel flash chromatography, the product was purified after removal of the solvent to produce N-(naphthalen-1-yl)-2-(phenylthio) ethane thioamide.

The solution was cooled to 0°C and added to a 100 mL round-bottom flask that contained naphthalen-1-ol (2.88 g, 20 mmol), pyridine (3.21 mL, 40 mmol), and acetonitrile (40 mL). Keeping the temperature at 0°C, 2.61 mL (30 mmol) of 2-bromoacetyl bromide was added dropwise over ten minutes. At this temperature, stirring continued for two hours. Dichloromethane (3 × 50 mL) was used to extract the reaction after it had been quenched with 100 mL of water. After being cleaned with saturated brine (2 × 50 mL), the mixed organic layers were dried over anhydrous Na_2_SO_4_ and filtered. Silica gel flash chromatography was used to separate naphthalen-1-yl 2-bromoacetat following solvent removal at low pressure. A 50 mL round-bottom flask was filled with a solution of naphthalen-1-yl 2-bromoacetate (528.0 mg, 2 mmol), aniline (186.0 mg, 2 mmol), and acetone (15 mL). Next, Na2CO3 (212.0 mg, 2 mmol) was added to the flask. For six hours, the reaction mixture was left to stir at 60 degrees Celsius. Naphthalen-1-yl phenylglycinate 11 was the result of purifying the residue that was left over after the solvent was evaporated under low pressure using silica gel flash chromatography. Route C: To a solution of 3-(naphthalen-1-yl) propanoic acid and naphthalen-1-amine in CH2Cl2 was added DMAP (4-dimethylaminopyridine, 0.122 g, 1.0 mmol) and DCC (dicyclohexylcarbodiimide, 2.27 g, 11.0 mmol) at room temperature. It was stirred for 12 hours at room temperature before being quenched with 1N HCl. After extracting the combination using CH2Cl2 (30 mL × 3), the mixed organic layers were then cleaned using brine and saturated NaHCO3 solution. Na2SO4 was used to dry the resulting organic layers that resulted.

After removal of solvent under reduced pressure, the residue was purified by silica gel column chromatography as eluent to give N,3-di(naphthalen-1-yl) propanamide. N,3-di(naphthalen-1-yl) propanamide (325 mg, 1 mmol), Lawesson’s reagent (243 mg, 0.6 mmol), and toluene (8 mL) were added to a 50 mL one-neck round-bottom flask equipped with a condenser, and the reaction mixture was allowed to stir at 110 °C for 3 h. The solvent was evaporated under reduced pressure, and the obtained residue was purified by silica gel flash chromatography, affording product N,3-di(naphthalen-1-yl) propane thioamide 5 (Fig 1C).

### Construction of LDH expression vector and protein purification

This study involved isolating a *C. parvum* strain to extract total RNA, synthesize cDNA, and perform reverse transcription. Using specific primers (forward: 5’-CTCGAGATGATTGAAAGACGCAAGA-3’; reverse: 5’-GGATCCTTATGCTCCAGCTGGT-3’), CpLDH gene coding sequence the coding sequence of the CpLDH gene was amplified. The PCR product was cloned into the pET28a expression vector via XhoI/BamHI double restriction sites, constructing an N-terminal hexahistidine (His-tag) fusion expression system, which was subsequently verified by sequencing. Following transformation of the recombinant plasmid into *E. coli* BL21(DE3), single colonies were selected and inoculated in LB liquid medium containing selection antibiotics. Cultivation was performed at 37°C until the OD_600_ reached 0.6–0.8. Expression optimization was conducted using 0.8 mM and 1.0 mM IPTG at varying temperatures (28°C and 37°C) and durations (0–6 hours). Bacterial cells were lysed by ultrasonic disruption (5 seconds on/off, total duration 3 minutes), and the supernatant and precipitate were separated by centrifugation. Optimal expression conditions were determined through 12% SDS-PAGE analysis.

Under optimal conditions, the protein was expressed on a large scale. After bacterial lysis with PBS buffer (containing PMSF), the supernatant was subjected to ultrasonic disruption, and the protein was purified using nickel affinity chromatography. The chromatographic column was washed with five column volumes of ddH_2_O, and the flow-through and gradient elution fractions (20–500 mM imidazole) were collected for sampling. Optimal elution conditions were identified via SDS-PAGE analysis, yielding highly purified recombinant CpLDH protein.

### Protein-level inhibitory effect

An enzymatic assay was established based on the purified recombinant protein. The reaction system comprised 10 mM pyruvate, 1 mM NADH, and 100 mM Tris-HCl buffer (pH 7.5), with varying concentrations of recombinant CpLDH protein added. A negative control group without added protein was included. Absorbance changes were monitored every 15 seconds at 340 nm wavelength over 2 minutes using an enzyme-labeled reader, enabling determination of enzyme kinetic parameters. This system facilitates high-throughput screening of compounds that inhibit the catalytic reduction of pyruvate to lactic acid by CpLDH.

### Cytotoxicity of NSC158011 and its derivatives

Derivatives exhibiting inhibitory effects on recombinant CpLDH were re-screened, and those demonstrating superior inhibitory activity compared to NSC158011 were pre-tested for cytotoxicity. The effective derivatives were subsequently evaluated three times using the WST-1 cell proliferation assay. Cryopreserved HCT-8 cells were rapidly thawed in a 37°C water bath and cultured under standard conditions. Experiments were conducted after two to three passages of cell culture. HCT-8 cells were seeded in 96-well plates with 200 μL of phenol red-free RPMI 1640 medium (Solaibao Technology Co., LTD), supplemented with 10% fetal bovine serum (Gibco, USA) and 1x penicillin-streptomycin-amphotericin B solution (Solaibao Technology Co., LTD). Upon reaching confluence, the medium was replaced with fresh medium containing varying concentrations of test compounds. A control group without compounds was established, with three replicates per concentration. After incubation for 24 hours, 20 μL of the WST-1 cell proliferation reagent (Biyuntian Biotechnology Co., LTD) was added to each well, followed by mixing and incubation at 37°C with 5% CO_2_ in the dark for 14 hours. Subsequently, 100 μL of the medium from each well was transferred to a new 96-well plate, and absorbance was measured at 450 nm using a scanning multi-well spectrophotometer.

### Parasite purification and excystation

In this study, *C. parvum* were utilized and maintained through passage in male Holstein calves. For the extraction and purification of oocysts, calf fecal samples were sequentially sieved and filtered. Subsequently, purification was performed using the Sheather sugar flotation method followed by discontinuous sucrose density gradient centrifugation(67). After being kept at 4 °C in a 50 mM tris buffer with 10mM EDTA (pH 7.2) this purified oocysts were used. In 500 µL of PBS oocyst were suspended and mixed with 40% commercial bleach with equal volume and to induce excystation the suspension was kept at 4°C. The mixture was resuspended in Hanks’ balanced salt solution, incubated for one hour at 37°C, and then rinsed four times with PBS supplemented with 1% bovine serum albumin. Subsequently, an equal volume of 1.5% sodium taurocholate was added. Sporozoites released from the oocysts were pelleted by centrifugation, washed, resuspended in PBS, and further purified using a 5 µm sterile syringe filter (Millex). Sporozoite counts were determined using a hemocytometer.

### Immunofluorescence and RT-qPCR quantitative analysis of the in vitro inhibitory effect of NSC158011 and its derivatives on *Cryptosporidium parvum*

For in vitro screening of compound activity against *C. parvum*, assays were performed at concentrations not exceeding 50% of the compound’s cytotoxic IC_50_. HCT-8 cells were seeded into 96-well plates and maintained in RPMI-1640 medium supplemented with 10% fetal bovine serum. Once the cells reached full confluence, the culture medium was removed and replaced with fresh complete medium for subsequent experiments. The experimental Settings included: three concentration groups for the derivative to be tested (0 concentration control group, 0.5 times IC_50_ concentration group and IC_50_ concentration group) with each concentration set up in triplicate. A blank control group without any inhibitor was included. The positive control was treated with paromomycin (a clinical anti-*cryptosporidium* drug) reconstituted in sterile distilled water. The HCT-8 cell culture wells were each inoculated with 4 × 10⁴ of freshly purified *C. parvum* sporozoites and incubated in a 5% CO_2_ incubator at 37°C for 48 hours. After incubation, the culture supernatant was decanted, and the cells fixed with methanol-acetic acid (9:1, v/v) at room temperature for 2 minutes. After fixation, the cells were washed twice with osmotic buffers containing 0.1% Triton X-100, 0.35M NaCl and 0.13M Tris-base (pH 7.6) to fully rehydrate and permeate. The nonspecific antibody binding was blocked with 5% normal goat serum, and commercial anti-*Cryptosporidium* antibody (SporoGlo) was to the cells and incubated overnight at 4°C in the dark. The cells were washed twice with phosphate buffer (PBS), then once with deionized water, and finally observed and images acquired under an inverted fluorescence microscope. The fluorescence intensity was quantitatively analyzed by ImageJ (1.37v). All experiments were set up with 3 technical replicates, and the experiment was independently repeated at least 3 times.

### Testing compounds’ in vivo anti-cryptosporidium efficacy using an IFN-γ knockout mouse and a bovine calf model

In this study, two animal models were used to evaluate the efficacy of compounds against *C. parvum*, including gamma-interferon-knockout mice (B6.129S7-Ifng, evenly divided by sex, aged 4 weeks) and neonatal bovine calves. NSC158011 and its derivatives H25 and H29 were reconstituted in DMSO and subsequently diluted with sterile distilled water to achieve a final DMSO concentration of less than 1% (v/v). A pre-experiment test was conducted to determine the maximum tolerable dose using a dose range of 0–1000 mg/kg and monitoring toxic reactions in both mice and calves for seven days. These reactions included activity, respiration, body temperature, and other relevant clinical indicators. The highest tolerable doses for NSC158011, H25, and H29 were determined to not exceed 400, 800, and 800 mg/kg, respectively.

The study design included six groups (n = 3 per group): normal control (CS), infection control (MS), paromomycin treatment (PS at 50 mg/kg), and three compound treatment groups (H23S, H25S, H29S, all at 50 mg/kg). Additionally, another set of six groups (n = 3 per group) was established: normal control (CR), infection control (MR), halosterone treatment (CSTR at 25 mg/kg), and three compound treatment groups (H23R, H25R, H29R, all at 25 mg/kg). On the first day of the experiment, mice were inoculated with 3000 *C. parvum* oocysts, while calves received 50,000 oocysts. Compound administration commenced on the following day and continued for seven consecutive days. Feces were collected daily in PBS supplemented with antibiotics (penicillin 100 U/mL, streptomycin 100 μg/mL) and stored at 4°C for subsequent DNA analysis. On day 9, the animals were euthanized, and intestinal tissue was harvested and preserved for further analysis. Throughout the experiment, animals were housed separately, and the control group received an equivalent volume of sterile distilled water containing an equivalent volume of DMSO as in compound-treated groups.

### Histopathological analysis of intestinal tissues

The jejunum and ileum of mice and calves were collected and preserved in 4% paraformaldehyde. The intestinal tissue was submitted to Wuhan Pinofei Biotechnology Co., LTD for histological preparation and analysis. Briefly, the intestinal tissue preserved in 4% paraformaldehyde was washed with 70% ethanol and then deposited in 1% AGAR, followed by paraffin inclusion. The paraffin-embedded tissue was sectioned transversely into 5 μm sections and stained with Hematoxylin-Eosin according to standard procedures of the veterinary diagnostic laboratory. Sections were imaged using a Zeiss microscope, and images were captured with a color camera.

### Quantitative determination of *Cryptosporidium parvum* in feces of experimental animals

Genomic DNA was extracted from individual fecal samples collected from laboratory animals on different days. A fecal DNA extraction kit was used purify genomic DNA from 220 mg of homogenized fecal sample following the manufacturer’s instructions (Beijing Solaibao Technology Co., LTD.). Primers targeting the *C. parvum* 18S rRNA gene (5 ‘-CTGCGAATGGCTCATTATACA-3’, Forward, and 5 ‘-AGGCCAA-TACCCTACCGCTT-3’, Reverse) were used to amplify a 240 bp gene fragment from the fecal genomic DNA by conventional PCR. The PCR product was fractionated on agarose gel, and the target gene fragment using a gel extraction kit (Nanjing Nuovizan Biotechnology Co., LTD.), and the concentration of the product determined by Nanodrop spectrophotometer (Fisher, USA). The extracted DNA fragments were 10-fold serially diluted to generate quantitative standards for generating the standard curve by RT-qPCR. For quantitative RT-qPCR system, a reaction mixture consisted of 5 μl DNA template, 1 μL primer mixture (500 nM each), 10 μL ChamQ SYBR Color qPCR Master Mix (Nanjing Nuoweizan Biotechnology Co., LTD.), and the final volume made up to 20 μL with sterile molecular grade water. qPCR reaction conditions were as follows: pre-denaturation at 95 ° C for 30 s, 40 cycles of denaturation at 95˚C for 10 s, annealing at 60˚C for 30 s, and a final melting curve generation step using the instrument’s default melting curve acquisition procedure (LightCycler 96).

### Analysis of genes and proteins related to intestinal barrier and inflammation

According to the RNA sequences of Claudin-1, Occludin, Caspase-1, and ZO-1 in the mouse and calf databases from GenBank, specific primers were designed using Primer Premier 6.0 software (S2 and S3 Tables) and subsequently validated against the GenBank database. The optimal primer sequences were selected, and all primers were synthesized by Shanghai Shenggong Biological Engineering Co., Ltd. Middle portions of the jejunum and ileum tissues (25 mg each) were collected from experimental animals in each group. Total RNA from intestinal tissues and cells were extracted according to the manufacturer’s instructions using an RNA extraction kit (Ecoray Biotechnology Co., Ltd.). Subsequently, cDNA was synthesized via reverse transcription following the protocol provided with the HiScript III All-in-one RT SuperMix Perfect for qPCR kit (Nanjing Nuoweizen Biotechnology Co., Ltd.). Using cDNA as the template and β-actin as the internal reference, fluorescence quantitative PCR analysis was performed on the gene expression levels of Claudin-1, Occludin, Caspase-1, and ZO-1 in intestinal tissues using the TSINGKE TSQ0101 ArtiCanATM SYBR qPCR Mix (Low ROX Premixed) (Beijing Qingko Biotechnology Co., Ltd.). The RT-qPCR reaction system consisted of 10 μL of 2xSYBR qPCR Mix, 1 μL of primer mixture (each at 500 nM), and 1 μL of cDNA template in a total volume of 20 μL. The qPCR reaction conditions were as follows: initial denaturation at 95°C for 20 s, followed by 40 cycles of denaturation at 95°C for 3 s and annealing/extension at 60°C for 10 s. Melting curve analysis was conducted using the default program of the LightCycler 96 instrument.

The total protein of jejunum and ileum of calves was extracted from RIPA lysate (containing protease inhibitor). The concentration of each sample supernatant was determined using a commercial BCA protein detection kit (Solebol, China). Samples were prepared by mixing 5 × loading buffer with protein samples (1:4 ratio) and boiling the mixture at 95–100°C for 5 min. The samples were fractionated by sodium dodecyl sulphate-polyacrylamide gel electrophoresis (SDS-PAGE), and transferred to 0.45 μm polyvinylidene fluoride (PVDF) membrane. The membranes were submerged in 5% skim milk at room temperature for 2 h, and incubated overnight at 4 °C with primary antibodies against GAPDH, Occludin, Cleaved Caspase-1, Claudin-1 or ZO-1 (ABclonal). After washing with TBST (containing Tween-20), the membranes were incubated with horseradish peroxidase (HRP)-conjugated secondary antibody at 37°C for 1 hour. Finally, signal generation was done by using an enhanced chemiluminescence (ECL) reagent (Soleibao, China). Protein band signals were analyzed using ImageJ software (NIH Image, USA), and the relative expression levels of GAPDH and target proteins (Occludin, Cleaved Caspase-1, Claudin-1, and ZO-1) were normalized with the GAPDH signal.

### Elisa detection of serum inflammatory related factor

Blood samples were obtained from the orbital venous plexus of mice and the jugular vein of calves. Whole blood was allowed to clot at room temperature for 1 hour, followed by centrifugation at 4000 rpm for 10 minutes to separate the serum. Levels of interleukin-6 (IL-6), interleukin-10 (IL-10), interleukin-1β (IL-1β), and tumor necrosis factor-α (TNF-α) were quantified using ELISA kits (Solarbio Life Sciences) according to the manufacturer’s protocols.

### Intestinal microbiome and metabolome sequencing and bioinformatics analysis

Total genomic DNA was isolated from rectal fecal samples collected from each group (n = 3 for mice; n = 6 for calves) using a commercial fecal DNA extraction kit. DNA quality and concentration were evaluated by 1.2% agarose gel electrophoresis and quantified with a NanoDrop 2000 spectrophotometer (Thermo Scientific, USA). The V3–V4 region of the bacterial 16S rRNA gene was amplified using primers 338F and 806R, as described in previous studies. PCR products were confirmed by 1.5% agarose gel electrophoresis and purified with the GenElute Gel Extraction Kit (Sigma-Aldrich, Germany). Sequencing libraries were prepared, quantified using the QuantiFluor-ST system (Promega, USA), and sequenced on an Illumina MiSeq platform.

In the bioinformatics analysis, QIIME2 was employed to perform quality control on the raw sequencing data and generate amplicon sequence variants (ASVs). These ASVs were then compared against the Greengenes database for taxonomic annotation. Microbial diversity metrics, including alpha-diversity and beta-diversity, were comprehensively analyzed in the C57BL/6 mouse cohort. To elucidate differences in microbial community structures across animal populations, random forest analysis and partial least squares discriminant analysis (PLS-DA) were utilized. Significant differences in bacterial abundances between populations were identified using statistical methods such as one-way ANOVA and DESeq2.

For metabolomics profiling, the intestinal contents of calves were subjected to non-targeted metabolomics analysis using ultra-high performance liquid chromatography coupled with tandem mass spectrometry (UHPLC-ESI-Q-Exactive HF-X). Chromatographic separation was achieved on an ACQUITY UPLC BEH C18 column (1.7 μm particle size, 2.1 mm × 100 mm). Data preprocessing was conducted using Progenesis QL software (Waters), followed by multivariate statistical analysis performed in SIMCA software. Pathway enrichment analysis of the identified metabolites was carried out using the Kyoto Encyclopedia of Genes and Genomes (KEGG) database and the Majorbio Cloud Platform.

### Statistical analysis

All experimental data were analyzed using GraphPad Prism software (version 10.1.2) and R software (version 4.2.0). Results are expressed as mean ± SEM. Statistical significance was defined as *P* < 0.05. For comparisons between two groups, unpaired two-tailed Student’s *t*-test was applied. For comparisons among multiple groups, one-way analysis of variance (ANOVA) followed by Tukey’s post-hoc test was used. Differential abundance of bacterial taxa between groups was analyzed using DESeq2 (version 1.34.0) based on the negative binomial distribution model, as well as LEfSe (Linear Discriminant Analysis Effect Size) with an LDA score threshold > 2. Untargeted metabolomics data were first subjected to Orthogonal Projections to Latent Structures Discriminant Analysis (OPLS-DA) using SIMCA software (version 14.1). Metabolites with Variable Importance in Projection (VIP) > 1.0 and fold change > 1.5 or < 0.67, combined with univariate P < 0.05, were defined as significantly differential metabolites. For correlation analyses between differential microbiota and differential metabolites, Spearman’s rank correlation coefficient was applied due to the non-normal distribution of the microbiome and metabolomics data, as confirmed by the Shapiro–Wilk test.

## Results

### Virtual screening and compounds synthesis

Through the substitution of functional groups in NSC158011, a total of 1440 structurally optimized derivatives were successfully obtained. Subsequently, molecular docking simulations were conducted to analyze the interactions between these derivatives and the protein crystal structure of CpLDH (*C. parvum* lactate dehydrogenase) (PDB code: 4ND5) and H-LDH (human lactate dehydrogenase) (PDB code: 1I0Z). The binding affinities of the compounds for the CpLDH active site were evaluated using scoring functions. Among the derivatives, 722 candidates with high CpLDH binding affinity were identified. These derivatives exhibited binding affinities comparable to or greater than NSC158011 while maintaining low H-LDH binding affinity (S1 Table). In the process of synthesis, we found that there were a large number of sulfur compounds in these compounds. Considering that thioether drugs were relatively few, while sulfone and sulfoxide drugs were more common, we carried out the oxidation reaction of sulfur compounds and obtained more than 20 sulfones and sulfoxides. A total of 29 derivatives of NSC158011 were synthesized (S1A Fig), the spectral characterization results of NSC158011 and two valid derivative substances are shown in S1B Fig.

To further validate the findings, molecular docking simulations and visualizations were performed for NSC158011 and H25/29, an effective derivative selected through subsequent experimental validation, using software tools such as AutoDock Tools and PYMOL. The calculated binding affinity of NSC158011 to the CpLDH complex was -5.55 kcal/mol, involving hydrophobic and hydrophilic interactions with key residues including SER-76, MET-72, and GLN-178, as well as non-polar aromatic rings within the molecule. For H25, the binding affinity to the CpLDH complex was -6.56 kcal/mol, with interactions occurring at residues VAL-166, ARG-173, and SER-170, along with non-polar aromatic rings. Similarly, H29 demonstrated a binding affinity of -5.83 kcal/mol to the CpLDH complex, interacting with residues PH-247 and VAL-298, as well as non-polar aromatic rings (Fig 1A).

In contrast, the binding affinity of NSC158011 to the H-LDH complex was -4.34 kcal/mol, characterized by hydrophobic and hydrophilic interactions with residue ASP-130 and non-polar aromatic rings. H25 exhibited a binding affinity of -4.29 kcal/mol to the H-LDH complex, interacting with residue LEU-330 and non-polar aromatic rings. Lastly, H29 showed a binding affinity of -3.99 kcal/mol to the H-LDH complex, forming interactions with residues LEU-44 and LEU-264, as well as non-polar aromatic rings (Fig 1B).

### Inhibitory effect of NSC158011 and its derivatives on *Cryptosporidium parvum*

The recombinant plasmid pET28A-LDH was constructed through ligation following double digestion of the pET28a vector with BamHI and XhoI restriction enzymes. The double digestion was confirmed by agarose gel electrophoresis, which revealed distinct bands corresponding to the vector fragment and the target insert (S2A Fig). Subsequently, the recombinant plasmid was transformed into *E. coli* BL21 (DE3). Colony PCR analysis demonstrated a clear band of approximately 966 bp, confirming the successful construction of the expression vector (S2B Fig). Induction of the recombinant *E. coli* BL21 (DE3) strain was performed under optimal conditions: 0.8 mM IPTG at 37°C for 2 hours in a shaking incubator (S2C Fig). Following induction, cell lysates were prepared via ultrasonication, and the induced proteins were purified under various purification conditions. Based on experimental results, the optimal purification condition was determined by elution with 500 mM imidazole. After purification, *C. parvum* lactate dehydrogenase (CpLDH) was obtained with high concentration and activity, reaching a concentration of 10,570 ng/μL (S2D Fig).

We conducted enzyme kinetics parameter determination and cytotoxicity studies on 29 synthesized derivatives and found that 14 of them had good in vitro inhibitory activity (Fig 2B). Cytotoxicity assays revealed that 14 compounds (H25, H29, H10, H02, H07, H15, H01, H27, H12, H28, H17, H11, H30, H19) demonstrated relatively low cytotoxicity. Consistent with previous studies, compounds with a selectivity index (SI = CC_50_/IC_50_) greater than 10 were considered to have acceptable selectivity. According to this criterion, H25 (SI = 37.92) and H29 (SI = 16.65) were classified as selective compounds, while the parent compound NSC158011 showed a lower selectivity index (SI = 11.56). These results indicate that structure optimization significantly improved the therapeutic window of the lead compound (Table 1). Preliminary screening was conducted at concentrations at least 50% lower than the cytotoxic IC_50_ values of the respective compounds. It was found that derivatives of H25 and H29 significantly inhibited the proliferation of *C. parvum* 48 hours post-infection (*P* < 0.05). Consequently, NSC158011 and these two derivatives were selected for further analysis. Their anti-cryptosporidial activities were tested at varying concentrations (NSC158011: 0 μM, 0.5 μM, 2.5 μM, 10 μM, 20 μM, 40 μM; H25/H29: 0 μM, 2.5 μM, 10 μM, 20 μM, 40 μM, 80 μM), and their anti-cryptosporidial IC_50_ values derived. The maximum inhibitory concentrations (IC_50_) of NSC158011, H25, and H29 against *C. parvum* were obtained using GraphPad Prism software from dose-response curves. The anti-cryptosporidial IC_50_ values for NSC158011, H25, and H29 were 11.12 μM, 7.44 μM, and 8.42 μM, respectively (Fig 2C). RT-qPCR was performed on the cell/parasite cDNA obtained by reverse transcription, and the ratio of *C. parvum* 18S rRNA to host cell GAPDH was calculated. The Cp-18S rRNA/H-GAPDH value in the untreated group was observably higher than that in the uninfected group (control group), NSC158011 group and H25 and H29 derivative groups, indicating that the derivatives (H25 and H29) reduced the load of *C. parvum* better than the parent compound (Fig 2A).

**Table 1:** Cytotoxicity (CC_50_), anti-*Cryptosporidium* activity (IC_50_), and selectivity indices (SI) of NSC158011 and its derivatives against HCT-8 cells.

Compound	CC_50_(µM)	IC_50_ (µM)	Selectivity Index (SI)²
NSC158011	128.53	11.12	11.56
H25	282.11	7.44	37.92
H29	140.20	8.42	16.65
H10	113.31	ND³	ND
H02	142.47	ND	ND
H07	97.49	ND	ND
H15	281.01	ND	ND
H01	115.67	ND	ND
H27	165.43	ND	ND
H12	77.25	ND	ND
H28	253.06	ND	ND
H17	169.62	ND	ND
H11	77.35	ND	ND
H30	190.22	ND	ND
H19	156.99	ND	ND

IC_50_ values against *C. parvum* were determined from dose–response curves (Fig 2c). SI = CC_50_/ IC_50_. Compounds with SI > 10 are considered to have acceptable selectivity. H25 and H29 showed SI values of 37.92 and 16.65, respectively, indicating favorable selectivity. ³ ND: not determined, as these compounds did not exhibit sufficient anti-*C. parvum* activity in the preliminary screening to generate reliable dose–response curves.

**Fig 2:**
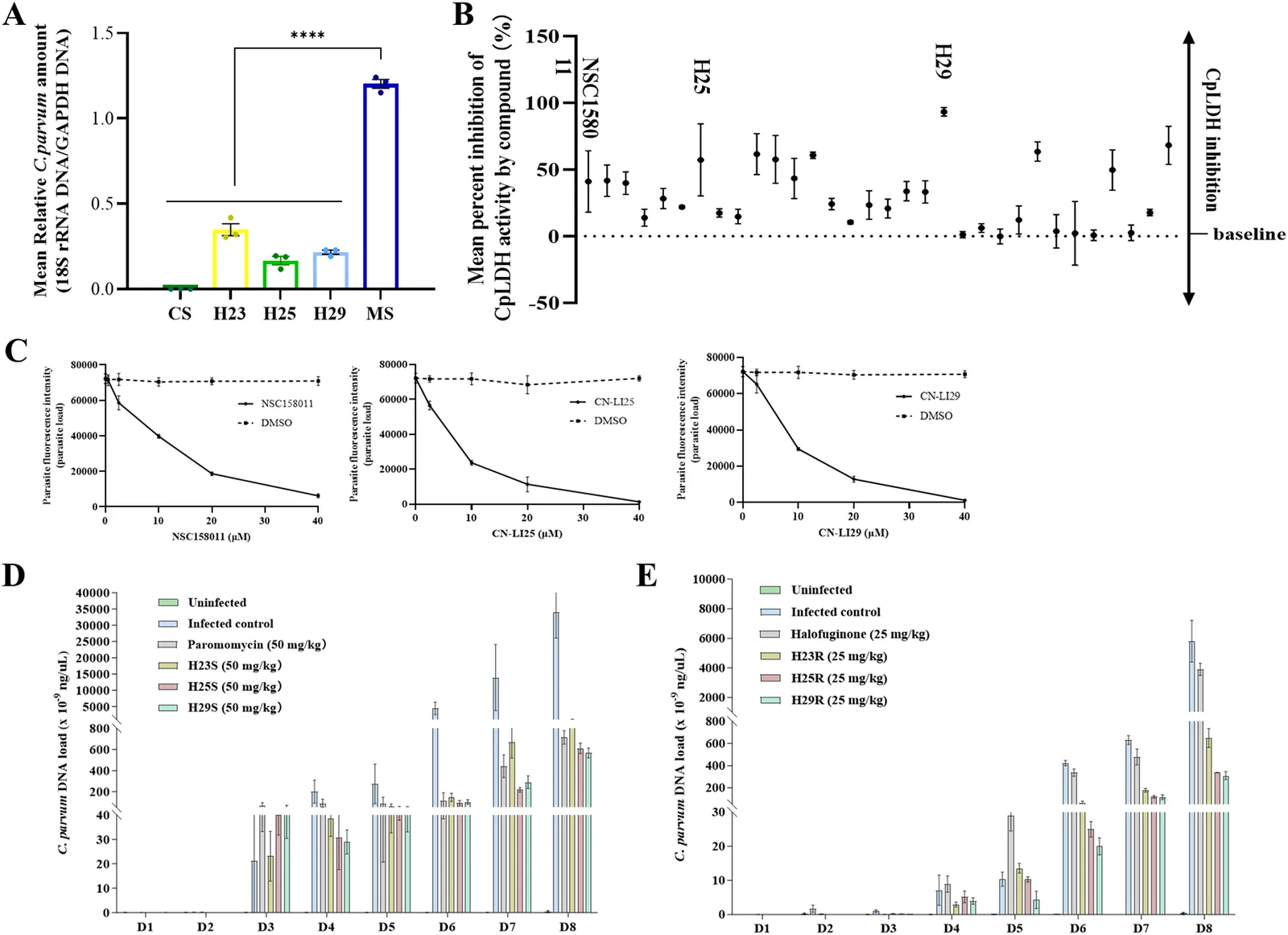
A: RT-qPCR was employed to evaluate the impact of the compound on the quantity of *C. parvum* in HCT-8 cells. B: Investigation into the inhibitory effect of the synthesized compound (2 μM) on the catalytic activity of recombinant CpLDH. C: Immunofluorescence analysis was conducted to assess the effects of varying concentrations of the compound on the growth of *C. parvum* in HCT-8 cells. D: Quantitative results of *C. parvum* oocysts in mouse feces. E: Quantitative results of *C. parvum* oocysts in calf feces. Statistical significance is indicated as **p < 0.01, ***p < 0.001, ****p < 0.0001. Data are presented as mean ± SEM. Abbreviations: RT-qPCR, reverse transcription quantitative polymerase chain reaction; *C. parvum, Cryptosporidium parvum*; HCT-8, human ileocecal adenocarcinoma cell line.

Genomic DNA extraction from individual fecal samples collected from mice and calves on different days post-infection and treatment were performed for *C. parvum* 18S rRNA quantitative analysis. On day 8, the number of oocysts in the feces of mice in the untreated group was about 340 × 10^-7^ng/μL, while the number of oocysts in the feces of mice treated with NSC158011 and its derivatives H25, H29 and paromomycin ranged from 5 × 10^-7^ to 10 × 10^-7^ng/μL. Compared with untreated infected mice, treatment with NSC158011 and its derivatives H25, H29 and paromomycin reduced the number of *C. pravum* oocysts in mouse feces by about 30–60-fold (Fig 2D). On day 8, the number of oocysts in the feces of the control group (MR) calves was about 60 × 10^-7^ng/μL, while the number of oocysts in the feces after treatment with NSC158011 and its derivatives H25 and H29 was 3 × 10^-7^ ~ 6 × 10^-7^ng/μL. Treatment with NSC158011 and its derivatives H25 and H29 reduced the number of *C. puvum* oocysts in calf feces by about 10–20-fold (Fig 2E).

### Restorative effect of NSC158011 and its derivatives on intestinal barrier

Histopathological analysis revealed that uninfected mice preserved the integrity of the intestinal mucosa, whereas lesions in infected mice were characterized by villous atrophy and mucosal erosion. Infected mice treated with NSC158011, H25, and H29 exhibited intact intestinal mucosa and villi, with their structural integrity comparable to that observed in mice treated with paromomycin (Fig 3A). Compared to the treated groups, the untreated group demonstrated a significant reduction in villus height and villus/crypt ratio, while crypt depth remained unchanged (Fig 3B). In the calf model, the infected untreated group (MR) exhibited typical intestinal pathology, including inflammatory cell infiltration, dilation of the central chylomicron duct, and disruption of the intestinal villus structure. Conversely, compound-treated groups maintained better integrity of the intestinal mucosa and villus structure (Fig 4A). To elucidate the specific mechanisms underlying these changes, molecular-level investigations were conducted. RT-qPCR quantification indicated that *C. parvum* colonization in the intestines markedly downregulated the expression of TJ proteins (Occludin, ZO-1, and Claudin-1), thereby compromising intestinal integrity and concurrently increasing the expression level of Cleaved Caspase-1. Following treatment, the H29-treated group showed significant restoration of Occludin and ZO-1 expression, while H25 exhibited the most pronounced upregulation effect on Claudin-1 and effectively inhibited Caspase-1 activation (Figs 3C and 4C). Further verification at the protein level confirmed that the protein expression changes were largely consistent with the gene expression results (Fig 4D). Serological analysis revealed that pro-inflammatory factors (IL-1β, IL-6, and TNF-α) were elevated in infected calves, while the anti-inflammatory factor IL-10 decreased; conversely, these trends were reversed in the compound-treated groups (Fig 4B). Collectively, the experimental findings suggest that NSC158011 and its derivatives cooperatively regulate tight junction protein expression and inhibit the caspase-1-mediated inflammatory signaling pathway, which may contribute to the improvement of intestinal barrier damage and systemic inflammatory responses associated with *C. parvum* infection.

**Fig 3:**
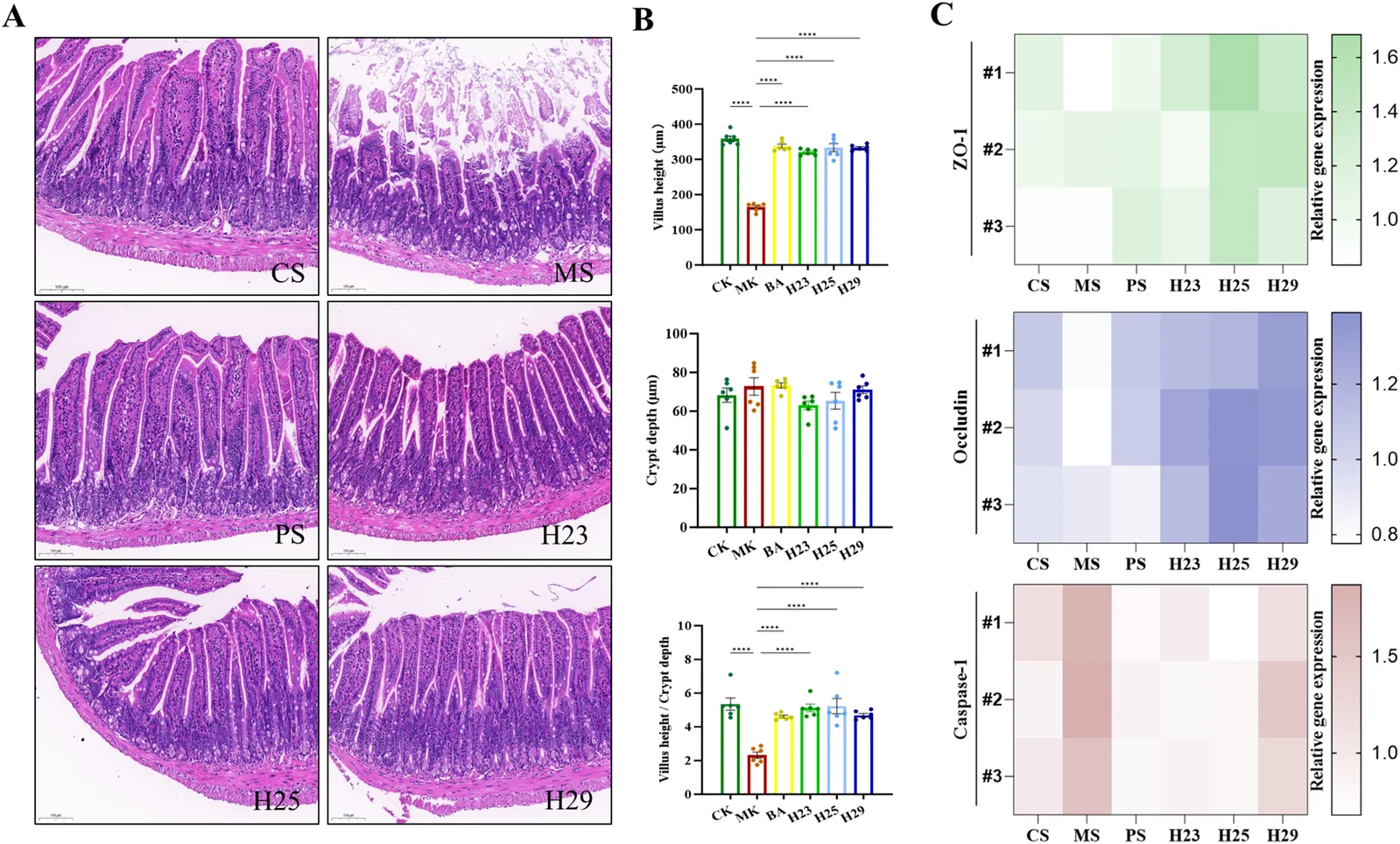
A: HE staining of mice small intestine. B: Analysis of the height of intestinal villi and the depth of intestinal crypts and their ratios among the six groups.C: RT-qPCR was used to analyze the expression levels of barrier and inflammation-related genes in the intestinal tissues of mice. Statistical significance is indicated as *p < 0.05, **p < 0.01, ***p < 0.001, ****p < 0.0001. Data are presented as mean ± SEM. Abbreviations: HE, hematoxylin and eosin; RT-qPCR, reverse transcription quantitative polymerase chain reaction; ZO-1, zonula occludens-1; SEM, standard error of the mean.

**Fig 4:**
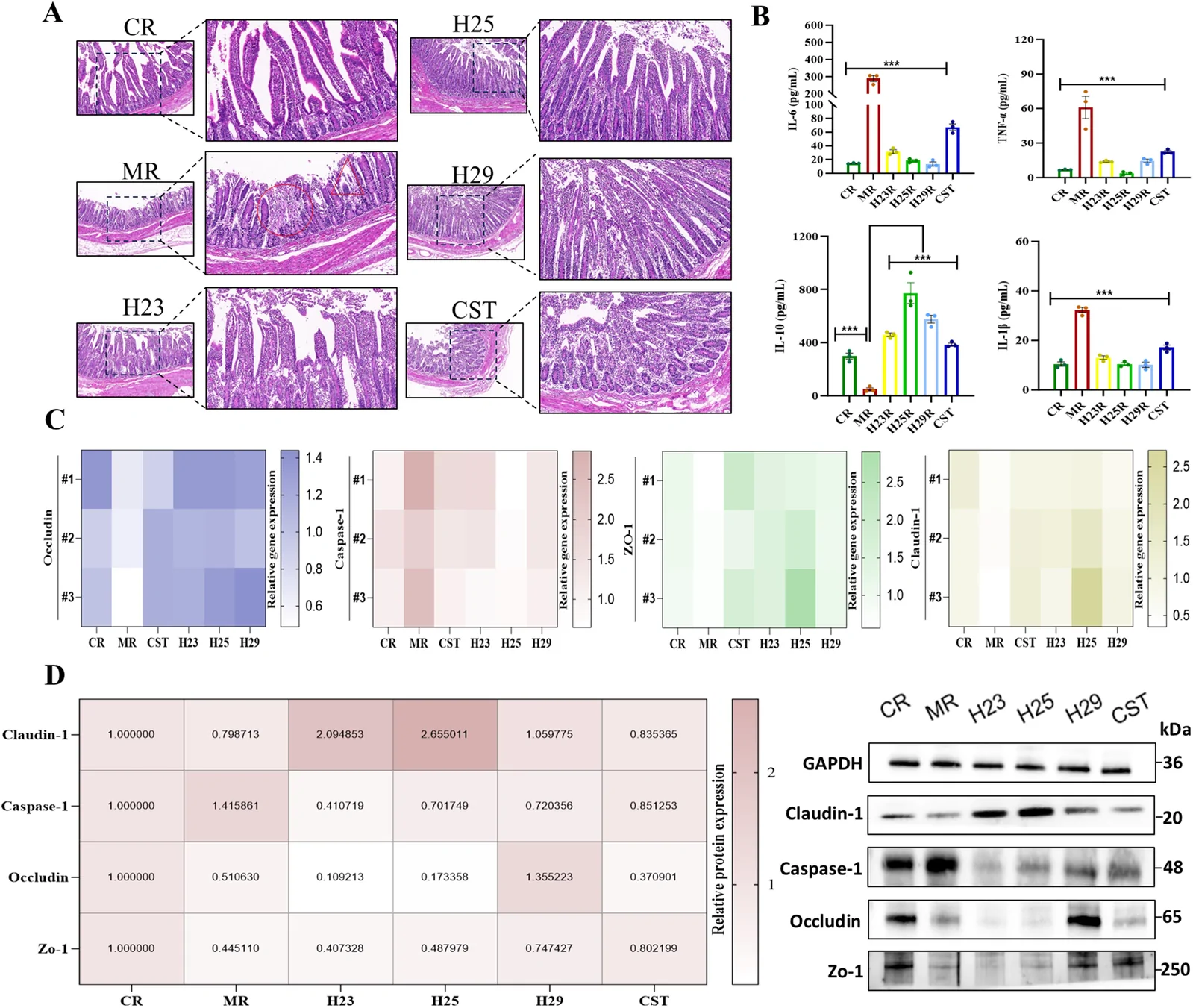
A: HE staining of calf small intestine. B: The content of inflammatory factors in calf serum. C: RT-qPCR was used to analyze the expression levels of barrier and inflammation-related genes in calf intestinal tissues. D: Western Blot was used to analyze the expression levels of barrier and inflammation-related proteins in calf intestinal tissues. Statistical significance is indicated as *p < 0.05, **p < 0.01, ***p < 0.001, ****p < 0.0001. Data are presented as mean ± SEM. Abbreviations: HE, hematoxylin and eosin; RT-qPCR, reverse transcription quantitative polymerase chain reaction; ZO-1, zonula occludens-1; GAPDH, glyceraldehyde-3-phosphate dehydrogenase; IL-6, interleukin-6; IL-10, interleukin-10; IL-1β, interleukin-1 beta; TNF-α, tumor necrosis factor-alpha; SEM, standard error of the mean.

### NSC158011 and its derivatives alleviate intestinal damage by influencing the intestinal microecology

We performed 16S rRNA sequencing on the intestinal contents of mice. The relative abundance of dominant microbial groups was quantified at the phylum and genus levels using microbial classification methods. At the phylum level, a total of 14 phyla were identified in the intestinal microbiota across all samples (CS = 10, MS = 11, PS = 10, H23S=11, H25S=11, H29S=10). As shown in the cumulative relative abundance bar chart for the top 10 species at both the phylum and genus levels (Fig 5A), Bacteroidota (CS = 56.56%, MS = 46.44%, H23S=17.45%, H25S=25.14%, H29S=28.09%), Firmicutes_D (CS = 13.91%, MS = 18.63%, H23S=17.45%, H25S=25.14%, H29S=28.09%), and Firmicutes_A (CS = 25.83%, MS = 24.61%, H23S=25.96%, H25S=29.92%, H29S=17.34%) constituted the three most abundant phyla in the CS, MS, H23S, H25S, and H29S groups, collectively accounting for approximately 96% of the bacterial community. In the PS group, the top three most abundant phyla were Bacteroidota (66.96%), Firmicutes_D (18.11%), and Proteobacteria (8.00%). Notably, Proteobacteria exhibited low proportions in the CS, H23S, and H25S groups (<0.71%) and accounted for approximately 1.82% in the MS group. Other phyla, such as Cyanobacteria (CS = 0.43%, MS = 0.043%, PS = 0.15%, H23S=0.06%, H25S=0.05%, H29S=0.04%) and Desulfobacterota_I (CS = 1.92%, MS = 3.57%, PS = 0.03%, H23S=5.23%, H25S=4.39%, H29S=1.89%), demonstrated relatively low richness. To further elucidate shifts in the composition of intestinal bacteria in mice, an analysis was conducted at the genus level, identifying a total of 173 genera. At this level, *Lactobacillus* (CS = 10.32%, H25S=13.41%, H29S=10.51%) ranked among the top three most abundant genera in the CS, H25S, and H29S groups. The other two dominant genera in the CS group were *Alloprevotella* (8.53%) and *Prevotella* (8.37%). In the H25S group, *UBA3282* (11.03%) and *Limosilactobacillus* (6.35%) were the other two dominant genera, while *Ligilactobacillus* (18.25%) and *Duncaniella* (12.33%) were the predominant genera in the H29S group. The top three most abundant genera in the MS group included *CAG-485* (8.21%), *Lactobacillus* (6.87%), and *Duncaniella* (6.66%). In the PS group, *Phocaeicola_A_858004* (15.19%), *Bacteroides_H* (13.91%), and *CAG-485* (9.20%) accounted for relatively high proportions. Finally, in the H23S group, the top three most abundant genera were *UBA3282* (11.69%), *Duncaniella* (8.13%), and *Ligilactobacillus* (8.04%). At the phylum and genus levels, a differential analysis of the microbiota composition was performed across the six groups, revealing one phylum and fifteen genera with significant differences among the groups. At the phylum level, the abundance of Proteobacteria exhibited relatively substantial changes between the MS group and the derived CpLDH inhibitor group (H23, H25, H29) (Fig 5B). At the genus level, treatment with NSC158011 and its derivative H25/29 resulted in increased abundances of *Ligilactobacillus*, *Paramuribaculum*, *Merdisoma*, *Tidjanibacter*, *Muribaculum*, and *QWKK01* compared to the MS group. Conversely, the abundances of *Bacteroides_H*, *Turicimonas*, *Scatocola*, and *Eubacterium_R* were higher in the MS group than in the treatment groups (Fig 5C).

**Fig 5:**
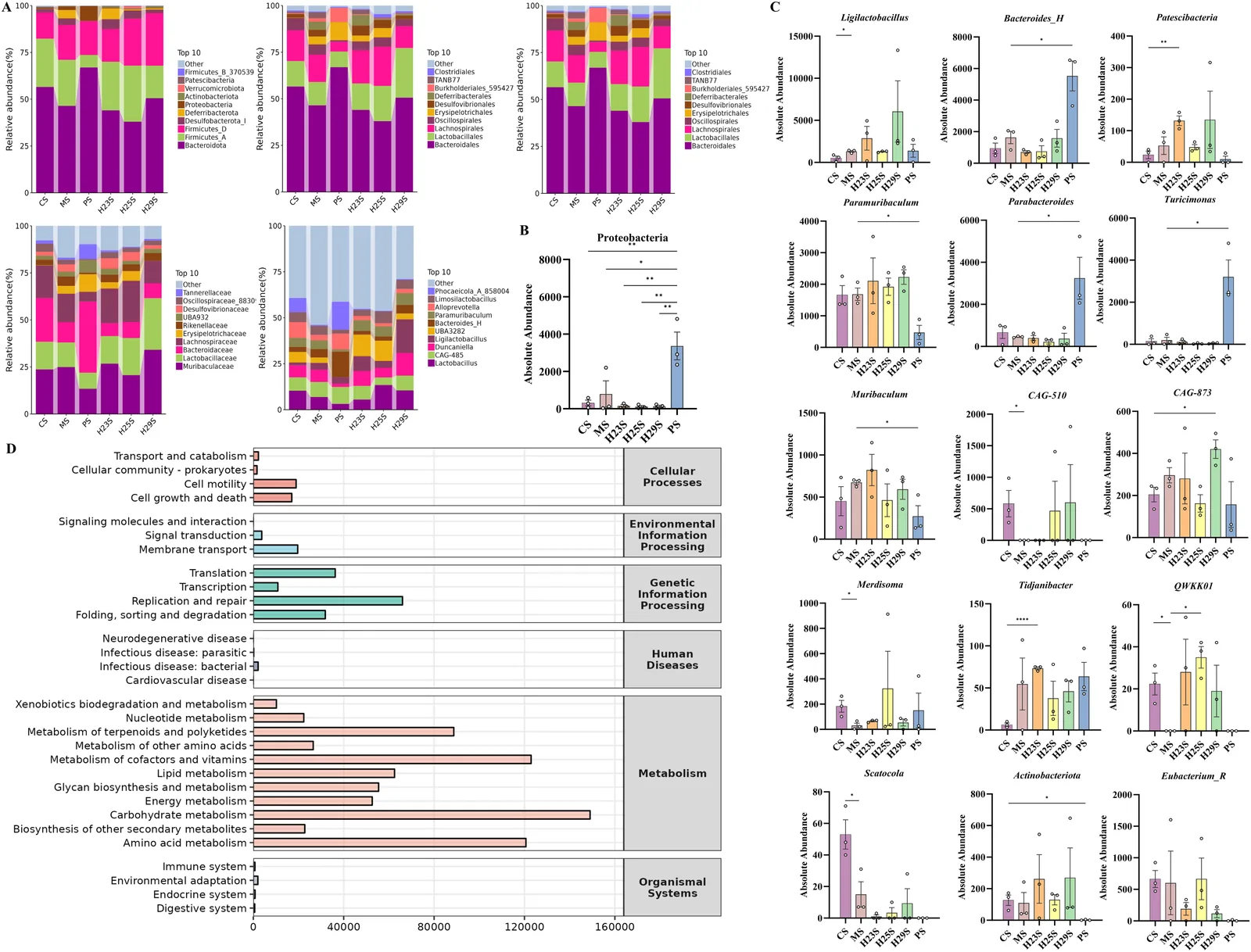
A: A diagram of the accumulation of intestinal flora species at five levels in the mouse intestine. B: Differential microbiota at the phylum level in infected mice and treated mice. C: Differential microbiota at the genus level between infected mice and treated mice. D: Abundance analysis of functional pathways of mouse microbiota. Statistical significance is indicated as *p < 0.05, **p < 0.01, ***p < 0.001, ****p < 0.0001. Data are presented as mean ± SEM.

We observed intriguing alterations in the intestinal microbiota of target animal calves. The relative abundance of dominant microbial groups was determined at both the phylum and genus levels using microbial classification methods. At the phylum level, a total of 55 phyla and 887 genera were identified across all samples within the intestinal bacterial communities. From the cumulative relative abundance bar charts of the top 10 species at the phylum and genus levels, Bacteroidota (MR = 12.19%, H23R=32.91%, H25R=38.53%, H29R=33.25%) and Firmicutes_A (MR = 54.00%, H23R=55.22%, H25R=35.06%, H29R=21.25%) emerged as predominant phyla. Additionally, Firmicutes_D (MR = 31.27%, H23R=4.90%, H25R=21.69%, H29R=21.92%) ranked among the top three phyla in terms of abundance proportion within the MR Group, H23R Group, H25R Group, and H29R Group. In the CR Group, Bacteroidota (37.14%), Firmicutes_D (30.32%), and Actinobacteriota (12.99%) constituted the top three most abundant phyla. Within the CST Group, Firmicutes_A (51.65%) and Bacteroidota (38.87%) exhibited higher abundances. At the genus level, *Bacteroides_H* (CR = 36.45%, H23R=30.44%, H25R=38.54%, H29R=32.55%) demonstrated relatively high proportions in the CR Group, H23R Group, H25R Group, and H29R Group. Specifically, in the CR Group, Enterococcus_A and *Bifidobacterium_388775* accounted for 12.62% and 12.02%, respectively. In the H23R Group, *Porphyromonas_A_859423* had an abundance percentage of 8.10%. In the H25R Group, *Streptococcus* (14.21%) and *Faecalimonas* (13.98%) were relatively abundant. In the H29R Group, *Fusobacterium A* and *Streptococcus* represented 16.44% and 12.03%, respectively. Within the MR Group, *Faecalibacterium* (22.48%) and *Streptococcus* (20.88%) were the dominant genera. Notably, the proportion of *Faecalibacterium* in the other five groups was below 3%, and that of *Streptococcus* was below 15%, indicating relatively low abundances. *Bacteroides_H* constituted only 4.95% in the MR Group. In the CST Group, *Faecousia* (10.65%) was the dominant genus, while other genera such as *Bacteroides_H*, *Parabacteroides_B_862066*, *CAG-485*, *Cryptobacteroides*, and *Phocaeicola_A_858004* exhibited abundances below 10% (Fig 6A). By reanalyzing the abundance of the intestinal microbiota in calves, we identified significant differences in the abundances of 8 phyla (Fig 6C) and 40 genera compared to those in the MR Group. Specifically, the abundance of *Bacteroides* was reduced in the MR Group, leading to an F/B imbalance characterized by a marked decrease in Bacteroides abundance. In contrast, the F/B ratio remained relatively normal in the three derived CpLDH inhibitors treatment groups (Fig 6B). At the genus level, the three derived CpLDH inhibitors (H23R, H25R, and H29R) significantly influenced the abundance of intestinal bacteria. Compared with the MR Group, several genera were highly abundant in the treatment groups, including *Bacteroides_H*, *Faecalimonas*, *Ruminococcus_B*, *Enterococcus_B*, *Limivicinus*, *Limiplasma*, *Pygmaiobacter*, *Exiguobacterium_A*, *Phascolarctobacterium A*, *Enterenecus*, *Clostridium_X*, *JC017*, *Lawsonia*, *Acinetobacter*, *Enterococcus_H_360604*, *Terrisporobacter*, *Campylobacter_B*, *Paludicola*, *Mycobacterium*, *Ligilactobacillus*, *Clostridium_J*, and *RUG420*. Conversely, the abundances of *Faecalibacterium*, *Clostridium_P*, *Phocaeicola_A_858004*, *Akkermansia*, *UMGS1071*, and *Fournierella* were relatively low in the treatment groups but higher in the MR Group (Fig 6D).

**Fig 6:**
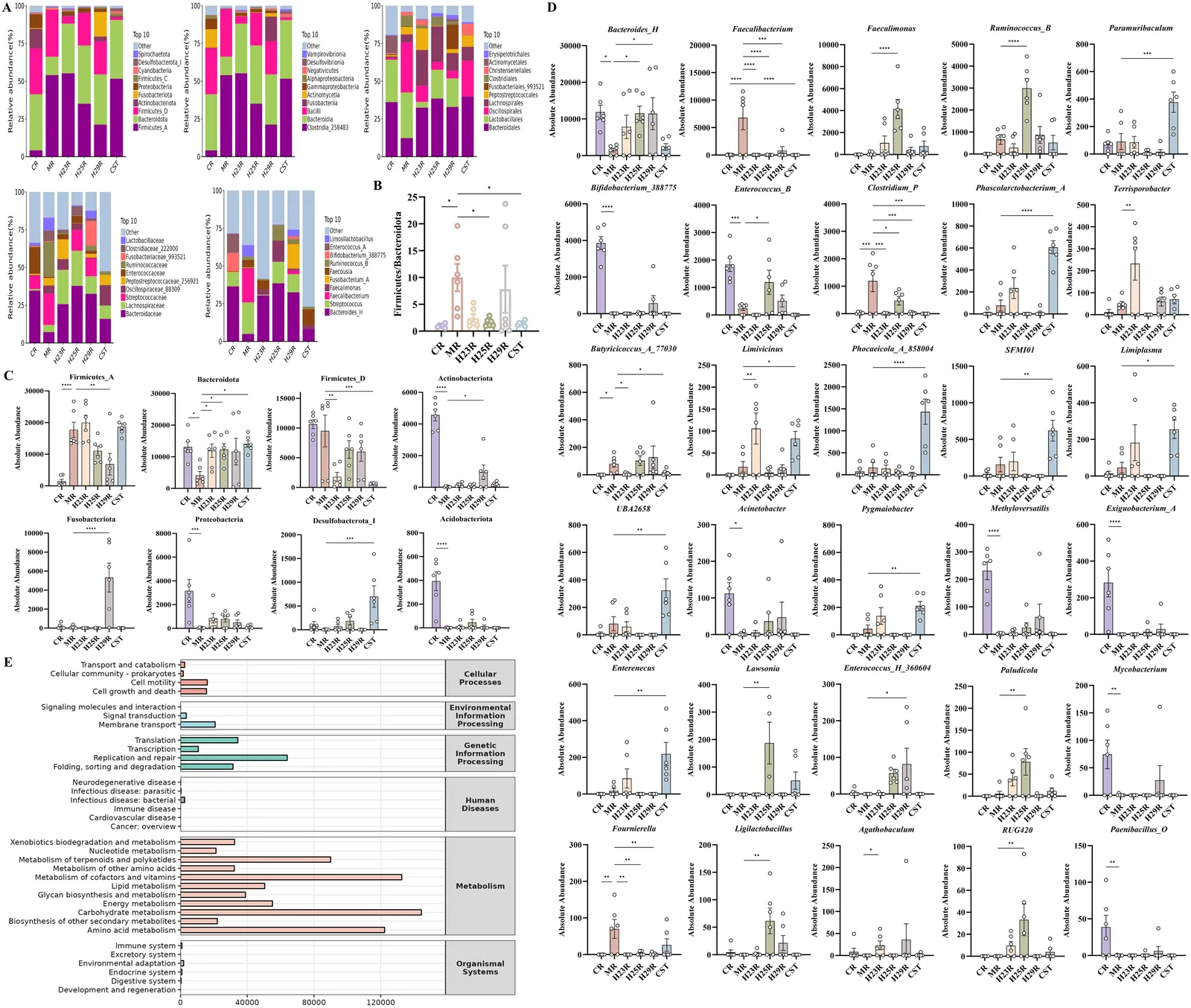
A: A diagram of the accumulation of intestinal flora species at five levels in the calf intestine. B: Firmicutes/Bacteroidetes ratio at the phylum level. C: Differential microbiota at the calf phylum level between infected calves and after treatment. D: Differential microbiota at the genus level in infected calves and calves after treatment. E: Abundance analysis of the functional pathways of calf microbiota. Statistical significance is indicated as *p < 0.05, **p < 0.01, ***p < 0.001, ****p < 0.0001. Data are presented as mean ± SEM.

We analyzed the differences in fungal composition between each group and the MR Group and found that there were significant differences in 9 genera. Compared with the CR group, the abundance of *Fusarium*, *Penicillium* and *Arnium* increased in the MR Group, while their abundance decreased in the CpLDH inhibitor treatment group but was still higher than that of the CR group. The abundances of *Botryotrichum*, *Dactylonectria*, *Oliveonia*, *Geotrichum* and *Rhizoctonia* increased in the CpLDH inhibitor treatment group and decreased in the MR Group (S3 Fig).

### NSC158011 and its derivatives ameliorate intestinal damage by influencing metabolism

To investigate the overall metabolomics changes, we performed orthogonal projection to latent structure discriminant analysis (OPLS-DA). The scatter plot demonstrates a remarkable separation between the infection-only group and the untreated control group (MR Group), as well as among other groups, indicating that *C. parvum* infection alters the metabolomic profiles of calves. Metabolites from each group were compared against those in the MR Group using the merge function for integrated analysis. Differential metabolites were further analyzed using Volcano and Stem plots. Compared with the CR Group, the MR Group exhibited 1627 differentially expressed metabolites (1022 upregulated and 605 downregulated) (S4A Fig). Notably, L-Urobilin, Indolmycin, 1,7-Bis(4-hydroxyphenyl)-3-heptanone, Tolnaftate, Vitamin E, Lipoxin B4, 2-Hydroxy-2-methylbutyric acid, and Firefly luciferin were significantly upregulated. Conversely, Theophylline, Alloxan, Butenoyl-PAF, 6-Aminopenicillanic acid, O-Succinyl-L-Homoserine, 4-Hydroxycinnamic acid, and Methazolamide were significantly downregulated (S5A Fig). Compared with the MR Group, the H23 Group showed 163 differential metabolites (94 upregulated and 69 downregulated) (S4B Fig). Significant upregulation was observed for Alloxan, Baicalin, O-Succinyl-L-Homoserine, Bergapten, Isopropamide, and 3-Methylxanthine, while significant downregulation occurred for 3,4-Dihydroxyhydrocinnamic acid, L-(−)-Sorbose, Leu-Ty, Carnitine C18:2, and 1-Eicosanoyl-sn-glycero-3-phosphocholine (S5B Fig). Furthermore, 147 differential metabolites (105 upregulated and 42 downregulated) were identified between the MR and H25 Groups (S4C Fig). In the H25 Group, pathways such as 1,2-Dioleoyl-sn-glycero-3-phospho-(1’-myo-inositol), Fendiline, Isopropamide, Vilazodone, Geranyllinalool, 8-Epi-prostaglandin F2alpha, and Licoricidin were significantly upregulated. Conversely, pathways including Dienestrol, 2-Methoxyestradiol, 3,4-Dihydroxyhydrocinnamic acid, Tolnaftate, Perhexiline, and Hecogenin were significantly downregulated (S5C Fig). In the H29 Group, a total of 171 differential metabolic pathways were detected compared with the MR Group (131 upregulated and 42 downregulated) (S4D Fig). Significant upregulation was observed for Theophylline, Butenoyl-PAF, Methazolamide, Glycerophosphoric acid, 1,2-Dioleoyl-sn-glycero-3-phospho-(1’-myo-inositol), 6-Aminopenicillanic acid, Phenols, and 3-Methylxanthine. Significant downregulation was noted for 3,4-Dihydroxyhydrocinnamic acid, Dienestrol, L-(−)-Sorbose, Rosmarinic acid, Glu-Arg, and Hydroxyisocaproic acid. Pathway enrichment analysis revealed that *C. parvum* infection altered multiple metabolic pathways of the intestinal microbiota in calves (S5D Fig). Interestingly, derived CpLDH inhibitors had a significant impact on these altered metabolic pathways.

We performed an in-depth analysis of the metabolite abundances across each sample group and identified 39 representative metabolites with significant differences. The results indicate that, compared to the control group, in the model group, the levels of L-Homophenylalanine, 1-Methylhistamine, Phe-ala, DL-2-Aminooctanoic acid, n-Oleoylethanolamine, Tetraethylene glycol, 5’-deoxy-5’-(Methylthio)adenosine, (R)-Sulcatol, Cytidine, Ornithine, Alanylphenylalanine, 4-Pentenoic acid, N-Acetyl-D-glucosamine, Hippuric acid, Isocitric acid, and Xanthine were significantly reduced. Conversely, these metabolites exhibited an upward trend in the treatment group. In contrast, in the model group, Leu-Val, Tyr-Ile, Dihydroactinidiolide, N-Methylalanine, Tyramine, 5-Hydroxytryptophol, Diethyl glutarate, Elemonic acid, 5-Methoxyindoleacetate, Mercaptopurine, Leu-Thr, Gln-Gln, N-Acetylthreonine, L-Norleucine, Glycolithocholic acid, and Orotic acid showed signally higher abundance than in the control and treatment groups (Fig 7).

**Fig 7:**
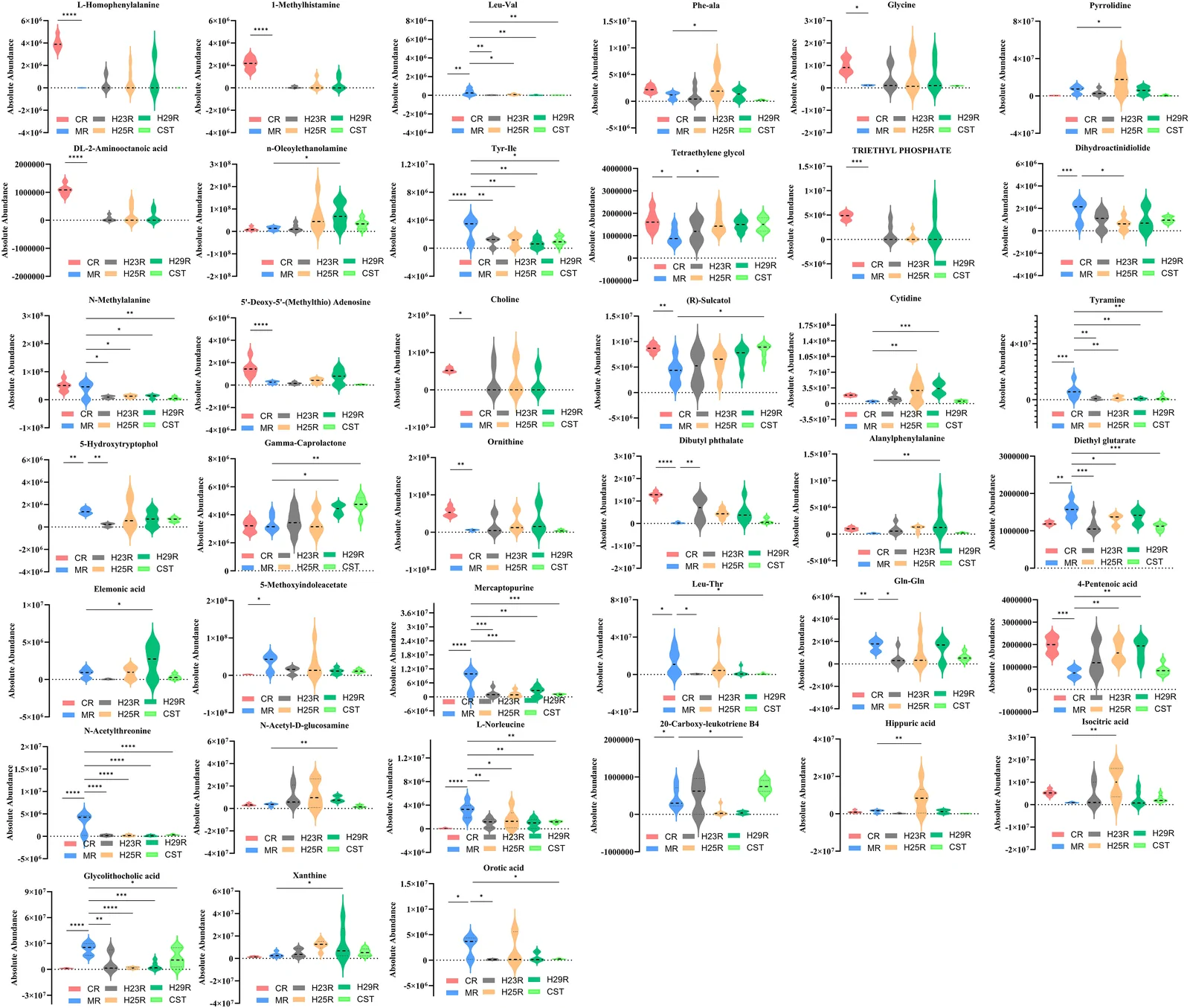
Differential metabolites of the intestinal microbiota in infected calves and treated calves.

### Association analysis of NSC158011 and its derivatives on differential microbiota and metabolites

We analyzed the associations between differential metabolites and differential microorganisms among each group and the MR group using heatmaps. In the comparison between the CR and MR groups, a total of 27 differential microbiota and 20 differential metabolites were selected for association analysis. The results demonstrated that the abundance of *Bacteroides_H* in the MR group was obviously lower than in other groups and was negatively correlated with Vitamin E, Lipoxin B4, Oxoamide, and L-Urobilin. Additionally, Choline, 8-Geranylumbelliferone, 1-Methylnicotinamide, 1-O-Palmitoyl-2-O-acetyl-sn-glycero-3-phosphorylcholine, L-Isoleucine, Malic acid, and Acetyl-L-carnitine were positively correlated. Meanwhile, *Bifidobacterium*_388775 was positively correlated with 7 alpha-hydroxycholesterol, Choline, Alanylarginine, 1-Methylnicotinamide, FFA (16:0), and 4-Phenyl-2-butanol, while it was negatively correlated with 1,7-Bis(4-hydroxyphenyl)-3-heptanone, Lipoxin B4, and Glyceraldehyde (S5F Fig). In the comparison between the MR group and the H23 group, through the analysis of 37 differential microbiota and 20 differential metabolites, it was found that *Bacteroides_H* exhibited a strong positive correlation with LPC (0:0/20:4) but a negative correlation with Chenodeoxycholic acid. *Limosilactobacillus* was positively associated with LPC (0:0/18:2), Piperidine, Soyasaponin I, 1-Heptadecanoyl-sn-glycero-3-phosphocholine, and Xylose, while it was negatively correlated with Succinic acid semialdehyde, Phenol, and Phosphocholine (S5G Fig). Further analysis of the data from the MR group and the H25 group revealed that *Bacteroides_H* and metabolites such as 1-(9Z-octadecenoyl)-sn-glycero-3-phosphocholine, Pantothenic acid, n-Oleoylethanolamine, and Glycerophospho-N-Palmitoyl Ethanolamine were positively correlated, whereas they were negatively correlated with Chenodeoxycholic acid. *Faecalibacterium* was positively correlated with Carnitine C14:2 and LPC (0:0/18:2), while it was negatively correlated with Succinic acid semialdehyde, L-Allothreonine, 1-Heptadecanoyl-sn-glycero-3-phosphoethanolamine amide, and Palmitic acid (S5H Fig). Finally, in the comparison between the MR group and the H29 group, it was observed that *Bacteroides_H* showed a significant positive correlation with n-Oleoylethanolamine. *Faecalibacterium* was positively correlated with alpha-hydroxyisobutyric acid, *Hydroxyisocaproic* acid, and 17-Octadecynoic acid, while it was negatively correlated with PC(O-16:0/O-2:0) and Palmitic amide (S5I Fig).

## Discussion

Lactate dehydrogenase (LDH), a key enzyme in the glycolytic pathway, plays a critical role in the energy metabolism of *C. parvum*. The reaction it catalyzes, the reduction of pyruvate to lactic acid, is essential for maintaining the energy supply of parasites. However, current research on CpLDH remains insufficient. By constructing the expression vector for *C. parvum* lactate dehydrogenase (CpLDH), we successfully achieved recombinant expression and purification of this enzyme, thereby establishing a foundation for subsequent studies on enzymatic characteristics and inhibitor screening. Through optimization of expression conditions, we obtained highly pure recombinant CpLDH protein and further evaluated its catalytic activity. Enzyme kinetics analysis confirmed that the recombinant CpLDH exhibited typical LDH catalytic properties. We investigated the impact of NSC158011 derivatives on the catalytic activity of recombinant CpLDH. Experimental results demonstrated that certain derivatives could significantly inhibit the enzymatic activity of CpLDH, with eight compounds exhibiting strong inhibitory effects. Furthermore, we assessed the cytotoxicity of highly effective CpLDH inhibitor derivatives on host cells (HCT-8). Results indicated that while some derivatives exhibited cytotoxicity at higher concentrations, their half maximal inhibitory concentration (IC_50_) was significantly higher for host cells than for CpLDH, enabling effective inhibition of parasite enzyme activity while minimizing damage to host cells. The inhibitory effects of highly effective CpLDH inhibitors on *C. parvum* were verified through immunofluorescence analysis and quantitative determination of *C. parvum* in cells by RT-qPCR. Results revealed that the two derivatives, which were identified as CpLDH inhibitors through enzymatic screening, could markedly reduce the proliferative capacity of parasites in a concentration-dependent manner.

The gastrointestinal tract is one of the earliest developing tissues and organs in the human body. It serves as the largest bacterial reservoir, the largest immune organ, and the primary interface for daily communication with the external environment. This organ plays crucial physiological roles, including digestion and absorption, barrier function, endocrine regulation, and immune response. It is densely populated with endocrine cells, autonomic nerves, microvessels, and lymphatic vessels. The intestinal barrier acts as a critical component that prevents the translocation of bacteria, toxins, and other harmful substances into the bloodstream and other tissues and organs, thereby maintaining intestinal immune homeostasis [31]. A breach in this barrier can lead to bacterial translocation and endotoxin dissemination, resulting in various infections and exaggerated immune responses within the body [32]. Based on their distinct roles, the intestinal barrier can be categorized into biological, chemical, mechanical, and immune barriers [33]. The mechanical barrier effectively prevents the entry of bacteria, viruses, and endotoxins, primarily consisting of epithelial cells and various tight junctions between cells [34]. TJs are predominantly composed of tight junction proteins, such as Occludin, the Claudin and occludens (ZO) family, and Junctional Adhesion Molecules (JAMs) [35]. *C. parvum* infection can impair the host’s intestinal epithelial barrier function, leading to diarrhea and even systemic inflammatory responses. Observations from intestinal pathological sections revealed that the treatment group significantly improved the structural integrity of intestinal villi compared to the infection control group, with crypt hyperplasia and mucosal inflammatory infiltration being markedly alleviated. This histological improvement was consistent with the reduced excretion data observed in the treatment group during quantitative fecal oocyst analysis, suggesting that the compound alleviates intestinal injury by directly killing or inhibiting parasite proliferation. Serum results demonstrated a noteworthy increase in pro-inflammatory cytokines such as IL-6, IL-1β, and TNF-α in the MR Group, while IL-10 levels decreased. This indicated that an inflammatory response occurred in the infected animals, corroborating findings from previous studies on infected dairy cows. Further protein-level verification via Western blot analysis revealed that compared to the control group (CR), the protein expressions of Occludin, ZO-1, and Claudin-1 were significantly reduced in the untreated infection group (MR), whereas Cleaved Caspase-1 expression increased. Tight Junctions (TJs) are critical structures for maintaining the integrity of the intestinal epithelial barrier, with their function depending on the synergistic interaction of ZO-1 (scaffold protein), Claudin-1 (transmembrane protein), and Occludin (transmembrane protein) [35]. The reduction in these proteins may directly increase intestinal epithelial permeability, facilitating the transmembrane transport of pathogens or toxins and exacerbating infection-related pathological damage [36]. *C. parvum* may disrupt the TJ complex through multiple mechanisms. Upon attachment or invasion of epithelial cells, the parasite may secrete proteases (cysteine proteases) that directly degrade TJ proteins [37], leading to the deficiency and loss of small intestinal villi. Additionally, parasitic infections may indirectly affect the localization and stability of TJ proteins by regulating actin cytoskeleton dynamics, ultimately resulting in intestinal barrier dysfunction [38]. Following treatment, Occludin and ZO-1 levels were significantly increased in the H29 group, while Claudin-1 expression was significantly elevated in the NSC158011 and H25 groups, particularly in the H25 group. Cleaved Caspase-1 expression increased in the MR Group, indicating inflammasome pathway activation, which might be closely associated with intestinal barrier disruption caused by *C. parvum* infection. Disruption of intestinal barrier function promotes the translocation of pathogen-associated molecular patterns (PAMPs) [39], activating pattern recognition receptors (NLRP3) and inducing inflammasome assembly and Caspase-1 activation. Conversely, activated Caspase-1 promotes the maturation and release of factors such as IL-1β, amplifying the inflammatory response and disrupting tight junction structures [40]. Serum results revealed that the treatment group observably downregulated the gene expression of pro-inflammatory factors such as TNF-α and IL-1β. After recombinant CpLDH inhibitor treatment, activated Caspase-1 levels were significantly reduced in the intestines of calves in the NSC158011 and its derivative H25/29 groups. The drug effectively reduced parasite load by inhibiting key metabolic enzymes of *C. parvum* (LDH), decreasing PAMP release, weakening inflammasome activation, restoring tight junction protein expression (ZO-1, Occludin), and enhancing intestinal epithelial cell renewal and repair capabilities. In summary, we speculate that *C. parvum* causes disease through a dual mechanism: first, it directly degrades key junction proteins such as ZO-1, Claudin-1, and Occludin by secreting effector proteins or metabolites, damaging the intestinal physical barrier; second, it activates the NLRP3 inflammasome/Caspase-1 signaling pathway, promoting excessive release of pro-inflammatory factors such as IL-1β, IL-6, and TNF-α, triggering an excessive inflammatory response. The compounds likely exert their primary effect by inhibiting CpLDH, a key metabolic enzyme of *C. parvum*, thereby reducing parasite proliferation and the associated epithelial damage. The subsequent reduction in parasite burden may, in turn, contribute to the restoration of tight junction protein expression and attenuation of the Caspase-1-mediated inflammatory pathway. However, we acknowledge that the current data do not allow us to distinguish between direct target-mediated effects and indirect effects resulting from reduced parasite load. Further studies using genetic approaches (e.g., CpLDH conditional knockdown) or direct target engagement assays (e.g., CETSA) are needed to establish the causal relationships.

The intestinal biological barrier is primarily constituted of the intestinal microbiota, which establishes a complex symbiotic relationship with the host to jointly maintain intestinal homeostasis [41]. A vast number of microorganisms reside in the mammalian intestines, reaching up to 100 trillion in quantity [42]. In the healthy human intestinal tract, Bacteroidetes and Firmicutes constitute the largest proportion, accounting for approximately 90% [43]. The ratio of Firmicutes to Bacteroidetes (F/B) is typically maintained within a relatively stable range to ensure the balance and health of the intestinal microbiota environment. Disruption of this equilibrium may lead the microbiota, originally functioning as a defense barrier, to instead cause damage or inflammation to the host organism [44]. In the MR Group, the abundance of Bacteroides decreased, suggesting potential disorders in intestinal function. As members of the Polysaccharide Degradation Consortium, Bacteroidetes possess diverse glycans and glycosidases that enable the utilization of polysaccharides [45]. They assist in breaking down dietary fiber and starch to release energy and may serve as the primary source of propionate [46]. Given that the human digestive system encodes only a limited number of carbohydrate-active enzymes, glycans are not easily digested in the stomach and small intestine [47]. However, the intestinal microbiome can encode tens of thousands of carbohydrate-active enzymes [48]. Bacteria capable of degrading glycans, such as Bacteroides, are referred to as primary degrading bacteria. Primary degradation of glycans releases glucose, while short-chain fatty acids (SCFAs), lactate, and succinate are produced through the action of secondary degrading bacteria. The intestinal microbiota in the MR Group exhibited an imbalance in F/B characterized by a significant reduction in Bacteroides abundance. Conversely, the F/B ratio in the three recombinant CpLDH inhibitor treatment groups remained relatively normal. This suggests that *C. parvum* may competitively consume host energy substrates due to inhibited energy metabolism, leading to a marked decrease in Bacteroides abundance and interference with the host’s normal energy metabolic process. The reduction in Bacteroidetes abundance may also result in decreased generation of SCFAs (specifically propionic acid and butyric acid) [49], further reducing the expression of epithelial tight junction proteins and damaging the intestinal barrier, thereby causing intestinal leakage.

The intestinal microbiota acts a critical role in the host’s physiology, digestion and absorption, immune regulation, and maintenance of intestinal epithelial cell integrity. Our prior research demonstrated that the intestinal microbiota of horses, yaks, Tibetan pigs, experimental mice, and dairy cows naturally infected with *C. parvum* exhibited disorder, characterized by increased pathogenic bacterial abundance and reduced beneficial bacterial abundance [50–52]. In this study, at the genus level, the mouse differentiated microbiota included *Ligilactobacillus*, *Paramuribaculum*, *Merdisoma*, and *Tidjanibacter*. After treatment with NSC158011 and its derivative H25/29, the abundances of Muribaculum and QWKK01 were higher than those in the MS group. Changes in the calf microbiota revealed that, compared with the model group (MR Group), each treatment group showed significant increases in *Bacteroides_*H, *Faecalimonas*, *Ruminococcus_*B, *Enterococcus_*B*, Limivicinus*, *Limiplasma*, *Pygmaiobacter*, *Exiguobacterium*_A, *Phas Colarctobacter* A, *Enterenecus*, *Clostridium* X, JC017, *Lawsonia*, *Acinetobacter*, *Enterococcus*_H_360604, *Terrisporobacter*, *Campyl obacter*_B, *Paludicola*, *Mycobacterium*, *Ligilactobacillus*, *Clostridium*_J, and RUG420. Conversely, *Faecalibacterium*, *Clostridium*_P, *Phocaeicola*_A_858004, *Akkermansia*, UMGS1071, and *Fournierella* exhibited relatively high levels in the MR Group. Notably, the abundance of *Ligilactobacillus* increased in both animals within the recombinant CpLDH inhibitor treatment group. *Ligilactobacillus* is a common microbiota found in the intestines of humans, livestock, poultry, and aquatic animals, playing a vital role in the host’s nutritional metabolism, physiology, and immune processes through competition with harmful microorganisms or expression of beneficial substances [53]. *Paramuribaculum*, a butyrate-producing bacterium and its symbiotic bacteria [54], exhibited increased abundance in the intestinal tract of mice within the recombinant CpLDH inhibitor treatment group. Butyrate plays a pivotal role in physiological processes, including inflammation inhibition and maintenance of normal intestinal function [55]. *Muribaculum* contributes to intestinal health through the production of short-chain fatty acids [56]. Our findings indicate that bacteria with higher abundance in the intestines of calves in the treatment group also demonstrated positive probiotic effects. *Faecalimonas* produces acetate, which helps stabilize the intestinal environment and nourish other beneficial bacteria in the colon [57]. *Pygmaiobacter* is linked to the recovery from non-alcoholic steatohepatitis in rats, and was believed to be related to SCFAs [58]. *Ruminococcus* B, one of the earliest discovered stomach bacteria, plays an essential role in metabolism by breaking down cellulose in the host’s digestive system for nutrient acquisition and fermenting glucose and xylose [59]. Additionally, it stabilizes the intestinal barrier and alleviates diarrhea. Through systematic observation of changes in the microbiota, we identified several significant trends. The gut microbiota acts a pivotal role in host energy metabolism, and *C. parvum* infection can disrupt its function by altering the intestinal microenvironment, thus affecting the host’s energy homeostasis. By comparing the model group (*Cryptosporidium*-infected) with the treatment group (LDH inhibition intervention), we found that metabolic activities associated with *C. parvum* significantly suppressed host energy-related microbiota. In contrast, the abundance of glycogenolytic bacteria and SCFAs-producing bacteria was elevated in the LDH inhibition treatment group. This finding may reflect the metabolic competitive relationship between *C. parvum* and the intestinal microbiota. In the model group, the abundance of bacteria related to energy metabolism, such as Bacteroides, was markedly reduced, potentially linked to the energy hijacking mechanism of *C. parvum,* this is consistent with the sequencing results of the metabolic pathways of the microbiota, with the shifts in the energy pathways being the most obvious (Figs 5D and 6E). As a strictly intracellular parasitic protozoan, *C. parvum* heavily depends on intermediate glycolytic products from the host for anaerobic metabolism, and its lactate dehydrogenase (LDH) activity is critical for lactic acid production. The accumulation of excessive lactic acid leads to localized intestinal acidification, inhibiting the growth of symbiotic bacteria, including butyrate-producing species. Furthermore, *C. parvum* ‘s competitive consumption of carbon sources like glucose reduces available substrates for the microbiota, resulting in metabolic deprivation. These findings align with research on pathogens such as *Giardia lamblia* and *Clostridium difficile*, which inhibit probiotic colonization by altering the intestinal metabolic environment [60]. In the treatment group, the abundance of glycogenolytic bacteria, such as *Bacteroides* and *Ruminococcus* B, as well as SCFA-producing bacteria, such as *Faecalibacterium* and *Roseburia*, significantly increased. This suggests that LDH inhibition may alleviate intestinal acidification by reducing *Cryptosporidium*-derived lactic acid production, thereby promoting the recovery of pH-sensitive SCFA-producing bacteria, such as *Clostridium butyricum*. Additionally, restricted glycolysis in *C. parvum* makes more glucose and glycogen available for symbiotic bacteria, promoting the proliferation of glycogen-degrading bacteria. Notably, SCFAs, particularly butyric acid and propionic acid, serve not only as the primary energy source for colonic epithelial cells but also regulate host glycolipid metabolism via activation of GPR41/GPR43 receptors and reduce inflammatory responses by inhibiting the NF-κB pathway [61,62], thus enhancing intestinal barrier function and reducing *C. parvum* invasion. Moreover, this process improves the overall energy metabolism efficiency of the host.

The metabolites generated by the microbiota can interact with receptors on host cells, thereby activating or inhibiting signaling pathways and exerting either beneficial or detrimental effects on host health [63]. Consequently, we administrated a further exploring of changes in microbiota-derived metabolites. Isocitric acid serves as a key intermediate in the tricarboxylic acid (TCA) cycle, and its reduction indicates mitochondrial dysfunction [64]. Ornithine functions is a bridge molecule between the urea cycle and the TCA cycle [65], while xanthine is an essential intermediate in purine energy metabolism. Our findings revealed that the levels of isocitric acid and ornithine were signally reduced in the model group, accompanied by a decrease in xanthine. This phenomenon suggests a marked inhibition of the mitochondrial energy metabolism pathway, which aligns with previous research on intracellular parasitic infections and may reflect the competitive consumption of host energy by pathogens [66]. Additionally, the content of oleoylethanolamide, which participates in fatty acid oxidation [67], decreased, whereas the abnormal metabolite diethyl glutarate increased, indicating significant inhibition of lipid metabolism pathways. Based on these alterations in energy-related metabolites, we speculated that the TCA cycle in the microbiota of the model group was disrupted, leading to impaired mitochondrial energy production, causing the animals to show weight loss and diarrhea (S4F Fig). In contrast, the treatment group exhibited varying degrees of recovery. Metabolites associated with the gut microbiota display distinct alteration patterns. The pronounced reduction in hippuric acid in the model group was strongly correlated with the diminished abundance of Prevotella, as demonstrated by 16S rRNA sequencing, confirming the microbiota-dependent nature of aromatic amino acid metabolism. The decline in the short-chain fatty acid derivative 4-pentenoic acid in the model group corresponded to the reduced abundance of SCFAs-generating bacteria observed in the microbiota results. Therefore, we hypothesize that the disruption of the TCA cycle and mitochondrial energy production in the MR group leads to insufficient energy supply, directly impacting the synthesis and assembly of TJ proteins in epithelial cells. Disruption of energy metabolism results in the accumulation of mitochondrial reactive oxygen species (ROS) [68], which activates the NLRP3 inflammasome, manifested by upregulated Caspase-1 expression [69]. Activated Caspase-1 promotes the maturation and release of IL-1β, further compromising the integrity of tight junction structures. Furthermore, secondary bile acid glycochenodeoxycholic acid was overexpressed in the model group, potentially over-activating the FXR receptor and contributing to systemic metabolic disorders (Fig 8).

**Fig 8:**
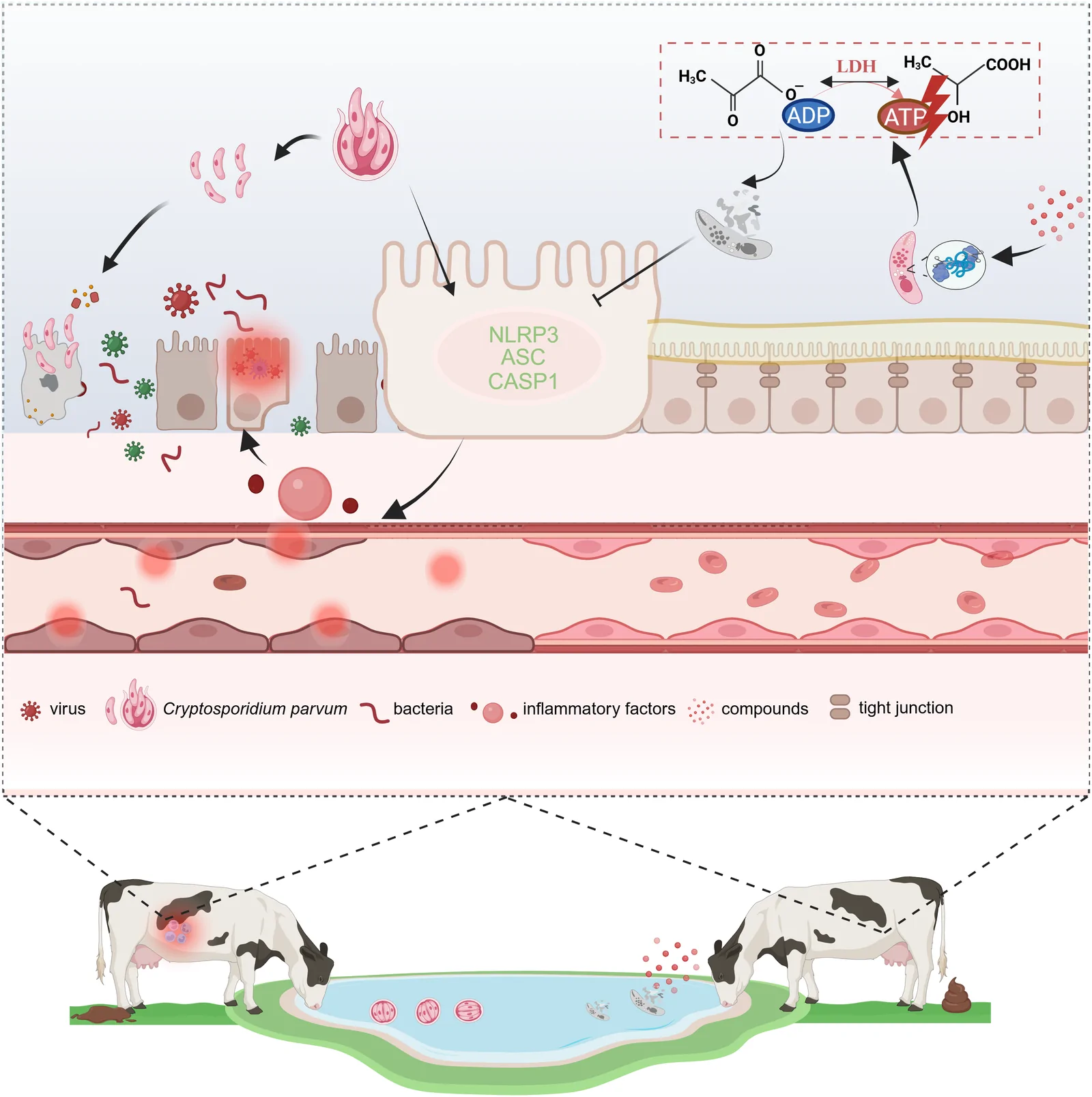
Summary diagram of the mechanism of action. Abbreviations: CpLDH, Cryptosporidium parvum lactate dehydrogenase; TCA, tricarboxylic acid; ROS, reactive oxygen species; TJ, tight junction; ZO-1, zonula occludens-1; GSDMD, gasdermin D; IL-1β, interleukin-1 beta; IL-18, interleukin-18. Created in BioRender. Kun, **L.** (2026) https://BioRender.com/vs16jmb.

We acknowledge several limitations in the current study. First, while we have demonstrated that H25 and H29 inhibit recombinant CpLDH activity in single-concentration screening and exhibit potent anti-*C. parvum* efficacy in cellular and animal models, we have not yet determined the precise IC_50_ values of these compounds against recombinant CpLDH or conducted full enzyme kinetic analyses to define the mode of inhibition. These experiments require large-scale protein purification and systematic kinetic studies, and will be completed in our follow-up investigations. Second, direct target engagement assays (e.g., CETSA or surface plasmon resonance) would provide additional evidence for CpLDH as the direct molecular target, which we also plan to pursue in future studies. Additionally, while our molecular docking results suggest that H25 and H29 bind more weakly to human LDH than to CpLDH (Fig 1b), and cellular cytotoxicity assays demonstrated favorable selectivity indices (SI > 10, Table 1), we acknowledge that these observations do not replace direct biochemical assessment of compound activity against recombinant human LDH isoforms. Quantitative enzyme inhibition assays using human LDH are required to definitively establish species selectivity. These experiments will be performed in our future studies. Besides, comprehensive characterization was performed only for the three lead compounds; the remaining 26 compounds, which were used for preliminary screening, were not subjected to full analytical characterization. Despite these limitations, the consistent results from enzymatic screening, cellular efficacy, animal models, and metabolomics analyses collectively support CpLDH as a relevant target for the anti-*C. parvum* activity of H25 and H29, although we acknowledge that the data presented here represent supportive evidence for target association rather than definitive target validation.

## Supporting information

S1 Table Screening results of molecular docking of derivatives. (DOCX)

S2 Table Mouse Intestinal Barrier and Inflammatory Gene Primers. (DOCX)

S3 Table Calf Intestinal Barrier and Inflammatory Gene Primers. (DOCX)

S1 Fig A: The structural formulas of 30 compounds, 23 is NSC158011, 25 is H25, and 29 is H29. B: Verification of the spectral structure of NSC158011 and its effective derivative substances.
(TIF)

S2 Fig A: Double-enzyme digestion agarose gel electrophoresis. B: Agarose gel electrophoresis of LDH expression vector. Note: M is a marker, and 2 and 3 in Fig A are the carrier digestion bands. 5 and 6 are plasmid digestion bands.
(TIF)

S3 Fig Differential flora of intestinal Fungi in calves. Statistical significance is indicated as *p < 0.05, **p < 0.01, ***p < 0.001, ****p < 0.0001. Data are presented as mean ± SEM.
(TIF)

S4 Fig Volcanic map of intestinal fungal metabolites in calves (A: MR And CR; B: MR And H23; C: MR And H25;D: MR And H29; E: MR And CST). F: Result of calf weight change (n = 3). Statistical significance is indicated as *p < 0.05, **p < 0.01, ***p < 0.001, ****p < 0.0001. Data are presented as mean ± SEM.
(TIF)

S5 Fig Differences in metabolic pathways of intestinal microbiota in calves (A: MR And CR; B: MR And H23; C: MR And H25; D: MR And H29; E: MR And CST) and heat map of combined analysis of differential flora and differential metabolism (F: MR And CR; G: MR And H23; H: MR And H25; I: MR And H29; J: MR And CST). (TIF)

S1 Raw Gel Original uncropped images of Western blots and gel electrophoresis. (PDF)

## References

[1] Li ZC, Zhang YL, Song XL, Zhou YH, Liu ZZ, Ayanniyi OO, et al. Prevalence and molecular characterization of Cryptosporidium spp. from domestic pigeons (Columba livia domestica) in Anhui, China. Pol J Vet Sci. 2025;28(1):143–50. doi: 10.24425/pjvs.2025.154022 40135476

[2] Çelik B, Çelik ÖY, Ayan A, Kılınç ÖO, Akyıldız G, İrak K, et al. Occurence and genotype distribution of Cryptosporidium spp., and Giardia duodenalis in sheep in Siirt, Turkey. Polish Journal of Veterinary Sciences. 2023;26(3). 10.24425/pjvs.2023.145040 37727051

[3] Coşkun MR, Şahin M. Prevalence of neonatal calf diarrhea caused by Escherichia coli and investigation of virulence factors, serotypes, and antibiotic susceptibility. Pol J Vet Sci. 2023;26(3):335–41. doi: 10.24425/pjvs.2023.145058 37727028

[4] Ahmedin UM, Assen AA. Calf morbidity, mortality, and management practices in dairy farms in Jimma City, Southwestern Ethiopia. BMC Vet Res. 2023;19(1):249. doi: 10.1186/s12917-023-03815-w 38017486 PMC10683357

[5] Jang D-H, Cho H-C, Park Y-J, Park J, Choi K-S. First report of Cryptosporidium andersoni and risk factors associated with the occurrence of Cryptosporidium spp. in pre-weaned native Korean calves with diarrhea. Front Vet Sci. 2023;10:1145096. doi: 10.3389/fvets.2023.1145096 37026096 PMC10070877

[6] Gharpure R, Perez A, Miller AD, Wikswo ME, Silver R, Hlavsa MC. Cryptosporidiosis Outbreaks - United States, 2009-2017. MMWR Morb Mortal Wkly Rep. 2019;68:568–72. 10.15585/mmwr.mm6825a3 PMC6597118 31246941

[7] Thomson S, Hamilton CA, Hope JC, Katzer F, Mabbott NA, Morrison LJ, et al. Bovine cryptosporidiosis: Impact, host-parasite interaction and control strategies. Vet Res. 2017;48(1):42. doi: 10.1186/s13567-017-0447-0 28800747 PMC5553596

[8] Khalil IA, Troeger C, Rao PC, Blacker BF, Brown A, Brewer TG, et al. Morbidity, mortality, and long-term consequences associated with diarrhoea from Cryptosporidium infection in children younger than 5 years: A meta-analyses study. Lancet Glob Health. 2018;6(7):e758–68. doi: 10.1016/S2214-109X(18)30283-3 29903377 PMC6005120

[9] Kotloff KL, Nasrin D, Blackwelder WC, Wu Y, Farag T, Panchalingham S, et al. The incidence, aetiology, and adverse clinical consequences of less severe diarrhoeal episodes among infants and children residing in low-income and middle-income countries: A 12-month case-control study as a follow-on to the Global Enteric Multicenter Study (GEMS). Lancet Glob Health. 2019;7(5):e568–84. doi: 10.1016/S2214-109X(19)30076-2 31000128 PMC6484777

[10] Rossignol J-F. Nitazoxanide in the treatment of acquired immune deficiency syndrome-related cryptosporidiosis: Results of the United States compassionate use program in 365 patients. Aliment Pharmacol Ther. 2006;24(5):887–94. doi: 10.1111/j.1365-2036.2006.03033.x 16918894

[11] Amadi B, Mwiya M, Musuku J, Watuka A, Sianongo S, Ayoub A, et al. Effect of nitazoxanide on morbidity and mortality in Zambian children with cryptosporidiosis: A randomised controlled trial. Lancet. 2002;360(9343):1375–80. doi: 10.1016/S0140-6736(02)11401-2 12423984

[12] Cohn IS, Hunter CA. Mining for crypto protection: A search for Cryptosporidium antibodies reveals antigens associated with immunity. J Clin Invest. 2023;133(16):e171966. doi: 10.1172/JCI171966 37581310 PMC10425208

[13] Luo C, Xu Y, Zhang J, Tian Q, Guo Y, Li N, et al. Cryptosporidium parvum disrupts intestinal epithelial barrier in neonatal mice through downregulation of cell junction molecules. PLoS Negl Trop Dis. 2024;18(5):e0012212. doi: 10.1371/journal.pntd.0012212 38787872 PMC11156435

[14] Cui Z, Wang L, Wang Y, Li J, Wang R, Sun M, et al. Cryptosporidium parvum gp40/15 is associated with the parasitophorous vacuole membrane and is a potential vaccine target. Microorganisms. 2020;8(3):363. doi: 10.3390/microorganisms8030363 32143441 PMC7143253

[15] Ferguson SH, Foster DM, Sherry B, Magness ST, Nielsen DM, Gookin JL. Interferon-λ3 promotes epithelial defense and barrier function against Cryptosporidium parvum infection. Cell Mol Gastroenterol Hepatol. 2019;8(1):1–20. doi: 10.1016/j.jcmgh.2019.02.007 30849550 PMC6510929

[16] Hurle GR, Brainard J, Tyler KM. Microbiome diversity is a modifiable virulence factor for cryptosporidiosis. Virulence. 2023;14(1):2273004. doi: 10.1080/21505594.2023.2273004 37872759 PMC10653618

[17] Kuo W-T, Zuo L, Odenwald MA, Madha S, Singh G, Gurniak CB, et al. The tight junction protein ZO-1 is dispensable for barrier function but critical for effective mucosal repair. Gastroenterology. 2021;161(6):1924–39. doi: 10.1053/j.gastro.2021.08.047 34478742 PMC8605999

[18] Li J, Zhang L, Wu T, Li Y, Zhou X, Ruan Z. Indole-3-propionic acid improved the intestinal barrier by enhancing epithelial barrier and mucus barrier. J Agric Food Chem. 2021;69:1487–95. 10.1021/acs.jafc.0c05205 33356219

[19] Siqueira-Neto JL, Debnath A, McCall L-I, Bernatchez JA, Ndao M, Reed SL, et al. Cysteine proteases in protozoan parasites. PLoS Negl Trop Dis. 2018;12(8):e0006512. doi: 10.1371/journal.pntd.0006512 30138453 PMC6107107

[20] Sateriale A, Gullicksrud JA, Engiles JB, McLeod BI, Kugler EM, Henao-Mejia J, et al. The intestinal parasite Cryptosporidium is controlled by an enterocyte intrinsic inflammasome that depends on NLRP6. Proc Natl Acad Sci U S A. 2021;118(2):e2007807118. doi: 10.1073/pnas.2007807118 33372132 PMC7812745

[21] Su P, Mao X, Ma J, Huang L, Yu L, Tang S, et al. ERRα promotes glycolytic metabolism and targets the NLRP3/caspase-1/GSDMD pathway to regulate pyroptosis in endometrial cancer. J Exp Clin Cancer Res. 2023;42(1):274. doi: 10.1186/s13046-023-02834-7 37864196 PMC10588109

[22] Liu Q, Yu L, Jiang F, Li M, Zhan X, Huang Y, et al. Annotation and characterization of Babesia gibsoni apicoplast genome. Parasit Vectors. 2020;13(1):209. doi: 10.1186/s13071-020-04065-7 32317011 PMC7175588

[23] Abrahamsen MS, Templeton TJ, Enomoto S, Abrahante JE, Zhu G, Lancto CA, et al. Complete genome sequence of the apicomplexan, Cryptosporidium parvum. Science. 2004;304(5669):441–5. doi: 10.1126/science.1094786 15044751

[24] Xu R, Beatty WL, Greigert V, Witola WH, Sibley LD. Multiple pathways for glucose phosphate transport and utilization support growth of Cryptosporidium parvum. Nat Commun. 2024;15(1):380. doi: 10.1038/s41467-024-44696-3 38191884 PMC10774378

[25] Abdelbaset AE, Fox BA, Karram MH, Abd Ellah MR, Bzik DJ, Igarashi M. Lactate dehydrogenase in Toxoplasma gondii controls virulence, bradyzoite differentiation, and chronic infection. PLoS One. 2017;12(3):e0173745. doi: 10.1371/journal.pone.0173745 28323833 PMC5360243

[26] Zhang H, Guo F, Zhu G. Cryptosporidium Lactate Dehydrogenase Is Associated with the Parasitophorous Vacuole Membrane and Is a Potential Target for Developing Therapeutics. PLoS Pathog. 2015;11(11):e1005250. doi: 10.1371/journal.ppat.1005250 26562790 PMC4642935

[27] Cook WJ, Senkovich O, Hernandez A, Speed H, Chattopadhyay D. Biochemical and structural characterization of Cryptosporidium parvum Lactate dehydrogenase. Int J Biol Macromol. 2015;74:608–19. doi: 10.1016/j.ijbiomac.2014.12.019 25542170

[28] Kayamba F, Faya M, Pooe OJ, Kushwaha B, Kushwaha ND, Obakachi VA, et al. Lactate dehydrogenase and malate dehydrogenase: Potential antiparasitic targets for drug development studies. Bioorg Med Chem. 2021;50:116458. doi: 10.1016/j.bmc.2021.116458 34687983

[29] Swale C, Bougdour A, Gnahoui-David A, Tottey J, Georgeault S, Laurent F, et al. Metal-captured inhibition of pre-mRNA processing activity by CPSF3 controls Cryptosporidium infection. Sci Transl Med. 2019;11(517):eaax7161. doi: 10.1126/scitranslmed.aax7161 31694928

[30] Li K, Nader SM, Zhang X, Ray BC, Kim CY, Das A, et al. Intestinal barrier. PLoS Pathog. 2019;15:e1007953. 10.1371/journal.ppat.1007953 PMC6687188 31356619

[31] Thoo L, Noti M, Krebs P. Keep calm: The intestinal barrier at the interface of peace and war. Cell Death Dis. 2019;10(11):849. doi: 10.1038/s41419-019-2086-z 31699962 PMC6838056

[32] Grosheva I, Zheng D, Levy M, Polansky O, Lichtenstein A, Golani O, et al. High-throughput screen identifies host and microbiota regulators of intestinal barrier function. Gastroenterology. 2020;159(5):1807–23. doi: 10.1053/j.gastro.2020.07.003 32653496

[33] Wang Y, Gong Y, Farid MS, Zhao C. Milk: A natural guardian for the gut barrier. J Agric Food Chem. 2024;72(15):8285–303. doi: 10.1021/acs.jafc.3c06861 38588092

[34] Groschwitz KR, Hogan SP. Intestinal barrier function: Molecular regulation and disease pathogenesis. J Allergy Clin Immunol. 2009;124(1):3–20; quiz 21–2. doi: 10.1016/j.jaci.2009.05.038 19560575 PMC4266989

[35] Zihni C, Mills C, Matter K, Balda MS. Tight junctions: From simple barriers to multifunctional molecular gates. Nat Rev Mol Cell Biol. 2016;17(9):564–80. doi: 10.1038/nrm.2016.80 27353478

[36] Wu Y, Zhang X, Liu X, Zhao Z, Tao S, Xu Q, et al. Galactooligosaccharides and Limosilactobacillus reuteri synergistically alleviate gut inflammation and barrier dysfunction by enriching Bacteroides acidifaciens for pentadecanoic acid biosynthesis. Nat Commun. 2024;15(1):9291. doi: 10.1038/s41467-024-53144-1 39468026 PMC11519483

[37] Argüello-García R, Carrero JC, Ortega-Pierres MG. Extracellular cysteine proteases of key intestinal protozoan pathogens-factors linked to virulence and pathogenicity. Int J Mol Sci. 2023;24(16):12850. doi: 10.3390/ijms241612850 37629029 PMC10454693

[38] Schüler H, Matuschewski K. Regulation of apicomplexan microfilament dynamics by a minimal set of actin-binding proteins. Traffic. 2006;7(11):1433–9. doi: 10.1111/j.1600-0854.2006.00484.x 17010119

[39] Burdette BE, Esparza AN, Zhu H, Wang S. Gasdermin D in pyroptosis. Acta Pharm Sin B. 2021;11:2768–82. 10.1016/j.apsb.2021.02.006 PMC8463274 34589396

[40] Barnett KC, Li S, Liang K, Ting JP-Y. A 360° view of the inflammasome: Mechanisms of activation, cell death, and diseases. Cell. 2023;186(11):2288–312. doi: 10.1016/j.cell.2023.04.025 37236155 PMC10228754

[41] Mules TC, Inns S, Le Gros G. Helminths’ therapeutic potential to treat intestinal barrier dysfunction. Allergy. 2023;78(11):2892–905. doi: 10.1111/all.15812 37449458

[42] Wekerle H. Brain autoimmunity and intestinal microbiota: 100 trillion game changers. Trends in Immunology. 2017;38:483–97. 10.1016/j.it.2017.03.008 28601415

[43] He X, Gao J, Peng L, Hu T, Wan Y, Zhou M, et al. Bacterial O-GlcNAcase genes abundance decreases in ulcerative colitis patients and its administration ameliorates colitis in mice. Gut. 2021;70(10):1872–83. doi: 10.1136/gutjnl-2020-322468 33310751 PMC8458092

[44] Magne F, Gotteland M, Gauthier L, Zazueta A, Pesoa S, Navarrete P, et al. The firmicutes/bacteroidetes ratio: A relevant marker of gut dysbiosis in obese patients?. Nutrients. 2020;12. 10.3390/nu12051474 PMC7285218 32438689

[45] Zafar H, Saier MH. Gut Bacteroides species in health and disease. Gut Microbes. 2021;13(1):1–20. doi: 10.1080/19490976.2020.1848158 33535896 PMC7872030

[46] Wang X, Cai Z, Wang Q, Wu C, Sun Y, Wang Z, et al. Bacteroides methylmalonyl-CoA mutase produces propionate that promotes intestinal goblet cell differentiation and homeostasis. Cell Host Microbe. 2024;32(1):63-78.e7. doi: 10.1016/j.chom.2023.11.005 38056459

[47] Flint HJ, Scott KP, Duncan SH, Louis P, Forano E. Microbial degradation of complex carbohydrates in the gut. Gut Microbes. 2012;3(4):289–306. doi: 10.4161/gmic.19897 22572875 PMC3463488

[48] El Kaoutari A, Armougom F, Gordon JI, Raoult D, Henrissat B. The abundance and variety of carbohydrate-active enzymes in the human gut microbiota. Nat Rev Microbiol. 2013;11(7):497–504. doi: 10.1038/nrmicro3050 23748339

[49] Doifode T, Giridharan VV, Generoso JS, Bhatti G, Collodel A, Schulz PE, et al. The impact of the microbiota-gut-brain axis on Alzheimer’s disease pathophysiology. Pharmacol Res. 2021;164:105314. doi: 10.1016/j.phrs.2020.105314 33246175

[50] Dong H, Chen X, Zhao X, Zhao C, Mehmood K, Kulyar MF-E-A, et al. Intestine microbiota and SCFAs response in naturally Cryptosporidium-infected plateau yaks. Front Cell Infect Microbiol. 2023;13:1105126. doi: 10.3389/fcimb.2023.1105126 36936759 PMC10014559

[51] Chen X, Zhao X, Zhao C, Ashfaq H, Fakhar-E-Alam Kulyar M, Bhutta ZA, et al. Cryptosporidium infection induced the dropping of SCFAS and dysbiosis in intestinal microbiome of Tibetan pigs. Microb Pathog. 2023;174:105922. doi: 10.1016/j.micpath.2022.105922 36462579

[52] Wang Y, Li X, Chen X, Kulyar MF-E-A, Duan K, Li H, et al. Gut Fungal Microbiome Responses to Natural Cryptosporidium Infection in Horses. Front Microbiol. 2022;13:877280. doi: 10.3389/fmicb.2022.877280 35875530 PMC9298756

[53] Xu L, Qiu B, Ba F, Zhang S, Han S, Chen H, et al. Synergistic effects of Ligilactobacillus salivarius Li01 and psyllium husk prevent mice from developing loperamide-induced constipation. Food Funct. 2024;15(24):11934–48. doi: 10.1039/d4fo04444d 39545778

[54] Fang C, Cheng J, Jia W, Xu Y. Akkermansia muciniphila ameliorates alcoholic liver disease in experimental mice by regulating serum metabolism and improving gut dysbiosis. Metabolites. 2023;13(10):1057. doi: 10.3390/metabo13101057 37887381 PMC10608788

[55] Li G, Lin J, Zhang C, Gao H, Lu H, Gao X, et al. Microbiota metabolite butyrate constrains neutrophil functions and ameliorates mucosal inflammation in inflammatory bowel disease. Gut Microbes. 2021;13(1):1968257. doi: 10.1080/19490976.2021.1968257 34494943 PMC8437544

[56] Guo Q, Chen L, Liu Z, Zheng B. Chlorogenic acid/linoleic acid-fortified wheat-resistant starch ameliorates high-fat diet-induced gut barrier damage by modulating gut metabolism. J Agric Food Chem. 2024;72(20):11759–72. doi: 10.1021/acs.jafc.4c01595 38738668

[57] Sakamoto M, Ikeyama N, Yuki M, Ohkuma M. Draft Genome Sequence of Faecalimonas umbilicata JCM 30896T, an Acetate-Producing Bacterium Isolated from Human Feces. Microbiol Resour Announc. 2018;7(9):e01091-18. doi: 10.1128/MRA.01091-18 30533935 PMC6256527

[58] Sun W, Cui Y, Zhang X, Wang Y, Zhang Z, Ding X, et al. Effects of gabexate mesylate on the gut microbiota and metabolomics in rats with sepsis. J Inflamm Res. 2022;15:6581–94. doi: 10.2147/JIR.S392060 36506782 PMC9733569

[59] Crost EH, Coletto E, Bell A, Juge N. Ruminococcus gnavus: Friend or foe for human health. FEMS Microbiol Rev. 2023;47(2):fuad014. doi: 10.1093/femsre/fuad014 37015876 PMC10112845

[60] Klimczak S, Packi K, Rudek A, Wenclewska S, Kurowski M, Kurczabińska D, et al. The Influence of the Protozoan Giardia lamblia on the Modulation of the Immune System and Alterations in Host Glucose and Lipid Metabolism. Int J Mol Sci. 2024;25(16):8627. doi: 10.3390/ijms25168627 39201314 PMC11354543

[61] Miao C, Wang L, Wang H, Shen Y, Man C, Zhang W, et al. Lacticaseibacillus plantarum postbiotics prepared by the combined technique of pasteurization and ultrasound: Effective measures to alleviate obesity based on the SCFAs-GPR41/GPR43 signaling pathway. Food Funct. 2024;15(22):11005–19. doi: 10.1039/d4fo03591g 39420807

[62] Fu X, Liu Z, Zhu C, Mou H, Kong Q. Nondigestible carbohydrates, butyrate, and butyrate-producing bacteria. Crit Rev Food Sci Nutr. 2019;59(sup1):S130–52. doi: 10.1080/10408398.2018.1542587 30580556

[63] Qu R, Zhang Y, Ma Y, Zhou X, Sun L, Jiang C, et al. Role of the gut microbiota and its metabolites in tumorigenesis or development of colorectal cancer. Adv Sci (Weinh). 2023;10(23):e2205563. doi: 10.1002/advs.202205563 37263983 PMC10427379

[64] Astorga J, Gasaly N, Dubois-Camacho K, De la Fuente M, Landskron G, Faber KN, et al. The role of cholesterol and mitochondrial bioenergetics in activation of the inflammasome in IBD. Front Immunol. 2022;13:1028953. doi: 10.3389/fimmu.2022.1028953 36466902 PMC9716353

[65] Salway JG. The krebs uric acid cycle: A forgotten krebs cycle. Trends Biochem Sci. 2018;43(11):847–9. doi: 10.1016/j.tibs.2018.04.012 29807701

[66] de Oliveira EC, Tibúrcio R, Duarte G, Lago A, de Melo L, Nunes S, et al. Pharmacological inhibition of key metabolic pathways attenuates Leishmania spp infection in macrophages. PLoS Negl Trop Dis. 2025;19(1):e0012763. doi: 10.1371/journal.pntd.0012763 39775223 PMC11756801

[67] Chen Z, Sun X, Li X, Liu N. Oleoylethanolamide alleviates hyperlipidaemia-mediated vascular calcification via attenuating mitochondrial DNA stress triggered autophagy-dependent ferroptosis by activating PPARα. Biochem Pharmacol. 2023;208:115379. doi: 10.1016/j.bcp.2022.115379 36525991

[68] Lennicke C, Cochemé HM. Redox metabolism: ROS as specific molecular regulators of cell signaling and function. Mol Cell. 2021;81(18):3691–707. doi: 10.1016/j.molcel.2021.08.018 34547234

[69] Youm Y-H, Nguyen KY, Grant RW, Goldberg EL, Bodogai M, Kim D, et al. The ketone metabolite β-hydroxybutyrate blocks NLRP3 inflammasome-mediated inflammatory disease. Nat Med. 2015;21(3):263–9. doi: 10.1038/nm.3804 25686106 PMC4352123

