# Supplementary material for: Novel inhibitors against Cryptosporidium parvum through blocking its energy metabolism
Source: PLoS Negl Trop Dis. 2026 Oct 5;20(10):e0014742. doi: 10.1371/journal.pntd.0014742 (PMC13652328; doi:10.1371/journal.pntd.0014742)

Caspase-1

Marker CR MR H23 H25 H29 CST

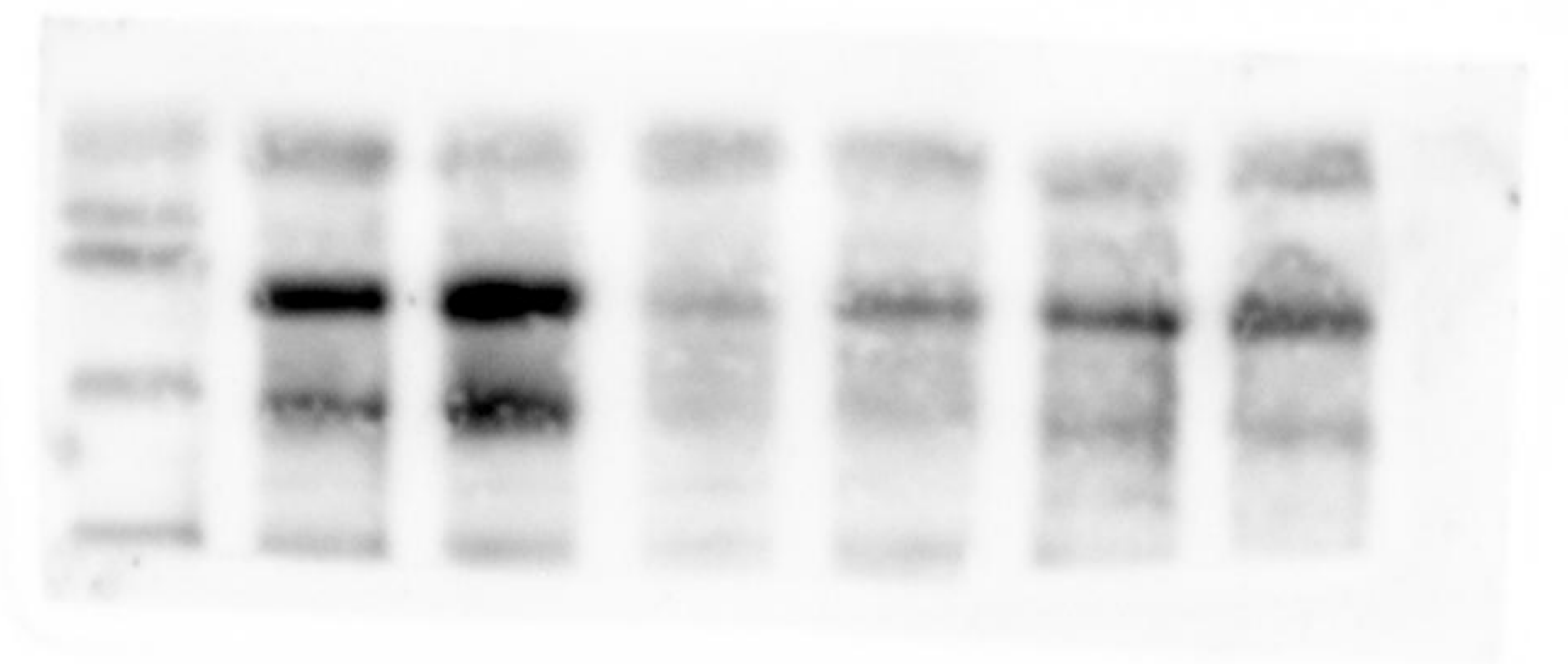

Claudin-1

Marker CR MR H23 H25 H29 CST

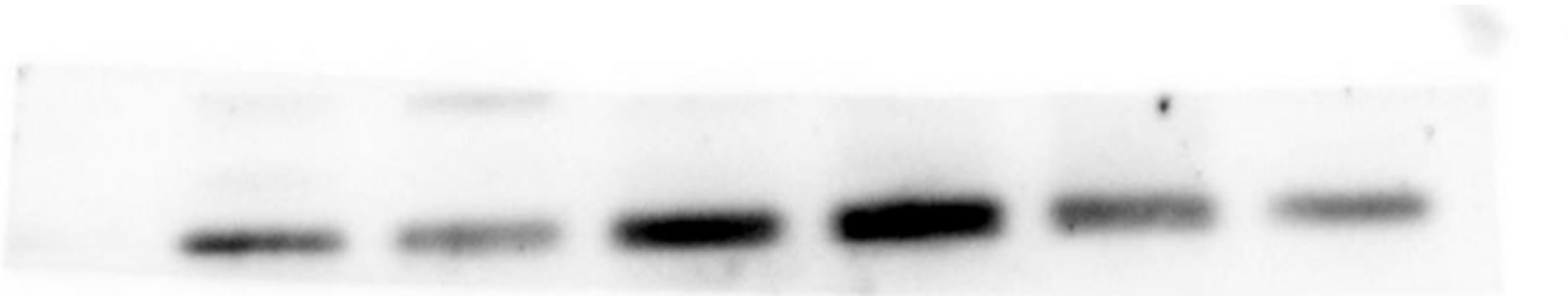

GAPDH

Marker CR MR H23 H25 H29 CST

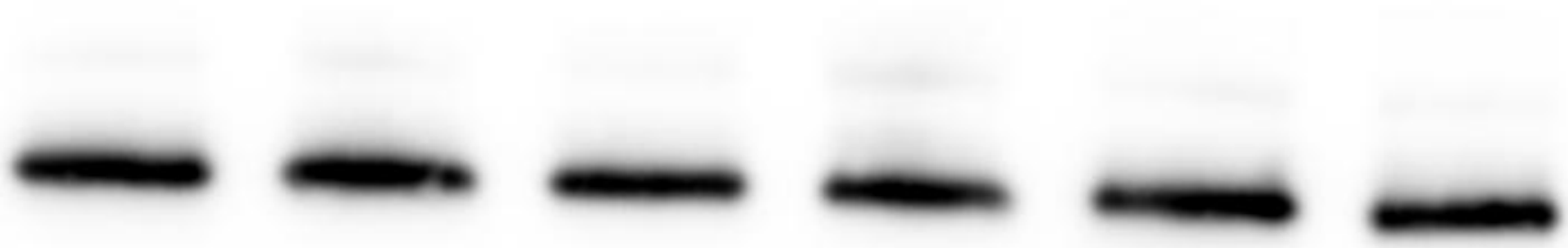

Occludin

Marker CR MR H23 H25 H29 CST

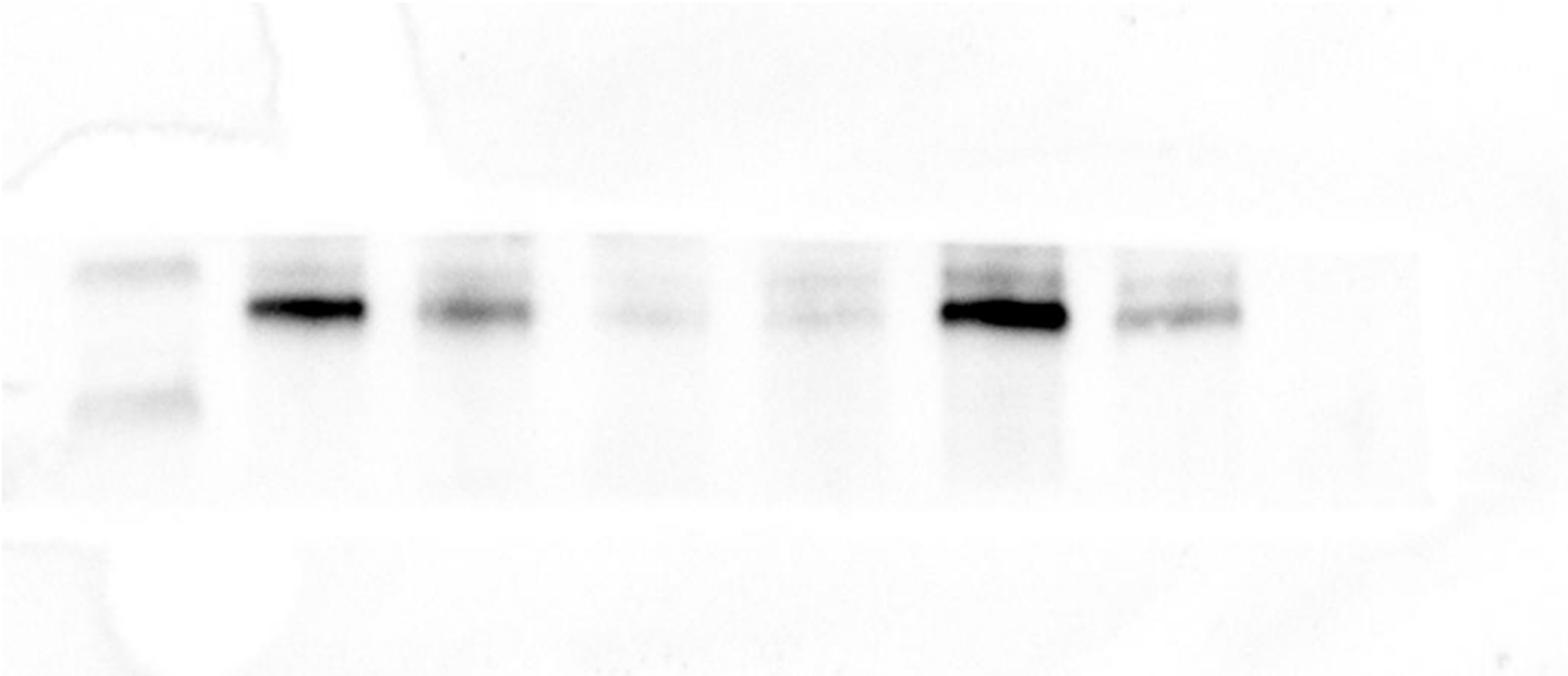

ZO-1

Marker CR MR H23 H25 H29 CST

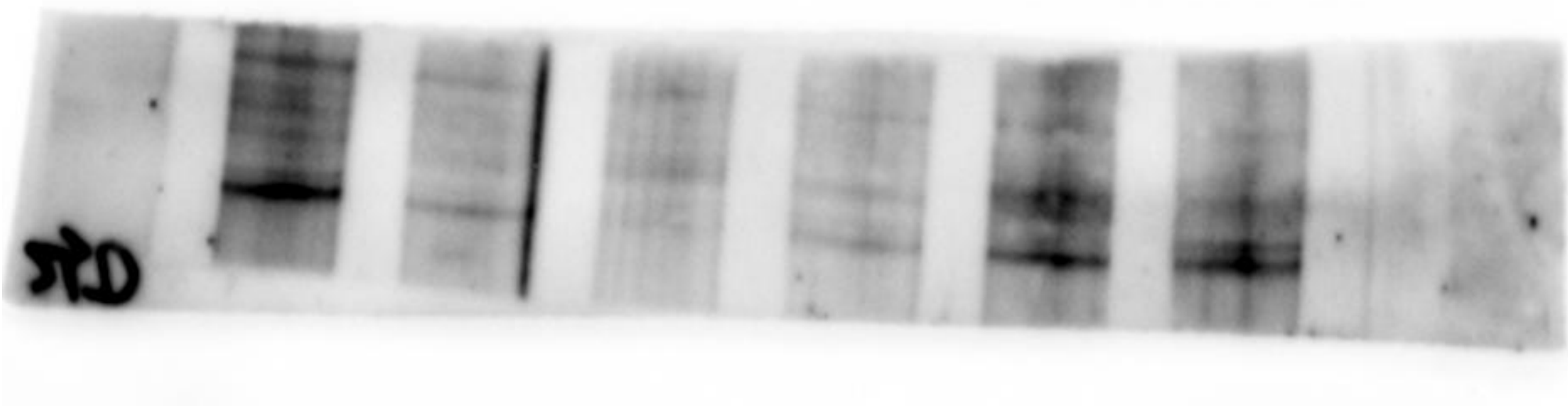

Supplement: Supplementary file 1 [file pntd.0014742.s009.pdf]
