# Supplementary material for: Novel inhibitors against Cryptosporidium parvum through blocking its energy metabolism
Source: PLoS Negl Trop Dis. 2026 Oct 5;20(10):e0014742. doi: 10.1371/journal.pntd.0014742 (PMC13652328; doi:10.1371/journal.pntd.0014742)
Supplement: Supplementary file 2 [file pntd.0014742.s001.docx]

**Supplementary Table 1** Screening results of molecular docking of derivatives

| Number | Cp-LDH  Score | HLDH  Score | D-value | Number | Cp-LDH  Score | HLDH  Score | D-value |
| --- | --- | --- | --- | --- | --- | --- | --- |
| 413 | -10.4 | -7 | -3.4 | 308 | -9.5 | -5.7 | -3.8 |
| 29 | -10.3 | -7 | -3.3 | 488 | -9.5 | -5.7 | -3.8 |
| 221 | -10.3 | -8.1 | -2.2 | 500 | -9.5 | -5.8 | -3.7 |
| 26 | -10.1 | -7.8 | -2.3 | 506 | -9.5 | -7.8 | -1.7 |
| 35 | -10.1 | -8 | -2.1 | 518 | -9.5 | -5.5 | -4 |
| 41 | -10.1 | -8.1 | -2 | 584 | -9.5 | -5.5 | -4 |
| 44 | -10.1 | -5.8 | -4.3 | 602 | -9.5 | -8.1 | -1.4 |
| 1181 | -10.1 | -7.8 | -2.3 | 641 | -9.5 | -5.6 | -3.9 |
| 1277 | -10.1 | -7.6 | -2.5 | 1178 | -9.5 | -7.7 | -1.8 |
| 20 | -10 | -5.8 | -4.2 | 1184 | -9.5 | -7.7 | -1.8 |
| 47 | -10 | -6 | -4 | 1187 | -9.5 | -6.8 | -2.7 |
| 65 | -10 | -5.9 | -4.1 | 1274 | -9.5 | -6.6 | -2.9 |
| 218 | -10 | -7.2 | -2.8 | 212 | -9.4 | -6.1 | -3.3 |
| 23 | -9.9 | -5.8 | -4.1 | 290 | -9.4 | -7.8 | -1.6 |
| 620 | -9.9 | -5.5 | -4.4 | 317 | -9.4 | -6.8 | -2.6 |
| 17 | -9.8 | -5.5 | -4.3 | 323 | -9.4 | -7.8 | -1.6 |
| 416 | -9.8 | -6.9 | -2.9 | 344 | -9.4 | -5.9 | -3.5 |
| 419 | -9.8 | -8 | -1.8 | 422 | -9.4 | -5.3 | -4.1 |
| 509 | -9.8 | -7.6 | -2.2 | 482 | -9.4 | -7.7 | -1.7 |
| 524 | -9.8 | -5.6 | -4.2 | 503 | -9.4 | -5.7 | -3.7 |
| 1373 | -9.8 | -6.5 | -3.3 | 515 | -9.4 | -7.8 | -1.6 |
| 32 | -9.7 | -8 | -1.7 | 569 | -9.4 | -5.6 | -3.8 |
| 230 | -9.7 | -6.2 | -3.5 | 1076 | -9.4 | -6.1 | -3.3 |
| 320 | -9.7 | -6.8 | -2.9 | 1085 | -9.4 | -7.8 | -1.6 |
| 335 | -9.7 | -5.6 | -4.1 | 1094 | -9.4 | -5.8 | -3.6 |
| 410 | -9.7 | -7 | -2.7 | 599 | -9.3 | -5.8 | -3.5 |
| 512 | -9.7 | -7.8 | -1.9 | 611 | -9.3 | -8 | -1.3 |
| 527 | -9.7 | -5.7 | -4 | 653 | -9.3 | -5.8 | -3.5 |
| 608 | -9.7 | -7.6 | -2.1 | 665 | -9.3 | -5.6 | -3.7 |
| 1088 | -9.7 | -6.9 | -2.8 | 1049 | -9.3 | -5.7 | -3.6 |
| 11 | -9.6 | -6 | -3.6 | 1259 | -9.3 | -5.9 | -3.4 |
| 161 | -9.6 | -5.8 | -3.8 | 53 | -9.2 | -6.1 | -3.1 |
| 209 | -9.6 | -5.9 | -3.7 | 176 | -9.2 | -6.2 | -3 |
| 314 | -9.6 | -7 | -2.6 | 497 | -9.2 | -5.6 | -3.6 |
| 428 | -9.6 | -5.6 | -4 | 560 | -9.2 | -5.4 | -3.8 |
| 431 | -9.6 | -5.8 | -3.8 | 656 | -9.2 | -5.9 | -3.3 |
| 521 | -9.6 | -5.5 | -4.1 | 662 | -9.2 | -5.8 | -3.4 |
| 617 | -9.6 | -5.7 | -3.9 | 1169 | -9.2 | -5.4 | -3.8 |
| 1103 | -9.6 | -5.6 | -4 | 1361 | -9.2 | -5.6 | -3.6 |
| 1283 | -9.6 | -6.8 | -2.8 | 197 | -9.1 | -7.1 | -2 |
| 1292 | -9.6 | -5.6 | -4 | 245 | -9.1 | -6.2 | -2.9 |
| 643 | -5.5 | -6.6 | 1.1 | 275 | -9.1 | -6.4 | -2.7 |
| 650 | -9.1 | -5.6 | -3.5 | 1247 | -7 | -5.7 | -1.3 |
| 677 | -9.1 | -8 | -1.1 | 1268 | -7 | -6 | -1 |
| 1058 | -9.1 | -6 | -3.1 | 1286 | -7 | -5.4 | -1.6 |
| 1073 | -9.1 | -5.9 | -3.2 | 1295 | -7 | -5.7 | -1.3 |
| 1097 | -9.1 | -5.6 | -3.5 | 1319 | -7 | -5.8 | -1.2 |
| 1256 | -9.1 | -5.6 | -3.5 | 1337 | -7 | -5.5 | -1.5 |
| 1280 | -9.1 | -6.6 | -2.5 | 1385 | -7 | -5.2 | -1.8 |
| 1376 | -9.1 | -6.6 | -2.5 | 1397 | -7 | -5.8 | -1.2 |
| 2 | -9 | -7.7 | -1.3 | 1 | -6.9 | -7.2 | 0.3 |
| 56 | -9 | -5.8 | -3.2 | 100 | -6.9 | -7.3 | 0.4 |
| 194 | -9 | -5.9 | -3.1 | 152 | -6.9 | -5.9 | -1 |
| 302 | -9 | -5.3 | -3.7 | 166 | -6.9 | -5.8 | -1.1 |
| 593 | -9 | -5.6 | -3.4 | 190 | -6.9 | -5.3 | -1.6 |
| 1379 | -9 | -6.7 | -2.3 | 196 | -6.9 | -7.6 | 0.7 |
| 590 | -8.9 | -5.3 | -3.6 | 239 | -6.9 | -6.4 | -0.5 |
| 149 | -8.8 | -6 | -2.8 | 244 | -6.9 | -7.8 | 0.9 |
| 215 | -8.8 | -6.5 | -2.3 | 263 | -6.9 | -6.4 | -0.5 |
| 217 | -8.8 | -7.5 | -1.3 | 286 | -6.9 | -7.4 | 0.5 |
| 220 | -8.8 | -7.7 | -1.1 | 287 | -6.9 | -6.3 | -0.6 |
| 226 | -8.8 | -7.5 | -1.3 | 337 | -6.9 | -5.8 | -1.1 |
| 1034 | -8.8 | -5.8 | -3 | 340 | -6.9 | -5.9 | -1 |
| 206 | -8.7 | -6.1 | -2.6 | 346 | -6.9 | -7.1 | 0.2 |
| 281 | -8.7 | -6 | -2.7 | 364 | -6.9 | -7 | 0.1 |
| 293 | -8.7 | -5.9 | -2.8 | 388 | -6.9 | -7.4 | 0.5 |
| 389 | -8.7 | -6.1 | -2.6 | 394 | -6.9 | -5.5 | -1.4 |
| 485 | -8.7 | -8 | -0.7 | 433 | -6.9 | -5.4 | -1.5 |
| 581 | -8.7 | -5.6 | -3.1 | 442 | -6.9 | -5.5 | -1.4 |
| 1010 | -8.7 | -5.6 | -3.1 | 511 | -6.9 | -6.7 | -0.2 |
| 1331 | -8.7 | -5.9 | -2.8 | 542 | -6.9 | -5.5 | -1.4 |
| 331 | -8.6 | -5.5 | -3.1 | 575 | -6.9 | -5.5 | -1.4 |
| 427 | -8.6 | -5.7 | -2.9 | 628 | -6.9 | -7.2 | 0.3 |
| 517 | -8.6 | -5.4 | -3.2 | 1045 | -6.9 | -5.4 | -1.5 |
| 28 | -8.5 | -7.3 | -1.2 | 1051 | -6.9 | -6.7 | -0.2 |
| 43 | -8.5 | -6.3 | -2.2 | 1063 | -6.9 | -6.1 | -0.8 |
| 158 | -8.5 | -6 | -2.5 | 1114 | -6.9 | -5.8 | -1.1 |
| 325 | -8.5 | -5.8 | -2.7 | 1129 | -6.9 | -6.9 | 0 |
| 421 | -8.5 | -6.9 | -1.6 | 1172 | -6.9 | -6.3 | -0.6 |
| 430 | -8.5 | -5.8 | -2.7 | 1207 | -6.9 | -5.1 | -1.8 |
| 491 | -8.5 | -5.8 | -2.7 | 1262 | -6.9 | -5.3 | -1.6 |
| 526 | -8.5 | -5.8 | -2.7 | 1306 | -6.9 | -5.4 | -1.5 |
| 578 | -8.5 | -5.7 | -2.8 | 1333 | -6.9 | -5 | -1.9 |
| 587 | -8.5 | -5.9 | -2.6 | 1340 | -6.9 | -5.6 | -1.3 |
| 616 | -8.5 | -5.8 | -2.7 | 1400 | -6.9 | -6 | -0.9 |
| 1087 | -8.5 | -7.1 | -1.4 | 1402 | -6.9 | -5.4 | -1.5 |
| 1096 | -8.5 | -6.1 | -2.4 | 1435 | -6.9 | -5 | -1.9 |
| 1099 | -8.5 | -5.9 | -2.6 | 1436 | -6.9 | -5.4 | -1.5 |
| 1148 | -8.5 | -6.1 | -2.4 | 10 | -6.8 | -5.7 | -1.1 |
| 1151 | -8.5 | -5.7 | -2.8 | 46 | -6.8 | -5.4 | -1.4 |
| 1157 | -8.5 | -5.9 | -2.6 | 61 | -6.8 | -7 | 0.2 |
| 1349 | -8.5 | -7.8 | -0.7 | 103 | -6.8 | -5.8 | -1 |
| 16 | -8.4 | -7 | -1.4 | 110 | -6.8 | -5.8 | -1 |
| 113 | -8.4 | -5.5 | -2.9 | 151 | -6.8 | -6.9 | 0.1 |
| 121 | -8.4 | -7.1 | -1.3 | 235 | -6.8 | -6.5 | -0.3 |
| 124 | -8.4 | -7.2 | -1.2 | 251 | -6.8 | -6.3 | -0.5 |
| 130 | -8.4 | -7.4 | -1 | 254 | -6.8 | -5.9 | -0.9 |
| 170 | -8.4 | -5.9 | -2.5 | 272 | -6.8 | -6 | -0.8 |
| 211 | -8.4 | -6.5 | -1.9 | 278 | -6.8 | -6.3 | -0.5 |
| 224 | -8.4 | -7.1 | -1.3 | 298 | -6.8 | -5.5 | -1.3 |
| 227 | -8.4 | -7.4 | -1 | 361 | -6.8 | -6.6 | -0.2 |
| 292 | -8.4 | -7 | -1.4 | 370 | -6.8 | -6.8 | 0 |
| 319 | -8.4 | -6.3 | -2.1 | 400 | -6.8 | -7.2 | 0.4 |
| 415 | -8.4 | -6.8 | -1.6 | 406 | -6.8 | -5.4 | -1.4 |
| 424 | -8.4 | -5.9 | -2.5 | 460 | -6.8 | -6.9 | 0.1 |
| 484 | -8.4 | -7.2 | -1.2 | 466 | -6.8 | -5.3 | -1.5 |
| 619 | -8.4 | -6.4 | -2 | 472 | -6.8 | -5.4 | -1.4 |
| 629 | -8.4 | -5.8 | -2.6 | 572 | -6.8 | -5.6 | -1.2 |
| 1189 | -8.4 | -6.8 | -1.6 | 634 | -6.8 | -5.3 | -1.5 |
| 1192 | -8.4 | -6.8 | -1.6 | 1060 | -6.8 | -6.1 | -0.7 |
| 1285 | -8.4 | -7 | -1.4 | 1111 | -6.8 | -5.9 | -0.9 |
| 1381 | -8.4 | -5.5 | -2.9 | 1120 | -6.8 | -5.9 | -0.9 |
| 1384 | -8.4 | -6.5 | -1.9 | 1138 | -6.8 | -5.3 | -1.5 |
| 1390 | -8.4 | -6.7 | -1.7 | 1351 | -6.8 | -5.5 | -1.3 |
| 19 | -8.3 | -5.3 | -3 | 1418 | -6.8 | -5.6 | -1.2 |
| 40 | -8.3 | -5.9 | -2.4 | 155 | -6.7 | -5.8 | -0.9 |
| 139 | -8.3 | -5.8 | -2.5 | 214 | -6.7 | -6.1 | -0.6 |
| 223 | -8.3 | -7.6 | -0.7 | 232 | -6.7 | -6.4 | -0.3 |
| 304 | -8.3 | -7.2 | -1.1 | 247 | -6.7 | -5.6 | -1.1 |
| 412 | -8.3 | -7.2 | -1.1 | 259 | -6.7 | -5.5 | -1.2 |
| 493 | -8.3 | -6.6 | -1.7 | 260 | -6.7 | -6.4 | -0.3 |
| 580 | -8.3 | -7.3 | -1 | 284 | -6.7 | -5.9 | -0.8 |
| 592 | -8.3 | -7.2 | -1.1 | 328 | -6.7 | -5.4 | -1.3 |
| 613 | -8.3 | -5.8 | -2.5 | 385 | -6.7 | -7 | 0.3 |
| 631 | -8.3 | -7 | -1.3 | 458 | -6.7 | -6.2 | -0.5 |
| 1000 | -8.3 | -5.7 | -2.6 | 529 | -6.7 | -5.4 | -1.3 |
| 1006 | -8.3 | -6.2 | -2.1 | 535 | -6.7 | -7 | 0.3 |
| 1069 | -8.3 | -5.8 | -2.5 | 553 | -6.7 | -6.6 | -0.1 |
| 1072 | -8.3 | -6.7 | -1.6 | 556 | -6.7 | -6.6 | -0.1 |
| 1180 | -8.3 | -6.8 | -1.5 | 625 | -6.7 | -7 | 0.3 |
| 1198 | -8.3 | -5 | -3.3 | 649 | -6.7 | -6.7 | 0 |
| 1250 | -8.3 | -5.5 | -2.8 | 659 | -6.7 | -5.8 | -0.9 |
| 1252 | -8.3 | -7.3 | -1 | 670 | -6.7 | -7.1 | 0.4 |
| 1276 | -8.3 | -6.8 | -1.5 | 1075 | -6.7 | -5.8 | -0.9 |
| 1288 | -8.3 | -6.7 | -1.6 | 1078 | -6.7 | -5.6 | -1.1 |
| 1372 | -8.3 | -7 | -1.3 | 1105 | -6.7 | -6.1 | -0.6 |
| 1387 | -8.3 | -6.4 | -1.9 | 1115 | -6.7 | -6.1 | -0.6 |
| 13 | -8.2 | -5.7 | -2.5 | 1123 | -6.7 | -6.1 | -0.6 |
| 25 | -8.2 | -7 | -1.2 | 1126 | -6.7 | -5.3 | -1.4 |
| 101 | -8.2 | -7.7 | -0.5 | 1144 | -6.7 | -5.3 | -1.4 |
| 122 | -8.2 | -7 | -1.2 | 1201 | -6.7 | -5.4 | -1.3 |
| 127 | -8.2 | -6.9 | -1.3 | 1210 | -6.7 | -6.8 | 0.1 |
| 128 | -8.2 | -7 | -1.2 | 1226 | -6.7 | -5.9 | -0.8 |
| 131 | -8.2 | -7 | -1.2 | 1231 | -6.7 | -6.9 | 0.2 |
| 301 | -8.2 | -6.8 | -1.4 | 1258 | -6.7 | -5.5 | -1.2 |
| 397 | -8.2 | -5.2 | -3 | 1297 | -6.7 | -5.6 | -1.1 |
| 1165 | -8.2 | -5.3 | -2.9 | 1354 | -6.7 | -5.5 | -1.2 |
| 1183 | -8.2 | -6.1 | -2.1 | 1393 | -6.7 | -7.3 | 0.6 |
| 1279 | -8.2 | -6.2 | -2 | 1403 | -6.7 | -5.9 | -0.8 |
| 1375 | -8.2 | -6.8 | -1.4 | 1423 | -6.7 | -5.2 | -1.5 |
| 38 | -8.1 | -5.3 | -2.8 | 1427 | -6.7 | -5.9 | -0.8 |
| 265 | -8.1 | -7.2 | -0.9 | 22 | -6.6 | -5.8 | -0.8 |
| 499 | -8.1 | -5.2 | -2.9 | 37 | -6.6 | -5.6 | -1 |
| 559 | -8.1 | -6.7 | -1.4 | 67 | -6.6 | -5.3 | -1.3 |
| 589 | -8.1 | -5.3 | -2.8 | 115 | -6.6 | -5.5 | -1.1 |
| 595 | -8.1 | -5.1 | -3 | 163 | -6.6 | -5.1 | -1.5 |
| 598 | -8.1 | -6.8 | -1.3 | 173 | -6.6 | -5.8 | -0.8 |
| 1084 | -8.1 | -7.1 | -1 | 185 | -6.6 | -6.1 | -0.5 |
| 1135 | -8.1 | -5.3 | -2.8 | 187 | -6.6 | -5.7 | -0.9 |
| 1261 | -8.1 | -5.3 | -2.8 | 283 | -6.6 | -5.5 | -1.1 |
| 34 | -8 | -7.4 | -0.6 | 352 | -6.6 | -6.7 | 0.1 |
| 172 | -8 | -5.6 | -2.4 | 376 | -6.6 | -6.8 | 0.2 |
| 268 | -8 | -7.2 | -0.8 | 448 | -6.6 | -6.8 | 0.2 |
| 310 | -8 | -5.8 | -2.2 | 454 | -6.6 | -5.6 | -1 |
| 544 | -8 | -6.9 | -1.1 | 490 | -6.6 | -7.1 | 0.5 |
| 640 | -8 | -7.1 | -0.9 | 622 | -6.6 | -5.6 | -1 |
| 655 | -8 | -6.6 | -1.4 | 646 | -6.6 | -5 | -1.6 |
| 676 | -8 | -7.7 | -0.3 | 647 | -6.6 | -5.8 | -0.8 |
| 1154 | -8 | -7.7 | -0.3 | 671 | -6.6 | -5.8 | -0.8 |
| 1357 | -8 | -6.1 | -1.9 | 1007 | -6.6 | -5.5 | -1.1 |
| 52 | -7.9 | -7.9 | 0 | 1033 | -6.6 | -5.7 | -0.9 |
| 178 | -7.9 | -5.8 | -2.1 | 1066 | -6.6 | -6 | -0.6 |
| 182 | -7.9 | -6.1 | -1.8 | 1102 | -6.6 | -6.1 | -0.5 |
| 203 | -7.9 | -5.8 | -2.1 | 1109 | -6.6 | -6 | -0.6 |
| 236 | -7.9 | -5.9 | -2 | 1117 | -6.6 | -5.8 | -0.8 |
| 398 | -7.9 | -5.9 | -2 | 1124 | -6.6 | -5.6 | -1 |
| 1036 | -7.9 | -5.6 | -2.3 | 1141 | -6.6 | -5.3 | -1.3 |
| 1082 | -7.9 | -6.9 | -1 | 1150 | -6.6 | -5.2 | -1.4 |
| 1171 | -7.9 | -5 | -2.9 | 1153 | -6.6 | -7.1 | 0.5 |
| 1267 | -7.9 | -5.3 | -2.6 | 1156 | -6.6 | -6.7 | 0.1 |
| 1414 | -7.9 | -5.1 | -2.8 | 1162 | -6.6 | -5.4 | -1.2 |
| 31 | -7.8 | -7 | -0.8 | 1234 | -6.6 | -5.2 | -1.4 |
| 200 | -7.8 | -6.2 | -1.6 | 1255 | -6.6 | -6.7 | 0.1 |
| 1091 | -7.8 | -7.8 | 0 | 1294 | -6.6 | -6.7 | 0.1 |
| 1363 | -7.8 | -5.2 | -2.6 | 1321 | -6.6 | -6.7 | 0.1 |
| 4 | -7.7 | -7.4 | -0.3 | 1330 | -6.6 | -5 | -1.6 |
| 188 | -7.7 | -5.6 | -2.1 | 1343 | -6.6 | -5.8 | -0.8 |
| 193 | -7.7 | -7.2 | -0.5 | 1366 | -6.6 | -5.3 | -1.3 |
| 202 | -7.7 | -5.7 | -2 | 1417 | -6.6 | -5.1 | -1.5 |
| 208 | -7.7 | -6.1 | -1.6 | 5 | -6.5 | -7.9 | 1.4 |
| 229 | -7.7 | -6.2 | -1.5 | 14 | -6.5 | -5.4 | -1.1 |
| 250 | -7.7 | -6.2 | -1.5 | 137 | -6.5 | -7.9 | 1.4 |
| 322 | -7.7 | -7.4 | -0.3 | 143 | -6.5 | -5.7 | -0.8 |
| 418 | -7.7 | -7.2 | -0.5 | 179 | -6.5 | -5.9 | -0.6 |
| 601 | -7.7 | -6.8 | -0.9 | 205 | -6.5 | -6.1 | -0.4 |
| 1205 | -7.7 | -5.8 | -1.9 | 347 | -6.5 | -5.9 | -0.6 |
| 1273 | -7.7 | -7 | -0.7 | 349 | -6.5 | -7 | 0.5 |
| 1346 | -7.7 | -5.4 | -2.3 | 401 | -6.5 | -5.4 | -1.1 |
| 1369 | -7.7 | -7 | -0.7 | 439 | -6.5 | -7 | 0.5 |
| 1370 | -7.7 | -7 | -0.7 | 445 | -6.5 | -7.1 | 0.6 |
| 134 | -7.6 | -5.4 | -2.2 | 457 | -6.5 | -7 | 0.5 |
| 169 | -7.6 | -5.4 | -2.2 | 514 | -6.5 | -7.2 | 0.7 |
| 271 | -7.6 | -7.4 | -0.2 | 523 | -6.5 | -5.1 | -1.4 |
| 313 | -7.6 | -7 | -0.6 | 562 | -6.5 | -5.4 | -1.1 |
| 409 | -7.6 | -7.2 | -0.4 | 644 | -6.5 | -5.7 | -0.8 |
| 443 | -7.6 | -6 | -1.6 | 1018 | -6.5 | -6 | -0.5 |
| 461 | -7.6 | -5.9 | -1.7 | 1025 | -6.5 | -5.6 | -0.9 |
| 467 | -7.6 | -5.9 | -1.7 | 1027 | -6.5 | -5.1 | -1.4 |
| 473 | -7.6 | -5.9 | -1.7 | 1054 | -6.5 | -5.4 | -1.1 |
| 505 | -7.6 | -7 | -0.6 | 1174 | -6.5 | -5.5 | -1 |
| 604 | -7.6 | -7.2 | -0.4 | 1216 | -6.5 | -7.2 | 0.7 |
| 610 | -7.6 | -7.2 | -0.4 | 1225 | -6.5 | -6.7 | 0.2 |
| 673 | -7.6 | -6.3 | -1.3 | 1249 | -6.5 | -7.2 | 0.7 |
| 674 | -7.6 | -6.1 | -1.5 | 1264 | -6.5 | -6.9 | 0.4 |
| 1139 | -7.6 | -6 | -1.6 | 1345 | -6.5 | -7 | 0.5 |
| 1204 | -7.6 | -5.2 | -2.4 | 1399 | -6.5 | -5.5 | -1 |
| 1211 | -7.6 | -5.9 | -1.7 | 1408 | -6.5 | -6.8 | 0.3 |
| 1300 | -7.6 | -6.9 | -0.7 | 104 | -6.4 | -5.9 | -0.5 |
| 1307 | -7.6 | -5.8 | -1.8 | 116 | -6.4 | -5.9 | -0.5 |
| 55 | -7.5 | -7.1 | -0.4 | 167 | -6.4 | -5.9 | -0.5 |
| 64 | -7.5 | -7 | -0.5 | 191 | -6.4 | -6 | -0.4 |
| 146 | -7.5 | -5.5 | -2 | 295 | -6.4 | -6.8 | 0.4 |
| 248 | -7.5 | -5.9 | -1.6 | 355 | -6.4 | -6.6 | 0.2 |
| 316 | -7.5 | -6.9 | -0.6 | 358 | -6.4 | -5.4 | -1 |
| 326 | -7.5 | -5.4 | -2.1 | 379 | -6.4 | -5.2 | -1.2 |
| 338 | -7.5 | -5.7 | -1.8 | 391 | -6.4 | -7 | 0.6 |
| 341 | -7.5 | -5.6 | -1.9 | 403 | -6.4 | -5.2 | -1.2 |
| 350 | -7.5 | -5.9 | -1.6 | 404 | -6.4 | -5.7 | -0.7 |
| 365 | -7.5 | -5.6 | -1.9 | 538 | -6.4 | -6.9 | 0.5 |
| 386 | -7.5 | -6.1 | -1.4 | 550 | -6.4 | -5.7 | -0.7 |
| 440 | -7.5 | -5.9 | -1.6 | 554 | -6.4 | -5.6 | -0.8 |
| 464 | -7.5 | -5.9 | -1.6 | 574 | -6.4 | -5 | -1.4 |
| 508 | -7.5 | -6.9 | -0.6 | 623 | -6.4 | -5.8 | -0.6 |
| 1048 | -7.5 | -5.8 | -1.7 | 635 | -6.4 | -5.8 | -0.6 |
| 1081 | -7.5 | -6.9 | -0.6 | 658 | -6.4 | -6.7 | 0.3 |
| 1090 | -7.5 | -7.4 | -0.1 | 1001 | -6.4 | -5.7 | -0.7 |
| 1186 | -7.5 | -7.1 | -0.4 | 1004 | -6.4 | -5.9 | -0.5 |
| 1202 | -7.5 | -5.8 | -1.7 | 1009 | -6.4 | -5.8 | -0.6 |
| 1282 | -7.5 | -7.1 | -0.4 | 1016 | -6.4 | -5.8 | -0.6 |
| 1298 | -7.5 | -5.7 | -1.8 | 1019 | -6.4 | -5.7 | -0.7 |
| 1324 | -7.5 | -6.5 | -1 | 1028 | -6.4 | -5.8 | -0.6 |
| 1378 | -7.5 | -6.9 | -0.6 | 1043 | -6.4 | -5.9 | -0.5 |
| 1388 | -7.5 | -6 | -1.5 | 1052 | -6.4 | -5.6 | -0.8 |
| 1394 | -7.5 | -5.6 | -1.9 | 1061 | -6.4 | -5.8 | -0.6 |
| 1396 | -7.5 | -7.5 | 0 | 1147 | -6.4 | -5.3 | -1.1 |
| 1420 | -7.5 | -6.5 | -1 | 1265 | -6.4 | -5.5 | -0.9 |
| 49 | -7.4 | -5.5 | -1.9 | 1309 | -6.4 | -5.1 | -1.3 |
| 50 | -7.4 | -6.1 | -1.3 | 1318 | -6.4 | -5 | -1.4 |
| 109 | -7.4 | -5.9 | -1.5 | 1336 | -6.4 | -5.2 | -1.2 |
| 133 | -7.4 | -5.9 | -1.5 | 1360 | -6.4 | -5.4 | -1 |
| 145 | -7.4 | -6.2 | -1.2 | 1405 | -6.4 | -6.9 | 0.5 |
| 164 | -7.4 | -5.7 | -1.7 | 62 | -6.3 | -6 | -0.3 |
| 257 | -7.4 | -6 | -1.4 | 140 | -6.3 | -5.5 | -0.8 |
| 262 | -7.4 | -8 | 0.6 | 157 | -6.3 | -5.9 | -0.4 |
| 280 | -7.4 | -7.3 | -0.1 | 299 | -6.3 | -8.1 | 1.8 |
| 367 | -7.4 | -6.6 | -0.8 | 307 | -6.3 | -5.3 | -1 |
| 392 | -7.4 | -6.1 | -1.3 | 311 | -6.3 | -5.8 | -0.5 |
| 395 | -7.4 | -8.1 | 0.7 | 329 | -6.3 | -8.3 | 2 |
| 446 | -7.4 | -5.9 | -1.5 | 362 | -6.3 | -5.6 | -0.7 |
| 449 | -7.4 | -5.8 | -1.6 | 373 | -6.3 | -5.3 | -1 |
| 494 | -7.4 | -5.4 | -2 | 437 | -6.3 | -5.7 | -0.6 |
| 563 | -7.4 | -5.9 | -1.5 | 478 | -6.3 | -5 | -1.3 |
| 605 | -7.4 | -7.7 | 0.3 | 547 | -6.3 | -6.7 | 0.4 |
| 1012 | -7.4 | -7.1 | -0.3 | 568 | -6.3 | -6.5 | 0.2 |
| 1040 | -7.4 | -5.8 | -1.6 | 596 | -6.3 | -5.7 | -0.6 |
| 1130 | -7.4 | -6.2 | -1.2 | 614 | -6.3 | -5.4 | -0.9 |
| 1196 | -7.4 | -5.6 | -1.8 | 638 | -6.3 | -5.7 | -0.6 |
| 1228 | -7.4 | -6.8 | -0.6 | 664 | -6.3 | -6.4 | 0.1 |
| 1229 | -7.4 | -5.5 | -1.9 | 667 | -6.3 | -4.7 | -1.6 |
| 1322 | -7.4 | -5.4 | -2 | 1067 | -6.3 | -5.7 | -0.6 |
| 1327 | -7.4 | -6.9 | -0.5 | 1070 | -6.3 | -5.4 | -0.9 |
| 1348 | -7.4 | -7.3 | -0.1 | 1100 | -6.3 | -6.2 | -0.1 |
| 1358 | -7.4 | -5.1 | -2.3 | 1106 | -6.3 | -5.3 | -1 |
| 1426 | -7.4 | -5.2 | -2.2 | 1121 | -6.3 | -5.9 | -0.4 |
| 1433 | -7.4 | -5.6 | -1.8 | 1133 | -6.3 | -5.6 | -0.7 |
| 59 | -7.3 | -5.9 | -1.4 | 1159 | -6.3 | -5.2 | -1.1 |
| 107 | -7.3 | -5.9 | -1.4 | 1199 | -6.3 | -5.7 | -0.6 |
| 118 | -7.3 | -5.9 | -1.4 | 1219 | -6.3 | -6.8 | 0.5 |
| 142 | -7.3 | -5.8 | -1.5 | 1237 | -6.3 | -5.2 | -1.1 |
| 148 | -7.3 | -7.4 | 0.1 | 1240 | -6.3 | -5.2 | -1.1 |
| 199 | -7.3 | -5.6 | -1.7 | 1342 | -6.3 | -5 | -1.3 |
| 238 | -7.3 | -6.5 | -0.8 | 1391 | -6.3 | -5.5 | -0.8 |
| 253 | -7.3 | -6.3 | -1 | 1432 | -6.3 | -6.7 | 0.4 |
| 256 | -7.3 | -8 | 0.7 | 353 | -6.2 | -6 | -0.2 |
| 296 | -7.3 | -6.1 | -1.2 | 356 | -6.2 | -5.7 | -0.5 |
| 334 | -7.3 | -5.6 | -1.7 | 368 | -6.2 | -6.2 | 0 |
| 343 | -7.3 | -5.7 | -1.6 | 371 | -6.2 | -5.8 | -0.4 |
| 434 | -7.3 | -6.1 | -1.2 | 382 | -6.2 | -5.2 | -1 |
| 455 | -7.3 | -5.9 | -1.4 | 407 | -6.2 | -6.4 | 0.2 |
| 463 | -7.3 | -6.5 | -0.8 | 425 | -6.2 | -5.7 | -0.5 |
| 479 | -7.3 | -5.8 | -1.5 | 469 | -6.2 | -5.4 | -0.8 |
| 481 | -7.3 | -5.7 | -1.6 | 470 | -6.2 | -5.9 | -0.3 |
| 530 | -7.3 | -5.6 | -1.7 | 502 | -6.2 | -6.8 | 0.6 |
| 1003 | -7.3 | -5.8 | -1.5 | 532 | -6.2 | -5.8 | -0.4 |
| 1015 | -7.3 | -6 | -1.3 | 545 | -6.2 | -5.6 | -0.6 |
| 1037 | -7.3 | -5.8 | -1.5 | 571 | -6.2 | -6.1 | -0.1 |
| 1042 | -7.3 | -5.6 | -1.7 | 632 | -6.2 | -5.8 | -0.4 |
| 1057 | -7.3 | -7 | -0.3 | 661 | -6.2 | -4.9 | -1.3 |
| 1127 | -7.3 | -5.9 | -1.4 | 1118 | -6.2 | -5.4 | -0.8 |
| 1142 | -7.3 | -6.1 | -1.2 | 1136 | -6.2 | -6.2 | 0 |
| 1160 | -7.3 | -5.4 | -1.9 | 1213 | -6.2 | -5.1 | -1.1 |
| 1175 | -7.3 | -5.7 | -1.6 | 1246 | -6.2 | -5 | -1.2 |
| 1177 | -7.3 | -7 | -0.3 | 1270 | -6.2 | -6.7 | 0.5 |
| 1208 | -7.3 | -5.9 | -1.4 | 332 | -6.1 | -5.8 | -0.3 |
| 1235 | -7.3 | -5.5 | -1.8 | 383 | -6.1 | -6 | -0.1 |
| 1253 | -7.3 | -8 | 0.7 | 451 | -6.1 | -6.8 | 0.7 |
| 1304 | -7.3 | -5.6 | -1.7 | 475 | -6.1 | -5.2 | -0.9 |
| 1325 | -7.3 | -5.9 | -1.4 | 520 | -6.1 | -5.8 | -0.3 |
| 1352 | -7.3 | -5.7 | -1.6 | 541 | -6.1 | -6.7 | 0.6 |
| 1367 | -7.3 | -6 | -1.3 | 548 | -6.1 | -5.7 | -0.4 |
| 1421 | -7.3 | -5.7 | -1.6 | 565 | -6.1 | -6.7 | 0.6 |
| 1439 | -7.3 | -5.8 | -1.5 | 668 | -6.1 | -5.7 | -0.4 |
| 119 | -7.2 | -5.6 | -1.6 | 1195 | -6.1 | -5.1 | -1 |
| 160 | -7.2 | -5.8 | -1.4 | 1243 | -6.1 | -4.9 | -1.2 |
| 175 | -7.2 | -6.6 | -0.6 | 1291 | -6.1 | -6.4 | 0.3 |
| 289 | -7.2 | -6.5 | -0.7 | 1310 | -6.1 | -5.6 | -0.5 |
| 305 | -7.2 | -5.4 | -1.8 | 1315 | -6.1 | -5.3 | -0.8 |
| 359 | -7.2 | -5.8 | -1.4 | 1409 | -6.1 | -5.5 | -0.6 |
| 374 | -7.2 | -5.6 | -1.6 | 1438 | -6.1 | -4.9 | -1.2 |
| 452 | -7.2 | -5.9 | -1.3 | 136 | -6 | -6 | 0 |
| 476 | -7.2 | -6.3 | -0.9 | 1241 | -6 | -5.8 | -0.2 |
| 496 | -7.2 | -6.9 | -0.3 | 1339 | -6 | -5.1 | -0.9 |
| 533 | -7.2 | -5.5 | -1.7 | 1024 | -5.9 | -7 | 1.1 |
| 539 | -7.2 | -5.8 | -1.4 | 1429 | -5.9 | -5.2 | -0.7 |
| 577 | -7.2 | -7.2 | 0 | 1412 | -5.8 | -5.8 | 0 |
| 652 | -7.2 | -6.9 | -0.3 | 1168 | -5.7 | -7 | 1.3 |
| 1013 | -7.2 | -5.8 | -1.4 | 1312 | -5.7 | -6.3 | 0.6 |
| 1030 | -7.2 | -6 | -1.2 | 1411 | -5.7 | -5.1 | -0.6 |
| 1031 | -7.2 | -5.6 | -1.6 | 637 | -5.6 | -7 | 1.4 |
| 1064 | -7.2 | -5.5 | -1.7 | 1334 | -7.1 | -5.2 | -1.9 |
| 1093 | -7.2 | -6 | -1.2 | 1382 | -7.1 | -5.3 | -1.8 |
| 1163 | -7.2 | -5.6 | -1.6 | 1424 | -7.1 | -5.4 | -1.7 |
| 1166 | -7.2 | -5.2 | -2 | 1430 | -7.1 | -5.8 | -1.3 |
| 1190 | -7.2 | -5.3 | -1.9 | 106 | -7 | -6.2 | -0.8 |
| 1214 | -7.2 | -5.6 | -1.6 | 154 | -7 | -6 | -1 |
| 1223 | -7.2 | -5.8 | -1.4 | 181 | -7 | -5.7 | -1.3 |
| 1271 | -7.2 | -6 | -1.2 | 184 | -7 | -5.4 | -1.6 |
| 1313 | -7.2 | -5.7 | -1.5 | 242 | -7 | -6.3 | -0.7 |
| 1328 | -7.2 | -5.6 | -1.6 | 266 | -7 | -6.2 | -0.8 |
| 1355 | -7.2 | -6 | -1.2 | 274 | -7 | -6.1 | -0.9 |
| 1364 | -7.2 | -5.5 | -1.7 | 277 | -7 | -6.1 | -0.9 |
| 1406 | -7.2 | -5.8 | -1.4 | 436 | -7 | -7.1 | 0.1 |
| 1415 | -7.2 | -5.8 | -1.4 | 557 | -7 | -5.6 | -1.4 |
| 58 | -7.1 | -5.5 | -1.6 | 566 | -7 | -5.5 | -1.5 |
| 112 | -7.1 | -7.1 | 0 | 626 | -7 | -5.7 | -1.3 |
| 241 | -7.1 | -6.2 | -0.9 | 1039 | -7 | -5.6 | -1.4 |
| 269 | -7.1 | -6.3 | -0.8 | 1108 | -7 | -7.4 | 0.4 |
| 377 | -7.1 | -5.9 | -1.2 | 1112 | -7 | -6.2 | -0.8 |
| 380 | -7.1 | -6.1 | -1 | 1132 | -7 | -5.3 | -1.7 |
| 487 | -7.1 | -5.3 | -1.8 | 1244 | -7 | -5.5 | -1.5 |
| 536 | -7.1 | -5.5 | -1.6 | 1145 | -7.1 | -5.6 | -1.5 |
| 551 | -7.1 | -5.8 | -1.3 | 1193 | -7.1 | -5.3 | -1.8 |
| 583 | -7.1 | -6.7 | -0.4 | 1217 | -7.1 | -5.8 | -1.3 |
| 586 | -7.1 | -6.8 | -0.3 | 1220 | -7.1 | -5.5 | -1.6 |
| 607 | -7.1 | -6.7 | -0.4 | 1222 | -7.1 | -5 | -2.1 |
| 1021 | -7.1 | -5.9 | -1.2 | 1232 | -7.1 | -6 | -1.1 |
| 1022 | -7.1 | -5.8 | -1.3 | 1238 | -7.1 | -5.9 | -1.2 |
| 1046 | -7.1 | -5.7 | -1.4 | 1289 | -7.1 | -7.7 | 0.6 |
| 1055 | -7.1 | -5.7 | -1.4 | 1301 | -7.1 | -5.8 | -1.3 |
| 1079 | -7.1 | -5.8 | -1.3 | 1303 | -7.1 | -5.4 | -1.7 |
| 1316 | -7.1 | -5.6 | -1.5 |  |  |  |  |
