# Supplementary material for: Novel inhibitors against Cryptosporidium parvum through blocking its energy metabolism
Source: PLoS Negl Trop Dis. 2026 Oct 5;20(10):e0014742. doi: 10.1371/journal.pntd.0014742 (PMC13652328; doi:10.1371/journal.pntd.0014742)
Supplement: Supplementary file 3 [file pntd.0014742.s002.docx]

**Supplementary Table 2** Mouse Intestinal Barrier and Inflammatory Gene Primers

| Target gene | Primer sequence（5′→3′） |
| --- | --- |
| ZO-1 | F-TGGTGCTCCTAAACAATC |
|  | R-TGCTATTACACGGTCCTC |
| Occludin | F-CCTGGAGGTACTGGTCT |
|  | R-ATCTTTCTTCGGGTTTT |
| Caspase-1 | F-ATTACAGACAAGGGTGCTGAACAA |
|  | R-CTTTCGGAATAACGGAGTCAATCA |
| β-actin | F-CCCATCTATGAGGGTTACGC |
|  | R-TTTAATGTCACGCACGATTTC |
