# Supplementary material for: Novel inhibitors against Cryptosporidium parvum through blocking its energy metabolism
Source: PLoS Negl Trop Dis. 2026 Oct 5;20(10):e0014742. doi: 10.1371/journal.pntd.0014742 (PMC13652328; doi:10.1371/journal.pntd.0014742)
Supplement: Supplementary file 4 [file pntd.0014742.s003.docx]

**Supplementary Table 3** Calf Intestinal Barrier and Inflammatory Gene Primers

| Target gene | Primer sequence（5′→3′） |
| --- | --- |
| ZO-1 | F- TCGTCTGTCCTACCTGTC |
|  | R- CTGTATCTGTGTCTTCATAGTC |
| Caspase-1 | F- CAGATGTGCTACCTTATGATAC |
|  | R- GCCTGGACAATGATGACTT |
| GAPDH | F- ACAGTCAAGGCAGAGAACGG |
|  | R- CTCGTGGTTCACACCCATCA |
| Claudin-1 | F- GACTCCTTGCTGAATCTGAA |
|  | R- CCATCCGCATCTTCTGTG |
| Occludin | F- CCGTATCCAGAGTCTTCCT |
|  | R- AGCGTCTCCAAGTTACCA |
