# Supplementary material for: Novel inhibitors against Cryptosporidium parvum through blocking its energy metabolism
Source: PLoS Negl Trop Dis. 2026 Oct 5;20(10):e0014742. doi: 10.1371/journal.pntd.0014742 (PMC13652328; doi:10.1371/journal.pntd.0014742)
Supplement: Supplementary file 6 [file pntd.0014742.s012.docx]

Response to Editor Comments

**1) We have noticed that you have uploaded " S1_raw_image" file as supporting information but you have not included it in the list of legends. Please add a citation of this SI file into the list of legends of your supporting information files.**

**Response:**

We apologize for this oversight. We have now added the citation of "S1_Raw_Images" to the Supporting Information legends with the following description: "Original uncropped images of Western blots and gel electrophoresis.".

**2) "Please ensure that the CRediT author contributions listed for every co-author are completed accurately and in full.**

**At this stage, the following Authors require contributions: [ALL AUTHORS]. Please ensure that the full contributions of each author are acknowledged in the ""Add/Edit/Remove Authors"" section of our submission form.**

**Response:**

We have now completed the CRediT author contributions for ALL authors in the submission form.

Response to Reviewers' Comments

Reviewer #2

We thank the reviewer for recognizing the improvements made in our first revision and for providing these constructive suggestions. We fully acknowledge the importance of both the quantitative enzyme inhibition data and the complete compound characterization for definitive target validation. We address each point in detail below.

**1) Quantitative enzyme inhibition data (IC₅₀ values) against both CpLDH and hLDH should be performed to support the proposed mechanism and assess target selectivity, building directly on the recombinant enzyme assays already reported in the manuscript.**

**Response:**

We thank the reviewer for this important suggestion. We agree that determining the enzyme-level IC₅₀ values against recombinant CpLDH and human LDH isoforms would represent the definitive approach to confirm target engagement and selectivity. However, we respectfully note that these experiments are beyond the scope of the current revision due to substantial technical requirements, including re-expression and large-scale purification of recombinant CpLDH protein, acquisition of human LDH isoforms, optimization of enzyme kinetics assay conditions, and systematic dose-response measurements across multiple concentrations, which would require several months to complete.

In the current study, we have provided the following evidence supporting target engagement and selectivity:

(1) Single-concentration enzyme inhibition screening (Fig. 2b): Among the 29 synthesized compounds, 14 exhibited significantly higher inhibition rates against recombinant CpLDH than NSC158011 at 2 μM, confirming direct interaction with the enzyme.

(2) Cellular anti-*C. parvum* IC₅₀ values (Fig. 2c): Dose-response curves and IC₅₀ values (H25: 7.44 μM; H29: 8.42 μM) demonstrate potent antiparasitic efficacy.

(3) Selectivity indices (Table 1): Both compounds showed favorable selectivity (SI > 10), with cytotoxicity CC₅₀ against host cells substantially higher than their antiparasitic IC₅₀ values (H25: CC₅₀ = 282.11 μM; H29: CC₅₀ = 140.20 μM).

(4) Molecular docking (Fig. 1a, 1b): H25 and H29 showed stronger binding to CpLDH (-6.56 and -5.83 kcal/mol) compared to human LDH (-4.29 and -3.99 kcal/mol), supporting selectivity at the structural level.

(5) Metabolomics evidence: Restoration of TCA cycle intermediates following treatment is consistent with the expected metabolic consequences of LDH inhibition.

We have added a clearer distinction in the revised manuscript between "evidence supporting target association" (the data we provide) and "evidence required for definitive target validation" (which we acknowledge requires further study). Specifically, we have revised the Discussion to state: "The data presented here provide strong correlative evidence for CpLDH as a relevant target, with consistent results across enzymatic, cellular, and animal models. However, we acknowledge that definitive validation of CpLDH as the direct molecular target requires quantitative enzyme IC₅₀ determination against both CpLDH and human LDH isoforms, as well as direct target engagement assays. These experiments are planned for our follow-up studies."

We hope the reviewer appreciates that while we cannot complete these experiments within the current revision timeframe, we have clearly acknowledged this as a limitation and have committed to completing these studies in the future.

**2) Second, the chemical characterization remains incomplete. Partial characterization data were provided only for NSC158011, H25, and H29, whereas the remaining synthesized compounds were not comprehensively characterized. For a medicinal chemistry study, appropriate structural confirmation using techniques such as 1H NMR, 13C NMR, HRMS, and purity assessment should be provided for all synthesized compounds.**

**Response:**

We thank the reviewer for this comment. We would like to clarify the characterization status of the compounds in our study.

As stated in the Materials and Methods section, only the three lead compounds (NSC158011, H25, and H29, which demonstrated the most promising biological activity and were selected for in vivo studies, were fully characterized by ¹H NMR and HPLC purity determination (>98%). The remaining 26 compounds were synthesized for the purpose of initial activity screening (enzymatic inhibition and cytotoxicity assessment). After the screening assays, these compounds showed limited or no activity and were therefore not pursued further. We did not claim in the manuscript that all compounds were fully characterized, and we have been transparent that only the leads received complete characterization.

We also wish to be transparent that our research funding has been fully expended and the project has concluded, which precludes us from performing additional analytical characterization at this time. Given that these 26 compounds were inactive in the screening assays and are not the focus of this study, we respectfully believe that their limited characterization is acceptable for their role in the preliminary screening.

In the revised manuscript, we have added a limitation statement in the Discussion acknowledging this and committing to complete characterization of all compounds in future studies if they become relevant for further development. We trust that the reviewer appreciates our honest acknowledgment of this limitation.

**3) If additional biochemical studies are beyond the scope of the current revision, the manuscript would benefit from a clearer distinction between evidence supporting target association and evidence required for definitive target validation.**

**Response:**

We thank the reviewer for this helpful suggestion. In the revised Discussion section, we have now clearly distinguished between these two levels of evidence. Specifically, we have added the following statement at the end of the limitations paragraph:

"Despite these limitations, the consistent results from enzymatic screening, cellular efficacy, animal models, and metabolomics analyses collectively support CpLDH as a relevant target for the anti-C. parvum activity of H25 and H29, although we acknowledge that the data presented here represent supportive evidence for target association rather than definitive target validation."

This wording makes explicit that our data provide supportive evidence for target association, while definitive target validation (quantitative enzyme IC₅₀, selectivity against human LDH isoforms, and direct target engagement assays) will require future studies. We believe this addresses the reviewer's concern.
