# Supplementary material for: Novel inhibitors against Cryptosporidium parvum through blocking its energy metabolism
Source: PLoS Negl Trop Dis. 2026 Oct 5;20(10):e0014742. doi: 10.1371/journal.pntd.0014742 (PMC13652328; doi:10.1371/journal.pntd.0014742)
Supplement: Supplementary file 7 [file pntd.0014742.s011.docx]

Response to Editor and Reviewer comments

1. **Please ensure that the CRediT author contributions listed for every co-author are completed accurately and in full. At this stage, the following Authors/Authors require contributions: Chang Xu, Yaru Ji, Qiang Sha, and Kun Li.**

**Response:**

We greatly appreciate your suggestion. We have completed the CRediT author contributions for all co-authors in the submission system. The specific contributions are as follows:” Chang Xu: Conceptualization, Methodology, Investigation, Visualization, Writing – original draft, Writing – review & editing. Yaru Ji: Investigation, Formal analysis, Data curation. Qiang Sha: Resources, Formal analysis. Kun Li: Conceptualization, Methodology, Funding acquisition, Project administration, Supervision. All authors have read and approved the final manuscript.” We have highlighted the revised content in the document.

2. **We do not publish any copyright or trademark symbols that usually accompany proprietary names, eg ©, ®, or TM (e.g. next to drug or reagent names). Therefore please remove all instances of trademark/copyright symbols throughout the text, including:**

**- ® on pages: 12, and 13**

**- TM on page: 14.**

**Response:**

We have carefully reviewed the entire manuscript and removed all trademark/copyright symbols (® and TM), including the ® symbols on pages 12 and 13 and the TM symbol on page 14.

3. **Please amend your detailed Financial Disclosure statement. This is published with the article. It must therefore be completed in full sentences and contain the exact wording you wish to be published. (i) State the initials, alongside each funding source, of each author to receive each grant. (ii) State what role the funders took in the study. (iii) If any authors received a salary from any of your funders, please state which authors and which funders.**

**Response:**

We have revised the Financial Disclosure statement as follows: This work was supported by the National Natural Science Foundation of China (Grant No. 32102692 to K.L.). The funders had no role in study design, data collection and analysis, decision to publish, or preparation of the manuscript. No authors received salaries from the funders.

4. **Thank you for stating " All raw sequence data from animals were deposited in the NCBI Sequence Read Archive database under accession number: PRJNA1293894, PRJNA1293901 and PRJNA1294146". However we are unable to access the Accession number from the information you provided. Please note that, though access restrictions are acceptable now, your entire data will need to be made freely accessible if your manuscript is accepted for publication.Please review and update the Data Availability Statement to ensure that the underlying data are publicly accessible and that all accession numbers, links, or repository details are correct and functional.**

**Response:**

We have checked and confirmed three accession numbers in the NCBI SRA database (PRJNA1293894, PRJNA1293901, PRJNA1294146). Previously, for privacy reasons, we chose to release them after publication. We have updated the release dates and now all three accession numbers can be searched for.

5. **We have amended your Competing Interest statement to comply with journal style. We kindly ask that you double check the statement and let us know if anything is incorrect.**

**Response:**

We have reviewed the Competing Interest statement and confirm that it is correct. The authors declare no competing interests.

6. **PLOS now requires that authors provide the original uncropped and unadjusted images underlying all blot or gel results reported in a submission’s figures or Supporting Information files. This policy and the journal’s other requirements for blot/gel reporting and figure preparation are described in detail at https://journals.plos.org/plosone/s/figures#loc-blot-and-gel-reporting-requirements and <https://journals.plos.org/plosone/s/figures#loc-preparing-figures-from-image-files>.**

**Response:**

We have compiled all original uncropped and unadjusted images underlying the Western blot and gel electrophoresis results into a single PDF file (named “S1_raw_images”), which will be submitted as Supporting Information with the revised manuscript. All original images have been annotated in accordance with the journal’s requirements.

Response to Reviewers' Comments

Reviewer #1

1. **Please ensure that the computational workflow is fully reproducible. Critical methodological details are currently missing, including the docking algorithm, grid dimensions, exhaustiveness parameters, validation procedures, and receptor preparation methods.**

**Response:**

Thank you for the valuable comments from the reviewers. We have supplemented the detailed parameters of the molecular docking in the "Materials and Methods" section of the revised manuscript. The specific additions are as follows: Molecular docking simulations were conducted using AutoDockTools Vina, the crystal structures of CpLDH (PDB: 4ND5) and H-LDH (PDB: 1I0Z) were obtained from the RCSB PDB database; the receptor protein was preprocessed through AutoDockTools with hydrogenation, calculation of Gasteiger charges, and addition of non-polar hydrogen, etc. The drug components were subjected to energy minimization using ChemDraw 3D. The number of connection operations was 15 times. Use pymol for visualization of docking.

2. **The methodological sequence is unclear. The manuscript initially refers to “synthesized derivatives,” but later states that the compounds will be synthesized after the docking analysis. It is therefore unclear how many derivatives were designed, how many were synthesized, which compounds passed the docking screening, and which were subjected to biological evaluation. This represents a major concern in medicinal chemistry studies and should be clarified explicitly.**

**Response:**

We thank the reviewer for pointing out this issue. We have reorganized the methodological workflow in the revised manuscript to make it clearer. The specific workflow is as follows: (1) Based on the structure of NSC158011, a virtual library of 1,440 derivatives was constructed through computer-aided drug design; (2) Through molecular docking screening, 722 candidate compounds with high docking scores against CpLDH were selected; (3) Twenty-nine representative derivatives were selected for actual chemical synthesis; (4) Enzymatic inhibition screening of the 29 synthesized compounds identified 14 compounds with good in vitro inhibitory activity; (5) Cytotoxicity evaluation of the 14 compounds identified 8 compounds with relatively low cytotoxicity; (6) Through in vitro anti-parasitic assays, H25 and H29 were identified as the two optimal compounds for further in-depth study. We have clearly distinguished the two stages of “virtual design/in silico screening” and “actual chemical synthesis/biological evaluation” in the revised manuscript and have supplemented the compound numbers at each stage.

3. **The statistical analysis section is unclear. Please clearly indicate the statistical test applied and justify its appropriateness for the data. The section related to microbiota, metabolomics, correlations, and differential abundance analyses lacks sufficient statistical detail. There is no description of the statistical methods used, including FDR correction methods, Benjamini–Hochberg adjustment, or q-values. Similarly, for the correlation analyses, it is unclear whether Pearson or Spearman correlations were applied, and whether multiple testing correction was performed. Please ensure that all statistical approaches are comprehensively described in the data analysis section.**

**Response:**

We thank the reviewer for this suggestion. We have substantially expanded the “Statistical Analysis” section in the revised manuscript to provide detailed descriptions of all statistical methods: (1) Unpaired two-tailed Student’s t-test was used for comparisons between two groups, and one-way ANOVA followed by Tukey’s post-hoc test was used for comparisons among multiple groups, (2) Differential abundance analysis of microbiota was performed using DESeq2 (negative binomial distribution model) and LEfSe (Linear Discriminant Analysis Effect Size), (3) Differential metabolite screening in metabolomics was performed using OPLS-DA models (VIP > 1.0) combined with univariate analysis (fold change > 1.5 or < 0.67, P < 0.05), (4) For correlation analyses, Spearman’s rank correlation (for non-normally distributed data) or Pearson correlation (for normally distributed data) was applied based on data distribution, with Benjamini-Hochberg multiple testing correction.

4. **Please ensure that all synthesized compounds are adequately characterized. The manuscript should explicitly state whether characterization was performed using ^1H NMR, ^13C NMR, and high-resolution mass spectrometry (HRMS). In addition, please report compound purity values**

**Response:**

We thank the reviewer for this important suggestion. We acknowledge that full characterization was performed only for the three key compounds (NSC158011, H25, and H29) that demonstrated the most promising biological activity and were selected for further in vivo studies, as these were the primary focus of our current investigation.

For these three compounds, the following characterization data are available:

All three compounds were characterized by ¹H NMR spectroscopy (Bruker Avance III 400 MHz), and the spectra have been provided in the revised manuscript.Compound purities were determined and all three compounds showed purities > 98%.

For the remaining 26 compounds, they were synthesized as part of the preliminary screening campaign and were evaluated only for enzymatic inhibition and cytotoxicity, without full NMR characterization. We hope the reviewer appreciates that the compounds with complete characterization and purity data are those that represent the main findings of this study.

5. **It is unclear whether the study included 29 or 30 compounds/components. Please revise the text to ensure consistency throughout the manuscript.**

**Response:**

We thank the reviewer for pointing out this inconsistency. Upon verification, a total of 29 NSC158011 derivatives were designed, synthesized, and biologically evaluated in this study (excluding the parent compound NSC158011 itself). If the parent compound NSC158011 is included, the total is 30 compounds. We have standardized the wording throughout the revised manuscript to clearly distinguish between “29 derivatives” and “30 compounds including the parent compound.”

6. **There is ambiguity regarding the interpretation of the docking results. It is unclear whether the manuscript refers to higher/lower docking scores or binding energy/affinity values. Please define the scoring system clearly and ensure consistent terminology throughout the manuscript.**

**Response:**

We thank the reviewer for this suggestion. We have clearly defined the scoring system in the revised manuscript: binding affinity was calculated using the AutoDock semi-empirical free energy function and is expressed in kcal/mol; lower values (more negative) indicate stronger binding affinity. We have uniformly used the terms “binding affinity” and “kcal/mol” throughout the manuscript and avoided the ambiguous term “docking score.”

7. **“*C. parvum*” is written inconsistently throughout the text. Please standardize the nomenclature according to journal style guidelines.**

**Response:**

We thank the reviewer for pointing out this issue. We have carefully reviewed the entire manuscript and standardized the writing of “C. parvum” to italicized “*C. parvum*” (genus abbreviation capitalized, species name in lowercase, entire name in italics) throughout the text, in accordance with the journal’s formatting requirements.

8. **Lines 545–546: “Cryptosporidium-induced intestinal barrier damage and systemic inflammatory responses.” The statement currently implies causality; however, the analyses presented do not appear sufficient to establish a direct causal relationship. Please revise the wording to avoid overinterpretation**

**Response:**

We thank the reviewer for this careful suggestion. We agree with this point and have revised the relevant statement to a more cautious wording: “... which may contribute to the improvement of intestinal barrier damage and systemic inflammatory responses associated with *C. parvum* infection.,” to reflect observed associations rather than direct causal relationships.

9. **The conclusion stating that “the two derivatives identified in this study holding significant implications for livestock production and public health” appears overstated considering the methodology and analyses presented. The workflow is difficult to follow, several analytical steps are insufficiently described, and the statistical power supporting these conclusions is unclear.**

**Response:**

We thank the reviewer for this suggestion. We agree with this point and have revised the conclusion to read: “The two derivatives identified in this study represent promising lead compounds for further development as anti-cryptosporidiosis agents, with potential relevance to livestock production and public health.” We have also noted that these findings still require further preclinical and clinical studies to validate their practical application value.

Reviewer #2

1. **While the manuscript demonstrates that three compounds inhibit recombinant CpLDH, it lacks quantitative enzymatic inhibition data. The authors should determine and report the IC₅₀ values of NSC158011, H25, and H29 against recombinant CpLDH, along with complete dose-response curves. Additionally, enzyme kinetic analyses should be conducted to define the mode of inhibition (competitive, noncompetitive, or mixed).**

**Response:**

We thank the reviewer for this important suggestion. We fully agree that determining the enzyme-level IC₅₀ values and inhibition kinetics of these compounds against recombinant CpLDH would substantially strengthen the mechanistic characterization of our lead compounds. However, we respectfully note that the timeframe for this revision does not allow us to complete these experiments, as they require re-expression and large-scale purification of recombinant CpLDH protein, optimization of assay conditions, and systematic enzyme kinetic analyses across multiple substrate concentrations, which typically takes several months. We plan to complete these experiments in our follow-up studies.

Nevertheless, we would like to emphasize the evidence we have already provided that strongly supports the CpLDH-targeting activity of our compounds:

(1) Single-concentration enzyme inhibition screening (Fig. 2b): Among the 29 synthesized compounds, 14 exhibited significantly higher inhibition rates against recombinant CpLDH than NSC158011 at 2 μM, confirming their direct interaction with the enzyme.

(2) Cellular anti-C. parvum IC₅₀ values (Fig. 2c): We have reported the dose-response curves and IC₅₀ values of H25 and H29 against C. parvum proliferation in HCT-8 cells (H25: 7.44 μM; H29: 8.42 μM), demonstrating their potent antiparasitic efficacy.

(3) Selectivity indices (Table 1): Both compounds showed favorable selectivity, with much higher cytotoxicity IC₅₀ values against host cells (H25: 282.11 μM; H29: 140.20 μM) compared to their antiparasitic IC₅₀ values.

(4) Molecular docking analysis (Fig. 1a): H25 and H29 showed strong binding interactions with key residues in the CpLDH active site (H25: -6.56 kcal/mol; H29: -5.83 kcal/mol), with weaker binding to human LDH, supporting target specificity.

We have now added a clear limitation statement in the Discussion section acknowledging that enzyme-level IC₅₀ determination and kinetic inhibition mode analysis have not been performed in this study, and we explicitly state that these experiments will be completed in our future work. We believe that the combination of enzymatic screening, cellular efficacy, selectivity data, docking analysis, and metabolomics evidence provides a robust characterization of the compounds’ mechanism of action, while we commit to providing the full enzyme kinetic data in subsequent studies.

2. **The manuscript states that eight compounds exhibited strong inhibitory activity and that fourteen showed favorable properties; however, the complete quantitative screening dataset is not presented. To improve transparency and enable a comprehensive assessment of the screening campaign, the authors should provide the full dataset, including activity measurements and relevant parameters for all evaluated compounds.**

**Response:**

We thank the reviewer for this suggestion. We have now compiled the complete screening dataset for all 29 synthesized compounds and provided it as Table 1 in the revised submission. This table includes: Compound identifiers, CpLDH inhibition activity (% inhibition at 2 μM), Cytotoxicity CC₅₀ values against HCT-8 cells (μM)

The anti-*C. parvum* IC₅₀ values are reported only for the three compounds (NSC158011, H25, and H29) for which complete dose-response curves were obtained (Fig. 2c). For the remaining compounds, IC₅₀ values could not be reliably determined due to insufficient activity in the preliminary screening.

3. **The manuscript proposes that the lead compounds exert their antiparasitic effects through CpLDH inhibition. However, evidence for direct target engagement is currently limited to molecular docking and in vitro enzyme inhibition assays. To strengthen this mechanistic claim, the authors should perform cellular target engagement via assays such as CETSA (Cellular Thermal Shift Assays) or NanoBRET (Nano-Bioluminescence Resonance Energy Transfer).**

**Response:**

We are grateful for the important suggestions provided by the reviewers. We fully agree that direct target binding experiments (such as CETSA or NanoBRET) will significantly enhance the persuasiveness of the mechanism study. We need to truthfully explain to the reviewers that these experiments require the establishment of a C. parvum infection cell model, optimization of CETSA experimental conditions (including temperature gradients, lysis conditions, antibody selection, etc.) or the establishment of a NanoBRET system (requiring the construction of a fusion expression vector), and these tasks cannot be completed within the current revision time frame. We have clearly pointed out this limitation in the "Discussion" section of the revised manuscript and stated that these experiments will be carried out in subsequent studies. Currently, our mechanism evidence chain includes: (1) Molecular docking shows the specific interaction of the compound with the active site of CpLDH (2) Recombinant CpLDH enzyme activity inhibition experiments (3) CpLDH is a key enzyme in *C. parvum*'s energy metabolism (supported by existing literature) (4) Metabolomics data show that the compound treatment restored the levels of TCA cycle-related metabolites. These pieces of evidence collectively support the rationality of CpLDH as the compound's target.

4. **Computational docking of the lead compounds to hLDH is insufficient to demonstrate selectivity toward CpLDH. The authors should biochemically assess the lead compounds against at least one recombinant human LDH isoform and report selectivity indices.**

**Response:**

We thank the reviewer for this suggestion. We agree that biochemical experiments are needed to verify the selectivity of the compounds for CpLDH over human LDH. However, we must honestly state that within the current revision timeframe, we are unable to complete the expression, purification, and enzyme inhibition assays for recombinant human LDH. In the original manuscript, we have already provided: (1) molecular docking showing significantly lower binding affinities of the compounds to hLDH compared to CpLDH (NSC158011: -5.55 vs -4.34 kcal/mol; H25: -6.56 vs -4.29 kcal/mol; H29: -5.83 vs -3.99 kcal/mol); (2) cytotoxicity data showing that the IC₅₀ values of the compounds against host HCT-8 cells are much higher than their anti-parasitic IC₅₀ values (e.g., H25: CC₅₀ = 282.11 μM vs IC₅₀ = 7.44 μM), suggesting good selectivity. We have explicitly acknowledged this limitation in the “Discussion” section of the revised manuscript and will supplement the enzyme inhibition data against human LDH isoforms in future studies.

5. **The manuscript repeatedly attributes the observed changes in the microbiome, metabolome, inflammatory responses, and intestinal barrier function to CpLDH inhibition. However, the current data do not establish a causal link between these effects and direct CpLDH inhibition, as they could simply result from reduced parasite burden following treatment. The authors should either provide additional experimental evidence supporting this mechanistic connection or substantially moderate these claims throughout the manuscript.**

**Response:**

We thank the reviewer for this important reminder. We fully agree with the reviewer’s point—the current data indeed cannot directly establish a causal chain from CpLDH inhibition to changes in the microbiome/metabolome/inflammation/barrier function, and these observed changes are likely largely indirect consequences of reduced parasite burden following treatment. We have comprehensively revised the relevant statements throughout the manuscript, changing causal language to descriptive/associative language. For example, “regulating gut microbiota through CpLDH inhibition” has been changed to “compound treatment was associated with restoration of gut microbiota, which may be related to reduced parasite burden”; “improving intestinal barrier by blocking energy metabolism” has been changed to “improvement of intestinal barrier function was observed following compound treatment, which may be partially attributed to reduced parasite load.” We have also added a dedicated limitations paragraph in the “Discussion” section, clearly stating that we cannot distinguish between direct target effects and indirect effects (via reduced parasite burden).

6. **The medicinal chemistry strategy is insufficiently described. The authors should provide a clearer explanation of how the 1,440 NSC158011 analogs were generated (including the computational tools, if applicable), as well as the rationale for their prioritization and selection.**

**Response:**

We thank the reviewer for this suggestion. We have supplemented the “Materials and Methods—Structure Optimization and Molecular Docking Simulation Analysis” section with detailed descriptions: Based on the core scaffold of NSC158011, systematic structural modifications were performed through the following strategies: (1) introduction of electron-donating or electron-withdrawing substituents (such as -OCH₃, -Cl, -F, -CF₃, etc.) at different positions of the phenyl and naphthyl rings; (2) oxidation of the thioether bond to sulfoxide or sulfone; (3) introduction of different alkyl or aryl groups at the amide/thioamide position. All virtual derivatives were generated and optimized using ChemDraw and SYBYL software packages. The prioritization criteria were: (1) docking binding affinity to CpLDH ≥ NSC158011; (2) docking binding affinity to hLDH ≥ -4.50 kcal/mol (to ensure some degree of selectivity).

7. **The authors should report the selectivity index (SI = CC₅₀/IC₅₀) for all tested compounds mentioned in table 1 and clearly define the criteria used to classify compounds as selective. For reference, compounds with an SI > 10 are often considered to have acceptable selectivity.**

**Response:**

We thank the reviewer for this suggestion. We have now calculated the selectivity index (SI = CC₅₀/IC₅₀) for all compounds for which both cytotoxicity (CC₅₀) and anti-C. parvum IC₅₀ values were available. For the three compounds with complete dose-response data (NSC158011, H25, and H29), the SI values are 11.56, 37.92, and 16.65, respectively. Following the established criterion (SI > 10 indicates acceptable selectivity), both H25 and H29 demonstrate favorable selectivity. These data have been added to Table 1 in the revised manuscript. For the other compounds listed in Table 1, SI values could not be calculated because they did not exhibit sufficient anti-C. parvum activity in preliminary screening to generate reliable dose-response curves. We have added a footnote to Table 1 to clarify this. Additionally, we have explicitly defined the SI > 10 criterion in the Results section.

8. **CpLDH is a well-established drug target for cryptosporidiosis, and numerous CpLDH inhibitors have been reported. The authors should expand the Introduction to include key contributions from other groups and better contextualize the novelty of their work within the existing literature.**

**Response:**

We thank the reviewer for this suggestion. We have expanded the literature review in the Introduction section, adding the following: (1) the biochemical and structural characterization of CpLDH by Cook et al. (2015, Int J Biol Macromol); (2) the study by Zhang et al. (2015, PLoS Pathog) on the association of CpLDH with the parasitophorous vacuole membrane and its potential as a drug target; (3) progress on small-molecule CpLDH inhibitors reported by other groups. By supplementing these references, we have better contextualized the novelty of our study within the existing literature: the novelty of this study lies in the systematic structure optimization and comprehensive in vivo and in vitro evaluation (including two animal models and multi-omics analyses), reporting for the first time novel CpLDH inhibitor derivatives with good in vivo activity and safety profiles.

9. Compound nomenclature should be standardized throughout the manuscript. Inconsistent use of identifiers (e.g., H25 vs. compound 25 and H29 vs. compound 29) may cause confusion and should be corrected for clarity.

**Response:**

We thank the reviewer for pointing out this issue. We have carefully reviewed the entire manuscript and standardized the compound nomenclature to “H25” and “H29” (consistent with Figure 1 and throughout the text), and no longer alternately use “compound 25/29.” The parent compound is consistently referred to as “NSC158011.”

10. **The manuscript contains numerous grammatical, typographical, and scientific inaccuracies and would benefit from careful English-language editing. Examples include:**

**(i) Page 3, lines 63–66: The statements “Currently, there are no vaccines or therapeutic drugs specifically targeting cryptosporidiosis” and “Nitazoxanide is the sole FDA-approved drug for treating cryptosporidiosis” are contradictory and should be revised for clarity and accuracy.**

**Response:**

We have revised this to: “Currently, there are no vaccines available for cryptosporidiosis” This clearly conveys both “no vaccines” and “only one therapeutic drug,” avoiding contradiction.

**(ii) Page 4, lines 102–104: The authors state that “LDH serves as a drug target for Plasmodium, Toxoplasma gondii, Giardia lamblia, Trypanosoma cruzi, and Entamoeba histolytica within the Apicomplexa phylum.” This is incorrect, as Entamoeba histolytica is not an apicomplexan parasite.**

**Response:**

We thank the reviewer for pointing out this scientific error. We have revised this to: “LDH serves as a drug target for various parasitic protozoa, including *Plasmodium, Toxoplasma gondii, Giardia lamblia, and Trypanosoma cruzi*, as well as *Entamoeba histolytica.*”

**(iii) Page 5, line 129: The term “simulated chemical synthesis” is unclear and should be defined or replaced with more appropriate terminology.**

**Response:**

We have provided a more detailed description of the steps of computer-aided optimization, which more accurately reflects the essence of computer-aided drug design.

**(iv) Page 16, lines 438–439: Replace “The active sites of the compounds and protein crystal structures were evaluated using scoring functions” with “The binding affinities of the compounds for the CpLDH active site were evaluated using scoring functions.”**

**Response:**

We have made the revision as suggested.

**(v) Page 2:**

**Lines 49–50: Replace “public health officials in 40 states and Puerto Rico have voluntarily annunciated” with “U.S. public health officials in 40 states and Puerto Rico have voluntarily announced.”**

**Line 53: Replace “Mortality rates can reach 10%-20%” with “Mortality rates can reach 10–20%.”**

**Line 57: Replace “Cryptosporidium-induced cryptosporidiosis” with “Cryptosporidiosis.”**

**Response:**

Thank you very much for your careful suggestions. We have made all the revisions.

**(vi) Page 3, lines 81–82: Avoid unnecessary capitalization of protein names throughout the manuscript (e.g., zonula occludens-1, claudins, and occludin).**

**Response:**

We have reviewed the entire manuscript and standardized protein names to lowercase (unless proper nouns or at the beginning of sentences), including zonula occludens-1, claudins, occludin, etc.

**(vii) Page 4:**

**Line 94: Replace “Unlike other protozoa” with “Unlike other Apicomplexa.”**

**Line 96: Replace “does not possess apicoplast organelles” with “does not possess an apicoplast.”**

**Line 109: Replace “lactate dehydrogenase gene” with “LDH,” as the abbreviation has already been introduced.**

**Response:**

Thank you very much for your careful suggestions. We have made all the revisions.

**(viii) Page 8, line 222: Replace “An enzyme activity detection system” with “An enzymatic assay.”**

**Response:**

Thank you very much for your careful suggestions. We have made all the revisions.

Reviewer #3

1. **For the title, choose between “anti-” and “against.”**

**Response:**

We thank the reviewer for this suggestion. We have revised the title to: “Novel inhibitors against *Cryptosporidium parvum* through blocking its energy metabolism,” using “against” instead of “anti-against.”

2. **Line 148: Scheme 1 is missing from the manuscript.**

**Response:**

We apologize for the confusion. Scheme 1 (the three synthetic routes A, B, and C) was actually presented as Fig. 1c in the original submission, but we failed to clearly state this in the text. To avoid ambiguity, we have now added an explicit citation at line 148: "as shown in Fig. 1c." We hope this clarification resolves the issue.

3. **Include abbreviations used in the figures in the figure legends as well.**

**Response:**

We thank the reviewer for this suggestion. We have reviewed all figure legends and ensured that all abbreviations are fully defined in the corresponding legends (e.g., CpLDH, H-LDH, ZO-1, TJ, etc.).

4. Check spelling throughout the text.

**Response:**

We thank the reviewer for this suggestion. We have conducted a thorough spell-check and grammar check of the entire manuscript using professional tools, and have had the manuscript proofread by a native English-speaking colleague. All identified spelling errors have been corrected.
