# Supplementary material for: Novel inhibitors against Cryptosporidium parvum through blocking its energy metabolism
Source: PLoS Negl Trop Dis. 2026 Oct 5;20(10):e0014742. doi: 10.1371/journal.pntd.0014742 (PMC13652328; doi:10.1371/journal.pntd.0014742)
Supplement: Peer review history

# Peer review: Decision Letter 0

**DOI:** 10.1371/journal.pntd.0014742.r001

**Authors:** Guilherme Werneck

11 Jul 2026

PNTD-D-26-00386

Novel inhibitors anti-against Cryptosporidium parvum through blocking its energy metabolism

PLOS Neglected Tropical Diseases

Dear Dr. Li,

Thank you for submitting your manuscript to PLOS Neglected Tropical Diseases. After careful consideration, we feel that it has merit but does not fully meet PLOS Neglected Tropical Diseases's publication criteria as it currently stands. Therefore, we invite you to submit a revised version of the manuscript that addresses the points raised during the review process.

Please submit your revised manuscript within by Sep 09 2026 11:59PM. If you will need more time than this to complete your revisions, please reply to this message or contact the journal office at plosntds@plos.org. When you're ready to submit your revision, log on to https://www.editorialmanager.com/pntd/ and select the 'Submissions Needing Revision' folder to locate your manuscript file.

Please include the following items when submitting your revised manuscript:

* A letter that responds to each point raised by the editor and reviewer(s). You should upload this letter as a separate file labeled 'Response to Reviewers'. This file does not need to include responses to any formatting updates and technical items listed in the 'Journal Requirements' section below.

* A marked-up copy of your manuscript that highlights changes made to the original version. You should upload this as a separate file labeled 'Revised Manuscript with Track Changes'.

* An unmarked version of your revised paper without tracked changes. You should upload this as a separate file labeled 'Manuscript'.

If you would like to make changes to your financial disclosure, competing interests statement, or data availability statement, please make these updates within the submission form at the time of resubmission. Guidelines for resubmitting your figure files are available below the reviewer comments at the end of this letter.

As the corresponding author, your ORCID iD is verified in the submission system and will appear in the published article. PLOS supports the use of ORCID, and we encourage all coauthors to register for an ORCID iD and use it as well. Please encourage your coauthors to verify their ORCID iD within the submission system before final acceptance, as unverified ORCID iDs will not appear in the published article. Only the individual author can complete the verification step; PLOS staff cannot verify ORCID iDs on behalf of authors.

We look forward to receiving your revised manuscript.

Kind regards,

Guilherme L Werneck

Section Editor

PLOS Neglected Tropical Diseases

Guilherme Werneck

Section Editor

PLOS Neglected Tropical Diseases

Shaden Kamhawi

co-Editor-in-Chief

PLOS Neglected Tropical Diseases

orcid.org/0000-0003-4304-636XX

Paul Brindley

co-Editor-in-Chief

PLOS Neglected Tropical Diseases

orcid.org/0000-0003-1765-0002

**Additional Editor Comments (if provided):**

**Journal Requirements:**

If the reviewer comments include a recommendation to cite specific previously published works, please review and evaluate these publications to determine whether they are relevant and should be cited. There is no requirement to cite these works unless the editor has indicated otherwise.

1) Please ensure that the CRediT author contributions listed for every co-author are completed accurately and in full.

At this stage, the following Authors/Authors require contributions: Chang Xu, Yaru Ji, Qiang Sha, and Kun Li. Please ensure that the full contributions of each author are acknowledged in the "Add/Edit/Remove Authors" section of our submission form.

The list of CRediT author contributions may be found here: https://journals.plos.org/plosntds/s/authorship#loc-author-contributions

2) We do not publish any copyright or trademark symbols that usually accompany proprietary names, eg ©,  ®, or TM  (e.g. next to drug or reagent names). Therefore please remove all instances of trademark/copyright symbols throughout the text, including:

- ® on pages: 12, and 13

- TM on page: 14.

3) Please amend your detailed Financial Disclosure statement. This is published with the article. It must therefore be completed in full sentences and contain the exact wording you wish to be published.

i) State the initials, alongside each funding source, of each author to receive each grant. For example: "This work was supported by the National Institutes of Health (####### to AM; ###### to CJ) and the National Science Foundation (###### to AM)."

ii) State what role the funders took in the study. If the funders had no role in your study, please state: "The funders had no role in study design, data collection and analysis, decision to publish, or preparation of the manuscript."

iii) If any authors received a salary from any of your funders, please state which authors and which funders..

If you did not receive any funding for this study, please simply state: u201cThe authors received no specific funding for this work.u201d

4) Thank you for stating " All raw sequence data from animals were deposited in the NCBI Sequence Read Archive database under accession number: PRJNA1293894, PRJNA1293901 and PRJNA1294146". However we are unable to access the Accession number from the information you provided. Please note that, though access restrictions are acceptable now, your entire data will need to be made freely accessible if your manuscript is accepted for publication.Please review and update the Data Availability Statement to ensure that the underlying data are publicly accessible and that all accession numbers, links, or repository details are correct and functional.

5) We have amended your Competing Interest statement to comply with journal style. We kindly ask that you double check the statement and let us know if anything is incorrect.

6) PLOS now requires that authors provide the original uncropped and unadjusted images underlying all blot or gel results reported in a submission’s figures or Supporting Information files. This policy and the journal’s other requirements for blot/gel reporting and figure preparation are described in detail at https://journals.plos.org/plosone/s/figures#loc-blot-and-gel-reporting-requirements and https://journals.plos.org/plosone/s/figures#loc-preparing-figures-from-image-files.

When you submit your revised manuscript, please ensure that your figures adhere fully to these guidelines and provide the original underlying images for all blot or gel data reported in your submission. See the following link for instructions on providing the original image data: https://journals.plos.org/plosone/s/figures#loc-original-images-for-blots-and-gels.

In your cover letter, please note whether your blot/gel image data are in Supporting Information or posted at a public data repository, provide the repository URL if relevant, and provide specific details as to which raw blot/gel images, if any, are not available. Email us at customercare@plos.org if you have any questions.

**Reviewers' Comments:**

Reviewer's Responses to Questions

**Key Review Criteria Required for Acceptance?**

As you describe the new analyses required for acceptance, please consider the following:

**Methods**

-Are the objectives of the study clearly articulated with a clear testable hypothesis stated?

-Is the study design appropriate to address the stated objectives?

-Is the population clearly described and appropriate for the hypothesis being tested?

-Is the sample size sufficient to ensure adequate power to address the hypothesis being tested?

-Were correct statistical analysis used to support conclusions?

-Are there concerns about ethical or regulatory requirements being met?

Reviewer #1: • Please ensure that the computational workflow is fully reproducible. Critical methodological details are currently missing, including the docking algorithm, grid dimensions, exhaustiveness parameters, validation procedures, and receptor preparation methods.

• The methodological sequence is unclear. The manuscript initially refers to “synthesized derivatives,” but later states that the compounds will be synthesized after the docking analysis. It is therefore unclear how many derivatives were designed, how many were synthesized, which compounds passed the docking screening, and which were subjected to biological evaluation. This represents a major concern in medicinal chemistry studies and should be clarified explicitly.

• The statistical analysis section is unclear. Please clearly indicate the statistical test applied and justify its appropriateness for the data. The section related to microbiota, metabolomics, correlations, and differential abundance analyses lacks sufficient statistical detail. There is no description of the statistical methods used, including FDR correction methods, Benjamini–Hochberg adjustment, or q-values. Similarly, for the correlation analyses, it is unclear whether Pearson or Spearman correlations were applied, and whether multiple testing correction was performed. Please ensure that all statistical approaches are comprehensively described in the data analysis section.

• Please ensure that all synthesized compounds are adequately characterized. The manuscript should explicitly state whether characterization was performed using ^1H NMR, ^13C NMR, and high-resolution mass spectrometry (HRMS). In addition, please report compound purity values

Reviewer #2: (No Response)

Reviewer #3: See the comments under Summary and General Comments

**********

**Results**

-Does the analysis presented match the analysis plan?

-Are the results clearly and completely presented?

-Are the figures (Tables, Images) of sufficient quality for clarity?

Reviewer #1: • It is unclear whether the study included 29 or 30 compounds/components. Please revise the text to ensure consistency throughout the manuscript.

• There is ambiguity regarding the interpretation of the docking results. It is unclear whether the manuscript refers to higher/lower docking scores or binding energy/affinity values. Please define the scoring system clearly and ensure consistent terminology throughout the manuscript.

• “C. parvum” is written inconsistently throughout the text. Please standardize the nomenclature according to journal style guidelines.

• Lines 545–546: “Cryptosporidium-induced intestinal barrier damage and systemic inflammatory responses.” The statement currently implies causality; however, the analyses presented do not appear sufficient to establish a direct causal relationship. Please revise the wording to avoid overinterpretation

Reviewer #2: (No Response)

Reviewer #3: See the comments under Summary and General Comments

**********

**Conclusions**

-Are the conclusions supported by the data presented?

-Are the limitations of analysis clearly described?

-Do the authors discuss how these data can be helpful to advance our understanding of the topic under study?

-Is public health relevance addressed?

Reviewer #1: The conclusion stating that “the two derivatives identified in this study holding significant implications for livestock production and public health” appears overstated considering the methodology and analyses presented. The workflow is difficult to follow, several analytical steps are insufficiently described, and the statistical power supporting these conclusions is unclear.

Reviewer #2: (No Response)

Reviewer #3: See the comments under Summary and General Comments

**********

**Editorial and Data Presentation Modifications?**

Use this section for editorial suggestions as well as relatively minor modifications of existing data that would enhance clarity. If the only modifications needed are minor and/or editorial, you may wish to recommend “Minor Revision” or “Accept”.

Reviewer #1: The methodological sequence need to be revised as describe explicity all analysis performed

Reviewer #2: (No Response)

Reviewer #3: See the comments under Summary and General Comments

**********

**Summary and General Comments**

Use this section to provide overall comments, discuss strengths/weaknesses of the study, novelty, significance, general execution and scholarship. You may also include additional comments for the author, including concerns about dual publication, research ethics, or publication ethics. If requesting major revision, please articulate the new experiments that are needed.

Reviewer #1: (No Response)

Reviewer #2: The manuscript by Xu et al., entitled “ovel inhibitors anti-against Cryptosporidium parvum through blocking its energy metabolism” presents the structure-guided optimization of the previously reported CpLDH inhibitor NSC158011, leading to the identification of two promising derivatives, H25 and H29, with enhanced anti-Cryptosporidium efficacy and reduced toxicity. The work integrates a multidisciplinary strategy encompassing virtual screening, organic synthesis, biochemical and enzymatic characterization, in vitro antiparasitic evaluation, and multiple omics-based analyses to investigate compound activity and mechanism of action. Given the pressing need for new therapeutic options for cryptosporidiosis, this study addresses a highly relevant and potentially impactful research question. Nevertheless, despite these strengths, several substantial concerns regarding the experimental design, data interpretation, and presentation need to be addressed before the manuscript can be considered for publication. Accordingly, I recommend a major revision.

MAJOR COMMENTS

1. While the manuscript demonstrates that three compounds inhibit recombinant CpLDH, it lacks quantitative enzymatic inhibition data. The authors should determine and report the IC₅₀ values of NSC158011, H25, and H29 against recombinant CpLDH, along with complete dose–response curves. Additionally, enzyme kinetic analyses should be conducted to define the mode of inhibition (competitive, noncompetitive, or mixed).

2. The manuscript states that eight compounds exhibited strong inhibitory activity and that fourteen showed favorable properties; however, the complete quantitative screening dataset is not presented. To improve transparency and enable a comprehensive assessment of the screening campaign, the authors should provide the full dataset, including activity measurements and relevant parameters for all evaluated compounds.

3. The manuscript proposes that the lead compounds exert their antiparasitic effects through CpLDH inhibition. However, evidence for direct target engagement is currently limited to molecular docking and in vitro enzyme inhibition assays. To strengthen this mechanistic claim, the authors should perform cellular target engagement via assays such as CETSA (Cellular Thermal Shift Assays) or NanoBRET (Nano-Bioluminescence Resonance Energy Transfer).

4. Computational docking of the lead compounds to hLDH is insufficient to demonstrate selectivity toward CpLDH. The authors should biochemically assess the lead compounds against at least one recombinant human LDH isoform and report selectivity indices.

5. The manuscript repeatedly attributes the observed changes in the microbiome, metabolome, inflammatory responses, and intestinal barrier function to CpLDH inhibition. However, the current data do not establish a causal link between these effects and direct CpLDH inhibition, as they could simply result from reduced parasite burden following treatment. The authors should either provide additional experimental evidence supporting this mechanistic connection or substantially moderate these claims throughout the manuscript.

MINOR COMMENTS

6. The medicinal chemistry strategy is insufficiently described. The authors should provide a clearer explanation of how the 1,440 NSC158011 analogs were generated (including the computational tools, if applicable), as well as the rationale for their prioritization and selection.

7. The authors should report the selectivity index (SI = CC₅₀/IC₅₀) for all tested compounds mentioned in table 1 and clearly define the criteria used to classify compounds as selective. For reference, compounds with an SI > 10 are often considered to have acceptable selectivity.

8. CpLDH is a well-established drug target for cryptosporidiosis, and numerous CpLDH inhibitors have been reported. The authors should expand the Introduction to include key contributions from other groups and better contextualize the novelty of their work within the existing literature.

9. Compound nomenclature should be standardized throughout the manuscript. Inconsistent use of identifiers (e.g., H25 vs. compound 25 and H29 vs. compound 29) may cause confusion and should be corrected for clarity.

10. The manuscript contains numerous grammatical, typographical, and scientific inaccuracies and would benefit from careful English-language editing. Examples include:

(i) Page 3, lines 63–66: The statements “Currently, there are no vaccines or therapeutic drugs specifically targeting cryptosporidiosis” and “Nitazoxanide is the sole FDA-approved drug for treating cryptosporidiosis” are contradictory and should be revised for clarity and accuracy.

(ii) Page 4, lines 102–104: The authors state that “LDH serves as a drug target for Plasmodium, Toxoplasma gondii, Giardia lamblia, Trypanosoma cruzi, and Entamoeba histolytica within the Apicomplexa phylum.” This is incorrect, as Entamoeba histolytica is not an apicomplexan parasite.

(iii) Page 5, line 129: The term “simulated chemical synthesis” is unclear and should be defined or replaced with more appropriate terminology.

(iv) Page 16, lines 438–439: Replace “The active sites of the compounds and protein crystal structures were evaluated using scoring functions” with “The binding affinities of the compounds for the CpLDH active site were evaluated using scoring functions.”

(v) Page 2:

Lines 49–50: Replace “public health officials in 40 states and Puerto Rico have voluntarily annunciated” with “U.S. public health officials in 40 states and Puerto Rico have voluntarily announced.”

Line 53: Replace “Mortality rates can reach 10%-20%” with “Mortality rates can reach 10–20%.”

Line 57: Replace “Cryptosporidium-induced cryptosporidiosis” with “Cryptosporidiosis.”

(vi) Page 3, lines 81–82: Avoid unnecessary capitalization of protein names throughout the manuscript (e.g., zonula occludens-1, claudins, and occludin).

(vii) Page 4:

Line 94: Replace “Unlike other protozoa” with “Unlike other Apicomplexa.”

Line 96: Replace “does not possess apicoplast organelles” with “does not possess an apicoplast.”

Line 109: Replace “lactate dehydrogenase gene” with “LDH,” as the abbreviation has already been introduced.

(viii) Page 8, line 222: Replace “An enzyme activity detection system” with “An enzymatic assay.”

Reviewer #3: The authors describe molecules with potential anti-C. parvum activity that act by inhibiting metabolic pathways, specifically, by inhibiting CpLDH, an enzyme in the glycolytic pathway. Given the scarcity of treatments for cryptosporidiosis, the data presented in the manuscript are significant for the relevant literature. In light of the above, I consider the article approved for the next steps, subject to minor revisions.

- For the title, choose between "anti-" and "against."

- Line 148: Scheme 1 is missing from the manuscript.

- Include abbreviations used in the figures in the figure legends as well.

- Check spelling throughout the text.

**********

PLOS authors have the option to publish the peer review history of their article (what does this mean?). If published, this will include your full peer review and any attached files.

If you choose “no”, your identity will remain anonymous but your review may still be made public.

**Do you want your identity to be public for this peer review?**  For information about this choice, including consent withdrawal, please see our Privacy Policy.

Reviewer #1: No

Reviewer #2: No

Reviewer #3: No

[NOTE: If reviewer comments were submitted as an attachment file, they will be attached to this email and accessible via the submission site. Please log into your account, locate the manuscript record, and check for the action link "View Attachments". If this link does not appear, there are no attachment files.]

**Figure resubmission:** While revising your submission, we strongly recommend that you use PLOS’s NAAS tool (https://ngplosjournals.pagemajik.ai/artanalysis) to test your figure files. NAAS can convert your figure files to the TIFF file type and meet basic requirements (such as print size, resolution), or provide you with a report on issues that do not meet our requirements and that NAAS cannot fix.

After uploading your figures to PLOS’s NAAS tool - https://ngplosjournals.pagemajik.ai/artanalysis, NAAS will process the files provided and display the results in the "Uploaded Files" section of the page as the processing is complete. If the uploaded figures meet our requirements (or NAAS is able to fix the files to meet our requirements), the figure will be marked as "fixed" above. If NAAS is unable to fix the files, a red "failed" label will appear above. When NAAS has confirmed that the figure files meet our requirements, please download the file via the download option, and include these NAAS processed figure files when submitting your revised manuscript.

**Reproducibility:**

To enhance the reproducibility of your results, we recommend that authors of applicable studies deposit laboratory protocols in protocols.io, where a protocol can be assigned its own identifier (DOI) such that it can be cited independently in the future. Additionally, PLOS ONE offers an option to publish peer-reviewed clinical study protocols. Read more information on sharing protocols at https://plos.org/protocols?utm_medium=editorial-email&utm_source=authorletters&utm_campaign=protocols

# Author comment: Author response to Decision Letter 1

**DOI:** 10.1371/journal.pntd.0014742.r002

29 Jul 2026

Attachment Submitted filename: Response.docx

# Peer review: Decision Letter 1

**DOI:** 10.1371/journal.pntd.0014742.r003

**Authors:** Guilherme Werneck

3 Sep 2026

PNTD-D-26-00386R1

Novel inhibitors against Cryptosporidium parvum through blocking its energy metabolism

PLOS Neglected Tropical Diseases

Dear Dr. Li,

Thank you for submitting your manuscript to PLOS Neglected Tropical Diseases. After careful consideration, we feel that it has merit but does not fully meet PLOS Neglected Tropical Diseases's publication criteria as it currently stands. Therefore, we invite you to submit a revised version of the manuscript that addresses the points raised during the review process.

Please submit your revised manuscript within by Nov 02 2026 11:59PM. If you will need more time than this to complete your revisions, please reply to this message or contact the journal office at plosntds@plos.org. When you're ready to submit your revision, log on to https://www.editorialmanager.com/pntd/ and select the 'Submissions Needing Revision' folder to locate your manuscript file.

Please include the following items when submitting your revised manuscript:

* A letter that responds to each point raised by the editor and reviewer(s). You should upload this letter as a separate file labeled 'Response to Reviewers'. This file does not need to include responses to any formatting updates and technical items listed in the 'Journal Requirements' section below.

* A marked-up copy of your manuscript that highlights changes made to the original version. You should upload this as a separate file labeled 'Revised Manuscript with Track Changes'.

* An unmarked version of your revised paper without tracked changes. You should upload this as a separate file labeled 'Manuscript'.

If you would like to make changes to your financial disclosure, competing interests statement, or data availability statement, please make these updates within the submission form at the time of resubmission. Guidelines for resubmitting your figure files are available below the reviewer comments at the end of this letter.

As the corresponding author, your ORCID iD is verified in the submission system and will appear in the published article. PLOS supports the use of ORCID, and we encourage all coauthors to register for an ORCID iD and use it as well. Please encourage your coauthors to verify their ORCID iD within the submission system before final acceptance, as unverified ORCID iDs will not appear in the published article. Only the individual author can complete the verification step; PLOS staff cannot verify ORCID iDs on behalf of authors.

We look forward to receiving your revised manuscript.

Kind regards,

Guilherme L Werneck

Section Editor

PLOS Neglected Tropical Diseases

Guilherme Werneck

Section Editor

PLOS Neglected Tropical Diseases

Shaden Kamhawi

co-Editor-in-Chief

PLOS Neglected Tropical Diseases

orcid.org/0000-0003-4304-636XX

Paul Brindley

co-Editor-in-Chief

PLOS Neglected Tropical Diseases

orcid.org/0000-0003-1765-0002

**Journal Requirements:**

If the reviewer comments include a recommendation to cite specific previously published works, please review and evaluate these publications to determine whether they are relevant and should be cited. There is no requirement to cite these works unless the editor has indicated otherwise.

1) We have noticed that you have uploaded " S1_raw_image" file as supporting information but you have not included it in the list of legends.  Please add a citation of this SI file into the list of legends of your supporting information files.

2) "Please ensure that the CRediT author contributions listed for every co-author are completed accurately and in full.

At this stage, the following Authors require contributions: [ALL AUTHORS]. Please ensure that the full contributions of each author are acknowledged in the ""Add/Edit/Remove Authors"" section of our submission form.

The list of CRediT author contributions may be found here

https://journals.plos.org/plosntds/s/authorship#loc-author-contributions

**Reviewers' Comments:**

Reviewer's Responses to Questions

**Key Review Criteria Required for Acceptance?**

As you describe the new analyses required for acceptance, please consider the following:

**Methods**

-Are the objectives of the study clearly articulated with a clear testable hypothesis stated?

-Is the study design appropriate to address the stated objectives?

-Is the population clearly described and appropriate for the hypothesis being tested?

-Is the sample size sufficient to ensure adequate power to address the hypothesis being tested?

-Were correct statistical analysis used to support conclusions?

-Are there concerns about ethical or regulatory requirements being met?

Reviewer #1: -Are the objectives of the study clearly articulated with a clear testable hypothesis stated?

R= Yes

-Is the study design appropriate to address the stated objectives?

R= Yes

-Is the population clearly described and appropriate for the hypothesis being tested?

R= Yes

-Is the sample size sufficient to ensure adequate power to address the hypothesis being tested?

R= Yes

-Were correct statistical analysis used to support conclusions?

R= Yes

-Are there concerns about ethical or regulatory requirements being met?

R= Yes

All comments and suggestions have been adequately addressed and justified. I have no further comments.

Reviewer #2: See "Summary and General Comments".

**********

**Results**

-Does the analysis presented match the analysis plan?

-Are the results clearly and completely presented?

-Are the figures (Tables, Images) of sufficient quality for clarity?

Reviewer #1: -Does the analysis presented match the analysis plan?

R= Yes

-Are the results clearly and completely presented?

R= Yes

-Are the figures (Tables, Images) of sufficient quality for clarity?

R= Yes

All comments and suggestions have been adequately addressed and justified. I have no further comments.

Reviewer #2: See "Summary and General Comments".

**********

**Conclusions**

-Are the conclusions supported by the data presented?

-Are the limitations of analysis clearly described?

-Do the authors discuss how these data can be helpful to advance our understanding of the topic under study?

-Is public health relevance addressed?

Reviewer #1: -Are the conclusions supported by the data presented?

R= Yes

-Are the limitations of analysis clearly described?

R= Yes

-Do the authors discuss how these data can be helpful to advance our understanding of the topic under study?

R= Yes

-Is public health relevance addressed?

R= Yes

All comments and suggestions have been adequately addressed and justified. I have no further comments.

Reviewer #2: See "Summary and General Comments".

**********

**Editorial and Data Presentation Modifications?**

Use this section for editorial suggestions as well as relatively minor modifications of existing data that would enhance clarity. If the only modifications needed are minor and/or editorial, you may wish to recommend “Minor Revision” or “Accept”.

Reviewer #1: (No Response)

Reviewer #2: See "Summary and General Comments".

**********

**Summary and General Comments**

Use this section to provide overall comments, discuss strengths/weaknesses of the study, novelty, significance, general execution and scholarship. You may also include additional comments for the author, including concerns about dual publication, research ethics, or publication ethics. If requesting major revision, please articulate the new experiments that are needed.

Reviewer #1: (No Response)

Reviewer #2: The revised manuscript has improved substantially, and the authors have adequately addressed most concerns related to study design, workflow presentation, statistical reporting, terminology, and data interpretation. The inclusion of a limitations section and the more cautious treatment of causal inferences are particularly appreciated.

While advanced target engagement studies such as CETSA may reasonably be considered beyond the scope of the current revision, quantitative enzyme inhibition data (IC₅₀ values) against both CpLDH and hLDH should be performed to support the proposed mechanism and assess target selectivity, building directly on the recombinant enzyme assays already reported in the manuscript.

Second, the chemical characterization remains incomplete. Partial characterization data were provided only for NSC158011, H25, and H29, whereas the remaining synthesized compounds were not comprehensively characterized. For a medicinal chemistry study, appropriate structural confirmation using techniques such as 1H NMR, 13C NMR, HRMS, and purity assessment should be provided for all synthesized compounds.

If additional biochemical studies are beyond the scope of the current revision, the manuscript would benefit from a clearer distinction between evidence supporting target association and evidence required for definitive target validation.

**********

PLOS authors have the option to publish the peer review history of their article (what does this mean?). If published, this will include your full peer review and any attached files.

If you choose “no”, your identity will remain anonymous but your review may still be made public.

**Do you want your identity to be public for this peer review?**  For information about this choice, including consent withdrawal, please see our Privacy Policy.

Reviewer #1: No

Reviewer #2: No

[NOTE: If reviewer comments were submitted as an attachment file, they will be attached to this email and accessible via the submission site. Please log into your account, locate the manuscript record, and check for the action link "View Attachments". If this link does not appear, there are no attachment files.]

**Figure resubmission:** While revising your submission, we strongly recommend that you use PLOS’s NAAS tool (https://ngplosjournals.pagemajik.ai/artanalysis) to test your figure files. NAAS can convert your figure files to the TIFF file type and meet basic requirements (such as print size, resolution), or provide you with a report on issues that do not meet our requirements and that NAAS cannot fix.

After uploading your figures to PLOS’s NAAS tool - https://ngplosjournals.pagemajik.ai/artanalysis, NAAS will process the files provided and display the results in the "Uploaded Files" section of the page as the processing is complete. If the uploaded figures meet our requirements (or NAAS is able to fix the files to meet our requirements), the figure will be marked as "fixed" above. If NAAS is unable to fix the files, a red "failed" label will appear above. When NAAS has confirmed that the figure files meet our requirements, please download the file via the download option, and include these NAAS processed figure files when submitting your revised manuscript.

**Reproducibility:**

To enhance the reproducibility of your results, we recommend that authors of applicable studies deposit laboratory protocols in protocols.io, where a protocol can be assigned its own identifier (DOI) such that it can be cited independently in the future. Additionally, PLOS ONE offers an option to publish peer-reviewed clinical study protocols. Read more information on sharing protocols at https://plos.org/protocols?utm_medium=editorial-email&utm_source=authorletters&utm_campaign=protocols

# Author comment: Author response to Decision Letter 2

**DOI:** 10.1371/journal.pntd.0014742.r004

3 Sep 2026

Attachment Submitted filename: Response letter.docx

# Peer review: Decision Letter 2

**DOI:** 10.1371/journal.pntd.0014742.r005

**Authors:** Guilherme Werneck

10 Sep 2026

Dear prof Li,

We are pleased to inform you that your manuscript 'Novel inhibitors against Cryptosporidium parvum through blocking its energy metabolism' has been provisionally accepted for publication in PLOS Neglected Tropical Diseases.

Before your manuscript can be formally accepted you will need to complete some formatting changes, which you will receive in a follow up email. A member of our team will be in touch with a set of requests.

Please note that your manuscript will not be scheduled for publication until you have made the required changes, so a swift response is appreciated.

IMPORTANT: The editorial review process is now complete. PLOS will only permit corrections to spelling, formatting or significant scientific errors from this point onwards. Requests for major changes, or any which affect the scientific understanding of your work, will cause delays to the publication date of your manuscript.

Should you, your institution's press office or the journal office choose to press release your paper, you will automatically be opted out of early publication. We ask that you notify us now if you or your institution is planning to press release the article. All press must be co-ordinated with PLOS.

Thank you again for supporting Open Access publishing; we are looking forward to publishing your work in PLOS Neglected Tropical Diseases.

Best regards,

Guilherme L Werneck

Section Editor

PLOS Neglected Tropical Diseases

Shaden Kamhawi

co-Editor-in-Chief

PLOS Neglected Tropical Diseases

orcid.org/0000-0003-4304-636XX

Paul Brindley

co-Editor-in-Chief

PLOS Neglected Tropical Diseases

orcid.org/0000-0003-1765-0002

***********************************************************

Reviewer's Responses to Questions

**Key Review Criteria Required for Acceptance?**

As you describe the new analyses required for acceptance, please consider the following:

**Methods**

-Are the objectives of the study clearly articulated with a clear testable hypothesis stated?

-Is the study design appropriate to address the stated objectives?

-Is the population clearly described and appropriate for the hypothesis being tested?

-Is the sample size sufficient to ensure adequate power to address the hypothesis being tested?

-Were correct statistical analysis used to support conclusions?

-Are there concerns about ethical or regulatory requirements being met?

Reviewer #2: (No Response)

**********

**Results**

-Does the analysis presented match the analysis plan?

-Are the results clearly and completely presented?

-Are the figures (Tables, Images) of sufficient quality for clarity?

Reviewer #2: (No Response)

**********

**Conclusions**

-Are the conclusions supported by the data presented?

-Are the limitations of analysis clearly described?

-Do the authors discuss how these data can be helpful to advance our understanding of the topic under study?

-Is public health relevance addressed?

Reviewer #2: (No Response)

**********

**Editorial and Data Presentation Modifications?**

Use this section for editorial suggestions as well as relatively minor modifications of existing data that would enhance clarity. If the only modifications needed are minor and/or editorial, you may wish to recommend “Minor Revision” or “Accept”.

Reviewer #2: (No Response)

**********

**Summary and General Comments**

Use this section to provide overall comments, discuss strengths/weaknesses of the study, novelty, significance, general execution and scholarship. You may also include additional comments for the author, including concerns about dual publication, research ethics, or publication ethics. If requesting major revision, please articulate the new experiments that are needed.

Reviewer #2: (No Response)

**********

PLOS authors have the option to publish the peer review history of their article (what does this mean?). If published, this will include your full peer review and any attached files.

If you choose “no”, your identity will remain anonymous but your review may still be made public.

**Do you want your identity to be public for this peer review?**  For information about this choice, including consent withdrawal, please see our Privacy Policy.

Reviewer #2: No

# Editor report: Acceptance letter

**DOI:** 10.1371/journal.pntd.0014742.r006

**Authors:** Guilherme Werneck

Dear prof Li,

We are delighted to inform you that your manuscript, "Novel inhibitors against Cryptosporidium parvum through blocking its energy metabolism," has been formally accepted for publication in PLOS Neglected Tropical Diseases.

We have now passed your article onto the PLOS Production Department who will complete the rest of the publication process. All authors will receive a confirmation email upon publication.

The corresponding author will soon be receiving a typeset proof for review, to ensure errors have not been introduced during production. Please review the PDF proof of your manuscript carefully, as this is the last chance to correct any scientific or type-setting errors. Please note that major changes, or those which affect the scientific understanding of the work, will likely cause delays to the publication date of your manuscript. Note: Proofs for Front Matter articles (Editorial, Viewpoint, Symposium, Review, etc...) are generated on a different schedule and may not be made available as quickly.

Soon after your final files are uploaded, the early version of your manuscript will be published online unless you opted out of this process. The date of the early version will be your article's publication date. The final article will be published to the same URL, and all versions of the paper will be accessible to readers.

For Research Articles, you will receive an invoice from PLOS for your publication fee after your manuscript has reached the completed accept phase. If you receive an email requesting payment before acceptance or for any other service, this may be a phishing scheme. Learn how to identify phishing emails and protect your accounts at https://explore.plos.org/phishing.

Thank you again for supporting open-access publishing; we are looking forward to publishing your work in PLOS Neglected Tropical Diseases.

Best regards,

Shaden Kamhawi

co-Editor-in-Chief

PLOS Neglected Tropical Diseases

Paul Brindley

co-Editor-in-Chief

PLOS Neglected Tropical Diseases
